# A palmitate-rich metastatic niche enables metastasis growth via p65 acetylation resulting in pro-metastatic NF-κB signaling

**DOI:** 10.1038/s43018-023-00513-2

**Published:** 2023-02-02

**Authors:** Patricia Altea-Manzano, Ginevra Doglioni, Yawen Liu, Alejandro M. Cuadros, Emma Nolan, Juan Fernández-García, Qi Wu, Mélanie Planque, Kathrin Julia Laue, Florencia Cidre-Aranaz, Xiao-Zheng Liu, Oskar Marin-Bejar, Joke Van Elsen, Ines Vermeire, Dorien Broekaert, Sofie Demeyer, Xander Spotbeen, Jakub Idkowiak, Aurelie Montagne, Margherita Demicco, H Furkan Alkan, Nick Rabas, Carla Riera-Domingo, François Richard, Tatjana Geukens, Maxim De Schepper, Sophia Leduc, Sigrid Hatse, Yentl Lambrechts, Emily Jane Kay, Sergio Lilla, Alisa Alekseenko, Vincent Geldhof, Bram Boeckx, Celia de la Calle Arregui, Giuseppe Floris, Johannes V. Swinnen, Jean-Christophe Marine, Diether Lambrechts, Vicent Pelechano, Massimiliano Mazzone, Sara Zanivan, Jan Cools, Hans Wildiers, Véronique Baud, Thomas G.P. Grünewald, Uri Ben-David, Christine Desmedt, Ilaria Malanchi, Sarah-Maria Fendt

**Affiliations:** 1 Laboratory of Cellular Metabolism and Metabolic Regulation, VIB-KU Leuven Center for Cancer Biology, VIB, Herestraat 49, 3000 Leuven, Belgium; 2 Laboratory of Cellular Metabolism and Metabolic Regulation, Department of Oncology, KU Leuven and Leuven Cancer Institute (LKI), Herestraat 49, 3000 Leuven, Belgium; 3 Department of Gastroenterology, Affiliated Hospital of Jiangsu University, Jiangsu University, Zhenjiang, China, 212001; 4 The Francis Crick Institute. 1 Midland Road, London NW1 1AT, United Kingdom; 5 Laboratory of Experimental Oncology, Department of Oncology, KU Leuven. Herestraat 49 - box 815 3000 Leuven, Belgium; 6 Department of Human Molecular Genetics & Biochemistry, Faculty of Medicine, Tel Aviv University, 69978 Tel Aviv, Israel; 7 Hopp-Children’s Cancer Center (KiTZ), Heidelberg, Germany; 8 Division of Translational Pediatric Sarcoma Research, German Cancer Research Center (DKFZ), German Cancer Consortium (DKTK) Im Neuenheimer Feld 280, 69120 Heidelberg, Germany; 9 Laboratory for Molecular Cancer Biology, Center for Cancer Biology, VIB; Laboratory for Molecular Cancer Biology, Department of Oncology, KU Leuven. Herestraat 49 - box 912 3000 Leuven, Belgium; 10 Laboratory for molecular biology of leukemia (VIB-KULeuven) Herestraat 49, Box 912 ON IV, 3000 Leuven Belgium; 11 Laboratory of Lipid Metabolism and Cancer, Department of Oncology, KU Leuven. Herestraat 49 - box 825 3000 Leuven, Belgium; 12 Department of Analytical Chemistry, Faculty of Chemical Technology, University of Pardubice, 53210 Pardubice, Czech Republic; 13 Université Paris Cité, NF-kappaB, Différenciation et Cancer, F-75006 Paris, France; 14 Laboratory of Tumor Inflammation and Angiogenesis, Center for Cancer Biology, Department of Oncology, KU Leuven, Herestraat 49 - box 912 3000 Leuven Belgium; 15 Laboratory for Translational Breast Cancer Research, Department of Oncology, Herestraat 49 - box 815 3000 Leuven, Belgium; 16 Cancer Research UK Beatson Institute. G61 1BD, Glasgow, UK; 17 SciLifeLab, Department of Microbiology, Tumor and Cell Biology. Karolinska Institute, Tomtebodavägen 23A, Box 1031, 171 65 Solna, Sweden; 18 Laboratory for angiogenesis and vascular metabolism (VIB-KULeuven) ON IV, 3000 Leuven Belgium; 19 Laboratory of Translational Genetics, VIB Center for Cancer Biology, 3000 Leuven, Belgium; 20 Laboratory of Translational Genetics, Department of Human Genetics, KU Leuven, 3000 Leuven, Belgium; 21 Department of Imaging and Pathology, Laboratory of Translational Cell & Tissue Research and University Hospitals Leuven; Department of Pathology, KU Leuven, Herestraat 49, Box 912 ON IV, 3000 Leuven Belgium; 22 Institute of Cancer Sciences, University of Glasgow, G611QH, Glasgow, UK; 23 Institute of Pathology, Heidelberg University Hospital, Im Neuenheimer Feld 224, 69120 Heidelberg Germany

**Keywords:** metastasis formation, fatty acids, palmitate, CPT1a, acetylation, NF-κB, p65, RelA, KAT2a, GCN5, breast cancer, pre-metastatic niche, AT2, high fat diet

## Abstract

Metabolic rewiring is often considered an adaptive pressure limiting metastasis formation. However, some nutrients available in distant organs may inherently promote metastatic growth. We find that the lung and liver are lipid-rich environments. Moreover, we observe that premetastatic niche formation increases palmitate availability only in the lung, while high fat diet increases it in both organs. In line, targeting palmitate processing inhibits breast cancer-derived lung metastasis formation. Mechanistically, breast cancer cells use palmitate to synthesize acetyl-CoA in a carnitine palmitoyltransferase 1a (CPT1a)-dependent manner. Concomitantly, lysine acetyltransferase 2a (KAT2a) expression is promoted by palmitate, linking the available acetyl-CoA to the acetylation of the NF-κB subunit p65. Deletion of KAT2a or CPT1a reduces metastasis formation in lean and high fat diet mice and patient-derived metastases from lipid rich organs show a co-expression of both proteins. In conclusion, palmitate-rich environments foster metastases growth by increasing p65 acetylation resulting in a pro-metastatic NF-κB signaling.

Nutrient availability is a key aspect of a permissive environment enabling metastasis formation. Certain nutrients such as glucose, fatty acids, pyruvate and glutamine are aiding metastasizing cancer cells when they are seeding and colonizing in a distant organ^1^. While nutrient availability is certainly defined by the functional processes occurring in healthy organs, the question arises whether aberrant disease processes or dietary conditions influence the nutrient concentrations in organs of metastasis. A nutrient class highly linked to metastasis formation are fatty acids^2^. In many cancer types, blocking fatty acid uptake is sufficient to impair metastasis formation ^3,4^, while increased dietary fat intake promotes cancer progression^1,2^. However, it remains largely elusive whether these fatty acids are available in future organs of metastasis and whether high fat diet feeding alters their concentration. Another aberrant disease process linked to cancer progression is pre-metastatic niche formation^5^. There is extensive evidence that the immune cell and extracellular matrix composition of the pre-metastatic niche are modulated by tumor secreted factors resulting in increased metastasis formation in such primed organs ^5,6^. However, to date very little is known about nutrient priming of the premetastatic niche with only one report showing increased glucose availability resulting from a general hypometabolism of lung resident cells ^7^.

We found that the interstitial fluid of lungs and livers is palmitate-rich, and that high fat diet and premetastatic niche formation further increase palmitate concentrations in an organs-specific or general manner. We further show that breast cancer cells rely on CPT1a for fatty acid oxidation, which in turn activates NF-κB signaling via the acetylation of p65 in a KAT2a-dependent manner. Accordingly, CPT1a and KAT2a targeting is highly effective in inhibiting metastasis formation.

## Results

### The lung and liver are lipid-rich environments

**Extended Data Figure 1:**
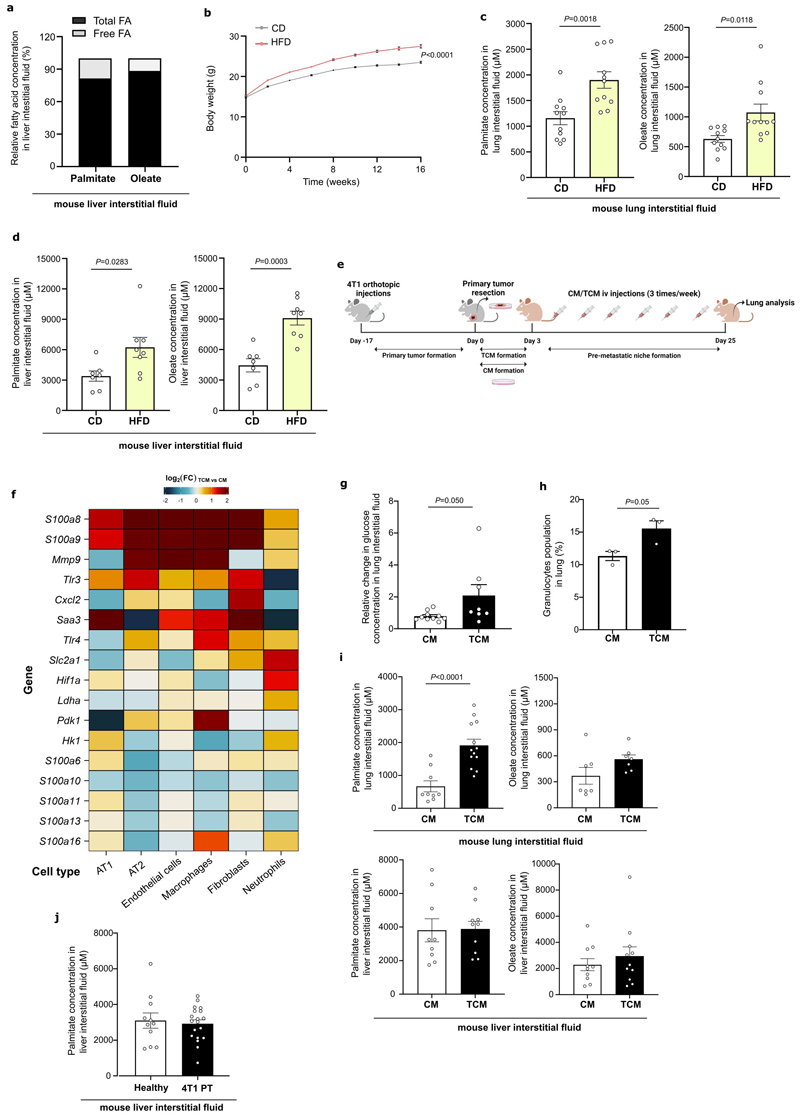
Palmitate and oleate levels are increased upon high fat diet exposure, and only palmitate is released by AT2 cells during pre-metastatic niche formation a. Fraction of free fatty acids (grey) over total fatty acid content (black) of liver interstitial fluid of healthy BALB/c mice (n=7). b. Mouse weight gain upon high fat or control diet over the course of the experiment. Data are presented as mean ± SEM(n =60). Mixed-effects analysis with Sidak’s multiple comparisons. Asterisks represent statistical significance as follows: ***p < 0.001, ****p < 0.0001. c. Palmitate and oleate abundance in lung interstitial fluid of BALB/c mice after 16 weeks on control (CD) or high fat (HFD) diet (n=11). Data are presented as mean ± SEM of absolute concentration measured by mass spectrometry. Unpaired two-tailed t-tests with Welch correction. d. Palmitate and oleate abundance in liver interstitial fluid of BALB/c mice after 16 weeks on CD or HFD diet (n=8). Data are presented as mean ± SEM of absolute concentration measured by mass spectrometry. Unpaired two-tailed t-tests with Welch correction. e. Schematic illustration for experimental pre-metastatic niche formation procedure. CM, control media; TCM, tumor conditioned media; i.v. intravenous. f. Changes in gene expression in lung populations upon TCM injection relative to CM injections, for genes whose upregulation has been previously linked to pre-metastatic niche formation, such as S100a8, S100a9, Mmp9 90,91 and Tlr3 and Cxcl2 in lung alveolar type II cells 91, Tlr4 and Saa3 in lung endothelial cells and macrophages 92; Slc2a1, Pdk1, and Ldha in macrophages 93, and S100 genes in lung fibroblasts 94. The color scale denotes log2 fold changes in TCM vs. CM. g. Relative glucose concentration in the lung interstitial fluid of healthy BALB/c mice exposed to control media or tumor condition media. Data are presented as mean ± SEM(n ≥7). Unpaired two-tailed t-tests with Welch correction. h. Granulocytes population (which includes neutrophils) present in lungs after induction of pre-metastatic niche formation using tumor conditioned media or control media. Data are presented as mean ± SEM (n=3). Unpaired two-tailed t-tests with Welch correction. i. Palmitate and oleate abundance in lung (n=16) and liver (n ≥7) interstitial fluid of BALB/c mice injected with control media (CM) or 4T1-tumor conditioned media (TCM) (3 weeks, 3 times/week). Data are presented as mean ± SEM. Unpaired two-tailed t-tests with Welch correction. j. Palmitate concentration in liver interstitial fluid of healthy or 4T1 tumor-bearing (PT) BALB/c mice. Data are presented as mean ± SEM (n ≥15).

**Figure 1:**
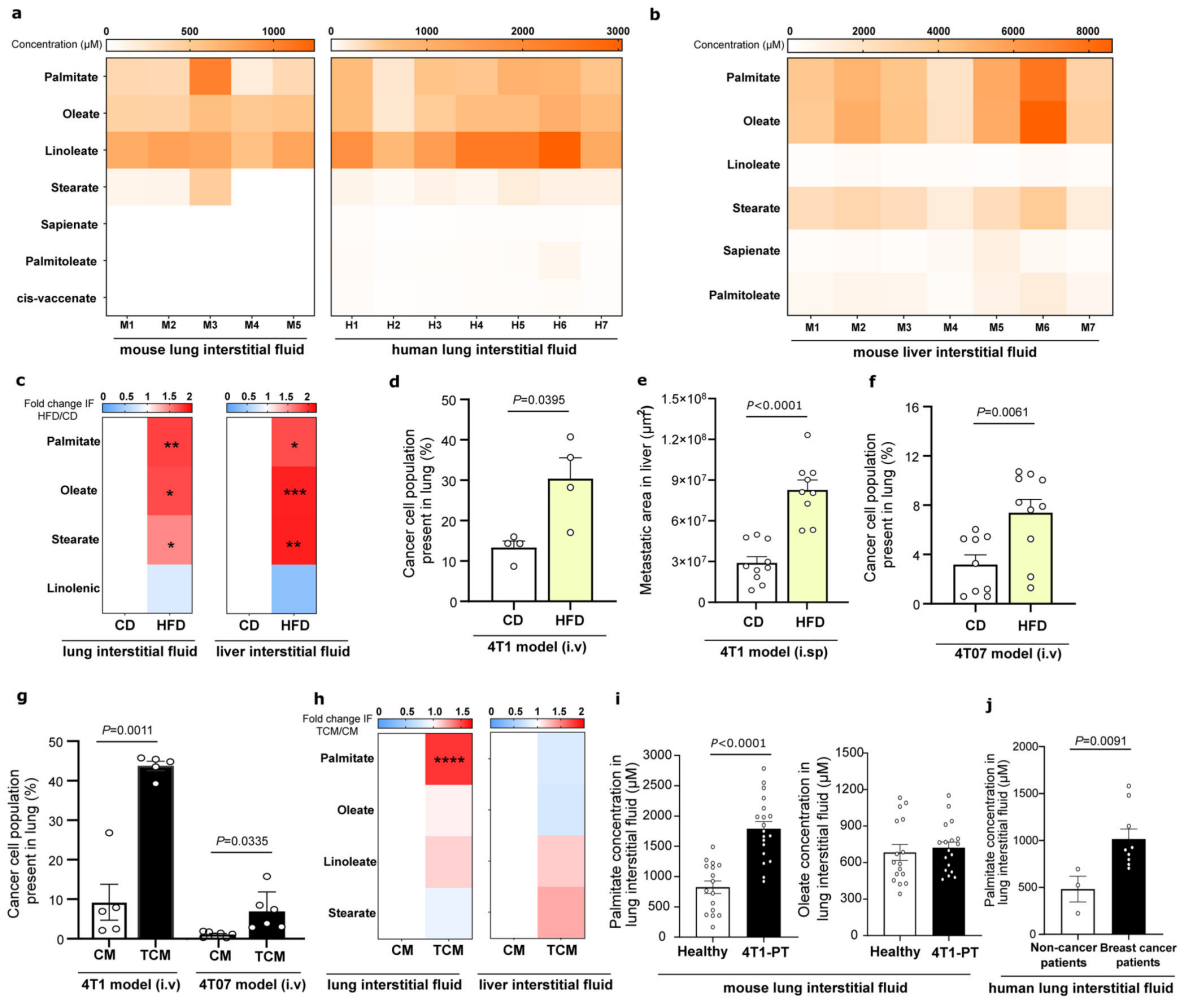
High fat diet enhances overall fatty acid availability in the lung and liver while palmitate availability is specifically increased only in lung during pre-metastatic niche formation. **a.** Total fatty acid concentrations in the lung interstitial fluid of healthy BALB/c mice (n=5) and human non-cancer patients (n=7) detected by mass spectrometry. **b.** Total fatty acid concentrations present in the liver interstitial fluid of healthy BALB/c mice (n=7) detected by mass spectrometry. **c.** Relatives changes in fatty acid concentrations in lung and liver interstitial fluid of BALB/c mice after 16 weeks on control diet (CD,) or high fat diet (HFDs) (n≥10). Unpaired two-tailed t-tests with Welch correction.*P<0.05, **P<0.01, ***P<0.001. **d.** Percentage of cancer cells present in lung of CD and HFD feeding mice injected with CD90.1 expressing 4T1 breast cancer cells intravenously (i.v.). Data are presented as mean + SEM(n=4). Unpaired two-tailed t-tests with Welch correction. **e.** Metastatic area in liver of CD and HFD feeding mice injected with 4T1 breast cancer cells intrasplenic (i.sp.). Data are presented as mean + SEM (n≥9). Unpaired two-tailed t-tests with Welch correction. **f.** Percentage of cancer cells present in lung of CD and HFD feeding mice injected with CD90.1 expressing 4T07 breast cancer cells intravenously (i.v.). Data are presented as mean + SEM (n≥9). Unpaired two-tailed t-tests with Welch correction. **g.** Percentage of cancer cells present in lung of mice injected with control media (CM) or 4T1-tumor conditioned media (TCM) (3 weeks, 3 times/week) after 16 days of intravenous (i.v.) injections with CD90.1 expressing 4T1 or 4T07 breast cancer cells. Data are presented as mean ± SEM (n≥5). Unpaired two-tailed t-tests with Welch correction. **h.** Relatives changes in fatty acid concentrations in lung and liver interstitial fluid of BALB/c mice injected with CM or 4T1- TCM (n≥11). Unpaired two-tailed t-tests with Welch correction., ****P<0.0001. **i.** Palmitate (left panel) and oleate (right panel) concentration in lung interstitial fluid of healthy or 4T1 tumor-bearing (PT) BALB/c mice. Data are presented as mean ± SEM (n ≥15). Unpaired two-tailed t-tests with Welch correction. **j.** Palmitate concentration in lung interstitial fluid of non-cancer (emphysema) patients (n=3) compared to breast cancer patients without detected lung metastases (n=9). Data are presented as mean ± SEM. Unpaired two-tailed Mann–Whitney U-tests.

Although the lung and liver are frequent metastatic sites, nutrient concentrations in these organs remain largely unknown. We isolated lung and liver interstitial fluid, which is a local source of nutrients for metastasized cancer cells, from BALB/c mice and non-cancerous lung tissue of patients (Supplementary Table 1) ^8,9^ and measured fatty acid availability using mass spectrometry. Mouse and human lung interstitial fluids were very similar regarding fatty acid composition and concentrations (Figure 1a). Palmitate and oleate were high in both organs, while linoleate and stearate were highly available in lung and liver, respectively (Figure 1a-b). Most of these fatty acids were present as acyl side chains within lipids (referred as fatty acids) rather than their free form (Extended Data Figure 1a). Thus, we concluded that the lung and the liver are organs rich in palmitate and oleate containing lipids.

### High fat diet increases general fatty acid availability

To date, it is largely unknown whether high fat diet exposure alters nutrient concentrations in organ interstitial fluids. Thus, we investigated fatty acid concentrations in intestinal fluids and metastasis formation in BALB/c mice fed a lipid-rich diet (HFD) or a control diet (CD) for 16 weeks (Extended Data Figure 1b). We found that HFD feeding increased the levels of palmitate, oleate and stearate in lung and liver interstitial fluid (Figure 1c, Extended Data Figure 1c-d). Next, we asked whether this elevation in fatty acid availability coincided with an increase in metastasis formation. For lung and liver metastasis, we intravenously (i.v.) or intrasplenically (i.sp.) injected 4T1 cells expressing the congenic marker CD90.1 and measured the percentage of cancer cells in the lung after 12 days or metastatic area in the liver after 17 days. We found that HFD feeding increased lung and liver metastatic growth by more than two- and three-fold, respectively (Figure 1d-e). Next, we asked whether HFD feeding was also sufficient to increase the metastatic growth of 4T07 cells, which are known to disseminate to the lung but do not outgrow ^10^. Remarkably, we observed that 4T07 cells were more proficient to colonize the lung environment in mice that received a HFD compared to mice on CD (Figure 1f). Thus, we concluded that HFD exposure increased fatty acid availability in lung and liver and that highly and low metastatic cancer cells benefit from an organ environment primed by HFD.

### Pre-metastatic lungs are enriched in palmitate

Next, we investigated whether pre-metastatic niche formation induced by tumor secreted factors ^6^ results in fatty acid priming. We generated tumor-conditioned media (TCM) by culturing 4T1 primary breast tumors in DMEM media without FBS ^11–14^ and injected TCM or control media (CM) for three weeks intravenously to BALB/c mice ^13,14^ (Extended Data Figure 1e). We confirmed that TCM injections induced an experimental pre-metastatic niche in the lung (Extended Data Figure 1f-h) and boosted lung metastasis formation (Figure 1g). Likewise, 4T07 cells injected into mice that had received 4T1 tumor-derived TCM resulted in a 6.8-fold increase in metastasis formation (Figure 1g). Consecutively, we isolated lung and liver interstitial fluid from mice treated with TCM or CM and measured fatty acid concentrations. We discovered that upon experimental pre-metastatic niche formation palmitate, but not oleate or linoleate, abundance increased in lung interstitial fluid. On the contrary, fatty acid abundance was not significantly changed in the liver (Figure 1h, Extended Data Figure 1i). To verify this finding, we injected 4T1 cancer cells into the mammary fat pad and isolated lung and liver interstitial fluid before detectable spontaneous metastases arose (17 days). We found that also spontaneous pre-metastatic niche formation resulted in the same fatty acid changes as TCM injection (Figure 1i, Extended Data Figure 1j). Next, we analyzed lung interstitial fluid collected *post-mortem* from patients with breast cancer and control patients without cancer (Supplementary Table 1-2). In line with our mouse data, we found that breast cancer patients without detected lung metastases showed increased palmitate but not oleate concentration in their lung interstitial fluid compared to control patients without cancer (Figure 1j). Thus, we concluded that specifically palmitate concentrations increase in the lung during pre-metastatic niche formation.

### AT2 cells secrete palmitate lipids to pre-metastatic lungs

**Extended Data Figure 2:**
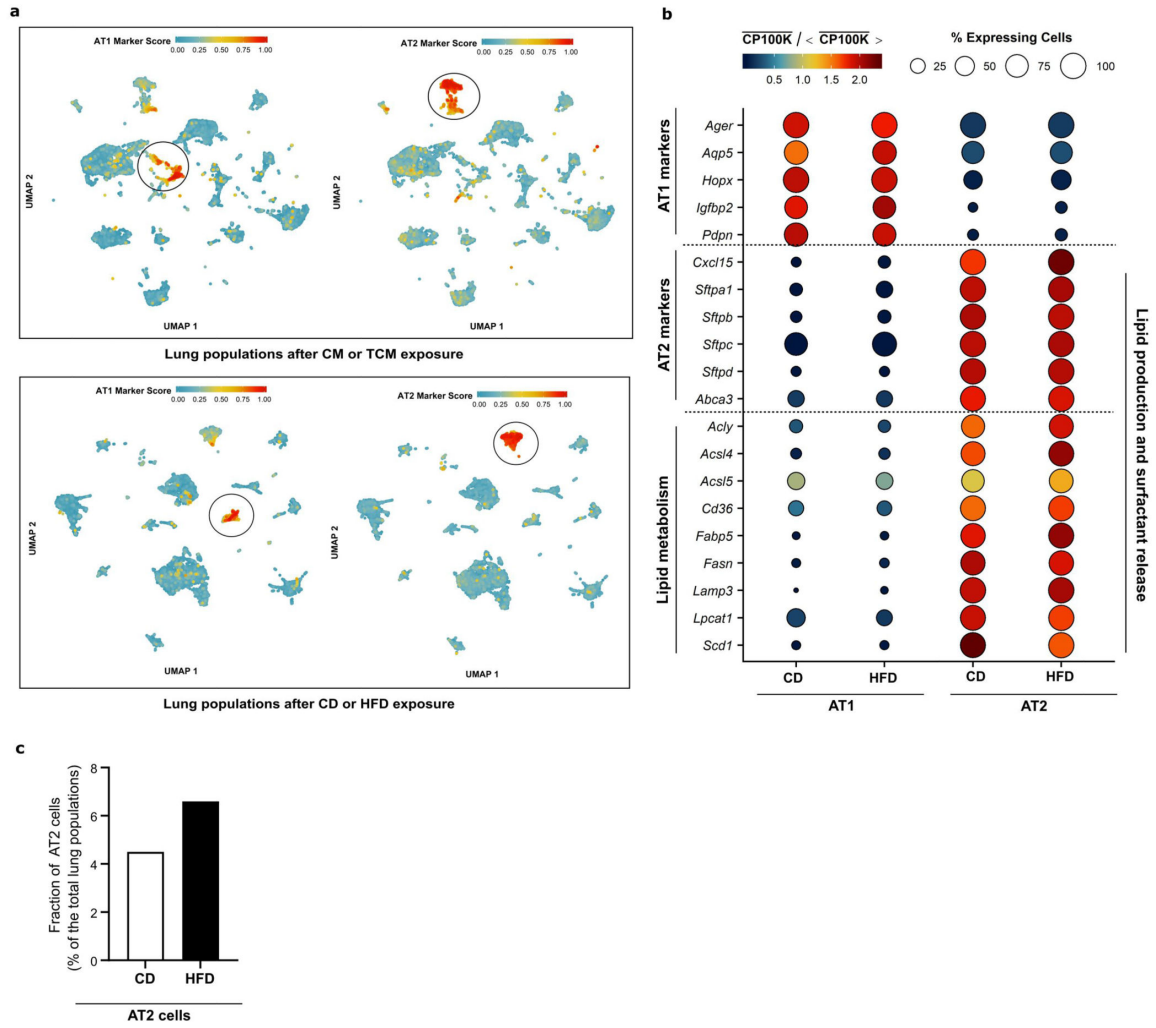
High fat diet moderately impacts gene expression but increases the fraction of lung resident alveolar type II cells a. UMAP plots for the scRNA-seq data corresponding to lungs preconditioned with control media (CM) and tumor conditioned media (TCM), or control diet (CD) and high fat diet (HFD). Color-coded based on GSVA-based marker scores for gene sets corresponding to alveolar type I (AT1) and II (AT2) marker genes. Identified clusters are indicated within black circles. Marker scores are scaled to the range 0–1 for each market set (see Methods). b. scRNA-seq-based gene expression vs. cell type and diet condition profiles for known marker for AT1 and AT2 cells and lipid-related genes indicated on the left-hand side. Scaled expression levels are indicated by the color scale, where (CP100k) ¯ denotes the average gene expression level (in counts per 100k reads) over all cells of a given type in each condition, and (CP100k) ¯ the average of the latter over all cell types and condition media. The areas of the circles represent the percentage of cells with non-zero expression of each gene among all cells of each type and in each dietary condition. CD, control diet; HFD, high fat diet. c. Fractions of cells corresponding to AT2 cells, among all cells present in the lung of mice exposed to control (CD) or high fat (HFD) diet, as determined from scRNA-seq data.

**Figure 2:**
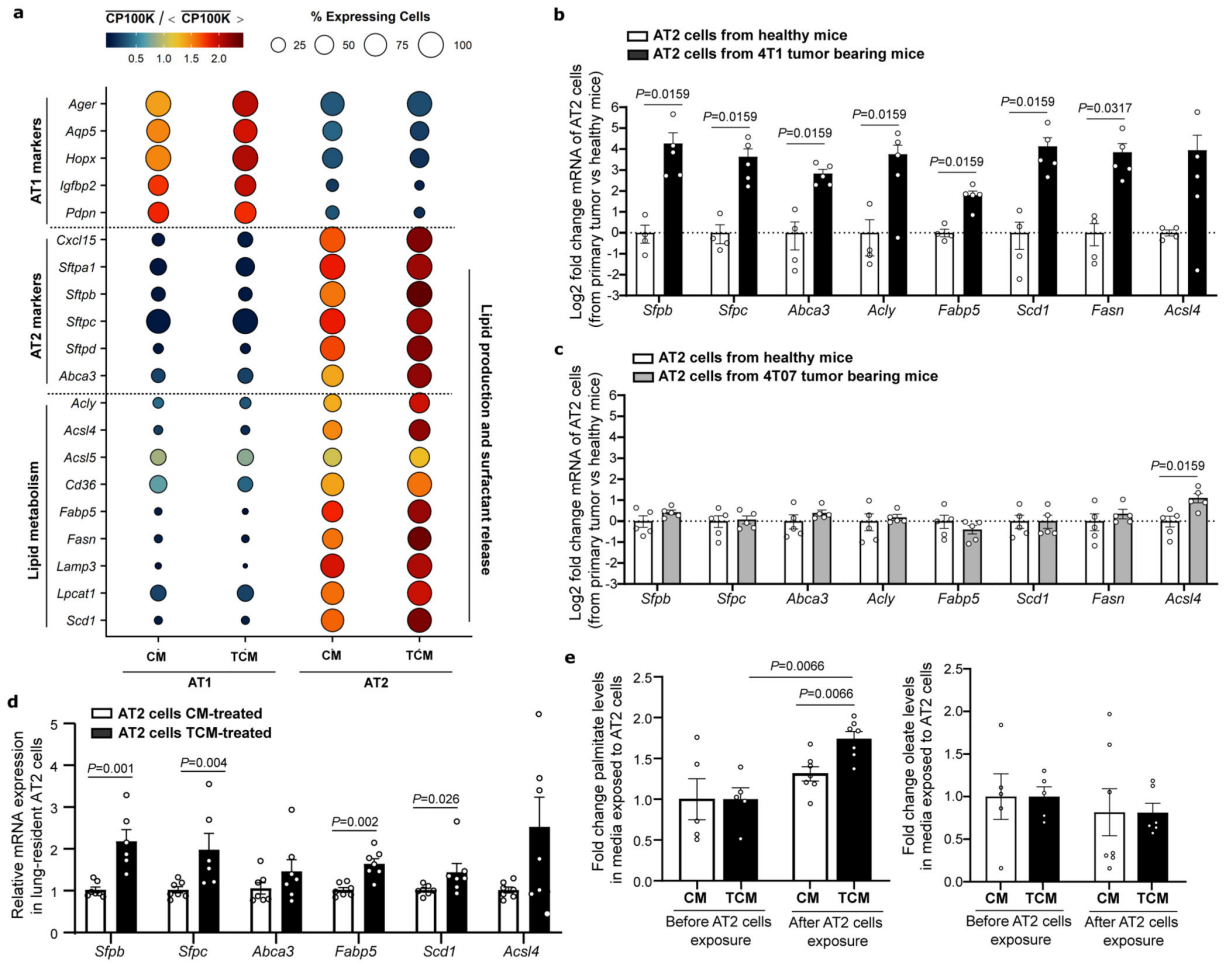
Lung resident alveolar type II (AT2) cells respond to preconditioning of metastatic breast primary tumors by increasing surfactant-related genes and palmitate release. **a**. scRNA-seq-based gene expression vs. cell type and pre-conditioning media profiles for known marker for AT1 and AT2 cells and lipid-related genes indicated on the left-hand side. Scaled expression levels are indicated by the color scale, where $\bar{CP100k}$ denotes the average gene expression level (in counts per 100k reads) over all cells of a given type in each condition, and $\bar{CP100k}$the average of the latter over all cell types and pre-conditioning media conditions. The areas of the circles represent the percentage of cells with non-zero expression of each gene among all cells of each type and in each preconditioning media condition. CM, control media; TCM, tumor conditioned media.. **b-c**. Relative expression of genes implicated in lipid and production surfactant release in AT2 cells isolated from healthy or **(b)** 4T1 and **(c)** 4T07 tumor-bearing (PT) BALB/c mice. Bars represent log2 of average fold change relative to AT2 in healthy mice and single dots represent individual fold changes. Error bars represent mean ± SEM (n=5). Unpaired nonparametric two-tailed Mann–Whitney U-tests. **d**. Relative expression of genes involved in pulmonary surfactant production and secretion in alveolar type II (AT2) cells exposed to control (CM) or tumor conditioned media (TCM) for 72h. Data are presented as mean ± SEM (n=6). Unpaired nonparametric two-tailed Mann–Whitney U-tests. **e**. Relative palmitate and oleate levels present in control (CM) or tumor conditioned media (TCM) before and after exposure to lung resident AT2 cells for 72h. Data are shown as mean ± SEM of fold changes compared with levels in media before incubation with AT2 cells (n=6). One-way ANOVA with Tukey’s multiple comparison test.

Next, we hypothesized that some lung resident cells respond to tumor secreted factors by releasing palmitate to the pre-metastatic niche. To identify these cells, we performed single cell RNA sequencing (scRNA-seq) on lungs of BALB/c mice that were injected with TCM or CM. Cell type annotation combined with the analysis of lipid related genes highlighted alveolar type II (AT2) as the potential cell type increasing lipid release in response to TCM (Figure 2a, Extended Data Figure 2a). AT2 resident cells are known to naturally release lipids that form 90% of the pulmonary surfactant in healthy lungs ^15^. Accordingly, we observed that the expression of lipid production and pulmonary surfactant release genes was upregulated in AT2 cells in mice exposed to TCM (Figure 2a). The expression of the same genes remained unchanged in alveolar type I epithelial (AT1) cells (Figure 2a). Similarly, AT2 cells freshly isolated from mice undergoing spontaneous pre-metastatic niche formation due to 4T1 primary breast tumors showed a significantly elevated expression of the same lipid production and pulmonary surfactant release genes (Figure 2b). Importantly, the expression of these genes (except for *Acsl4*) was not significantly altered in the presence of a low metastatic 4T07 primary breast tumor (Figure 2c).

We next investigated whether AT2 cells directly respond to TCM and potentially elevate palmitate secretion. We isolated AT2 cells from *Sftpc-CreERT2;R26R-YFP* mice, cultured them on a 3D-scaffold system ^16^ and treated them with CM or TCM generated from 4T1 primary tumors. We observed that AT2 cells increased the expression of several genes, identified by our *in vivo* scRNA-seq, after *in vitro* TCM exposure (Figure 2d). Moreover, we observed an elevated secretion of palmitate, but not oleate, from AT2 cells treated with TCM (Figure 2e). Thus, we concluded that lung resident AT2 cells respond to secreted factors from highly metastatic breast tumors by increasing the expression of lipid production and pulmonary surfactant release genes, which is associated with increased palmitate release.

### High fat diet increases the fraction of AT2 cells

Since HFD increased palmitate availability in lungs, we examined whether AT2 cells were also involved in this effect. Therefore, we performed scRNA-seq of lungs from mice fed a HFD versus CD. In lungs of HFD mice, we observed a slight to moderate upregulation of lipid release and pulmonary surfactant genes in AT2 cells (Extended Data Figure 2b). However, the most striking observation was an increase in the total fraction of AT2 cells in HFD compared to CD mice (Extended Data Figure 2c), which is consistent with a previously report ^17^. Thus, we concluded that the mechanisms by which HFD and tumor secreted factors alter lipid availability in future organs of metastasis differ but that, at least in the lung, AT2 cells may be involved in both processes.

### Palmitate increases in metastases and feeds spheroid growth

**Extended Data Figure 3:**
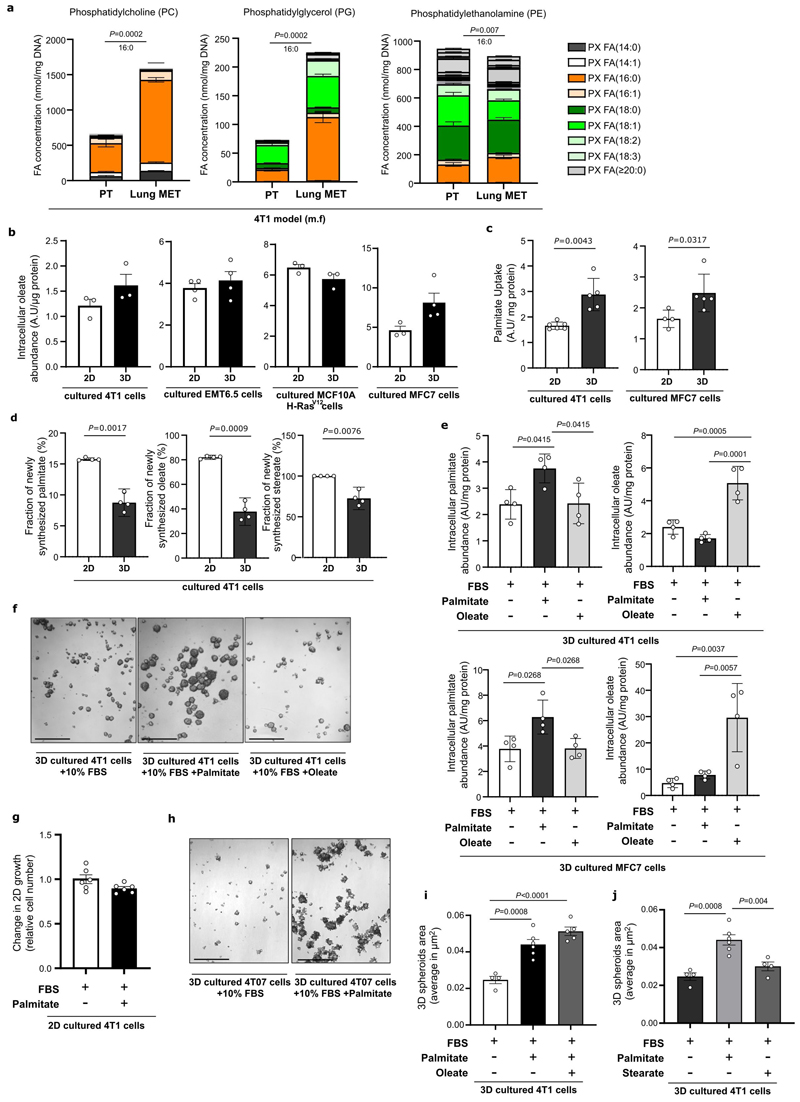
Lung metastases show an increase in lipid species enriched in palmitoyl acyl chains typically found in the pulmonary surfactant. a. Fatty acid composition of the lipid classes phosphatidylcholine, phosphatidylglycerol and phosphatidylethanolamine in breast primary tumor and lung metastasis tissues from BALB/c mice orthotopically injected with 4T1 breast cancer cells. Data are presented as mean ± SEM (n=6). Unpaired nonparametric two-tailed Mann–Whitney U-tests. b. Intracellular oleate abundance from mouse (4T1, EMT6.5) and human (MCF10A H-RasV12, MCF7) breast cancer cells cultured on soft-agar (3D) or attached (2D) conditions. Data are presented as mean ± SEM (n≥3). c. Palmitate uptake measured by 13C16-Palmitate intracellular incorporation in 3D spheroids and 2D cultured breast cancer cells after 5 days of incubation with BSA-conjugated 13C16-Palmitic acid. Data are presented as mean ± SEM (n≥4). Unpaired two-tailed t-tests with Welch correction. d. Fraction on newly synthesized fatty acids estimated by fatty acid Isotopomer Spectral Analysis (ISA) based on mass isotopomer distribution (MID) of 13C6-glucose incorporation in 3D spheroids and 2D cultured breast cancer cells in the presence of extra palmitate (75μM) for 5 days. Data are presented as mean ± SEM (n=4). Unpaired two-tailed t-tests with Welch correction. e. Intracellular palmitate and oleate abundance from 3D spheroids mouse (4T1) and human (MCF7) breast cancer cells. Data are presented as mean ± SEM (n=4). One-way ANOVA with Holm-Sidak’s multiple comparison test. f. Representative pictures of 4T1 spheroids cultured in 10%FBS, or 10%FBS in the presence of palmitate (75 μM) or oleate (116 μM). Scale bar = 0.5 μm. A representative of n=3 experiments is shown. g. Relative proliferation of 2D cultured 4T1 cells (with or without extra palmitate) normalized to condition without extra palmitate. Data are presented as mean ± SEM (n=6). h. Representative pictures of 4T07 spheroids cultured in 10%FBS with or without extra palmitate (75 μM) Scale bar = 0.5 μm. A representative of n=3 experiments is shown. i-j. 3D spheroids growth of 4T1 cells upon palmitate + oleate supplementation (i) and stearate (j) supplementation represented by the average spheroids area of >100 spheroids. Data are presented as mean ± SEM (n ≥4). One-way ANOVA with Holm-Sidak’s multiple comparison test.

**Figure 3:**
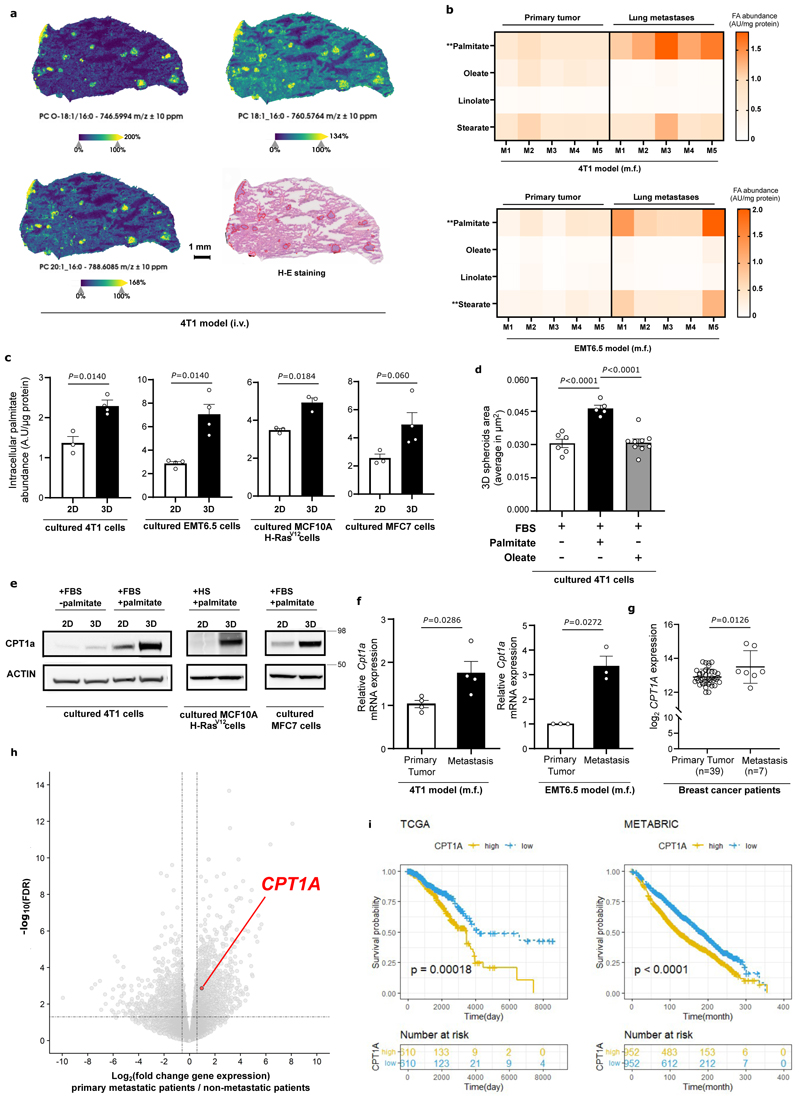
Intracellular palmitate levels as well as CPT1a expression are increased in breast cancer spheroids and lung metastasis. **a.** MALDI-MSI ion images (50 μm pixel) of lipids PC O-18:1/16:0, PC 20:1_16:0 and PC18:1_16:0 within the lung tissue. Metastases are identified by optical image of the lung tissue section following by H&E staining and are denoted by red line based on bisecting k-means segmentation map. **b.** Fatty acid abundance in 4T1 and EMT6.5 primary tumor tissues and the matching lung metastases. Data represent normalized metabolite ion counts (n=5). Unpaired nonparametric two-tailed Mann–Whitney U-tests. Asterisks represent statistical significance (**p≤ 0.01). **c.** Intracellular palmitate abundance from mouse (4T1, EMT6.5) and human (MCF10A H-Ras^V12^, MCF7) breast cancer cells cultured on soft-agar (3D) or attached (2D) conditions. Data are presented as mean ± SEM (n≥3). Unpaired two-tailed t-tests with Welch correction. **d.** 3D spheroids growth upon palmitate or oleate supplementation represented by the average of spheroids area of >100 spheroids. Data are presented as mean ± SEM (n ≥5). One-way ANOVA with Holm-Sidak’s multiple comparison test. **e.** CPT1a expression in breast cancer cells growing in 2D monolayer or 3D spheroid with or without additional palmitate. A representative image of n=3 experiments is shown. **f.** Relative *Cpt1a* gene expression in 4T1 and EMT6.5 (m.f.) breast-derived lung metastases normalized to *Cpt1a* gene expression of their breast primary tumors. Data are presented as mean ± SEM (n ≥3). Unpaired nonparametric two-tailed Mann–Whitney U-tests. **g.** *CPT1A* gene expression in breast primary tumors compared to metastatic tissues (GEO accession number GSE2109 (HS-00002(33)). Data are presented as mean ± SD (n ≥7). Unpaired two-tailed t-tests with Welch correction. **h.** Differently expressed genes in primary tumors of metastatic patients (metastasis already present at diagnosis) compared to non-metastatic patients (no metastasis during at least 7 years of followup). *CPT1A* transcript is identified as upregulated (considering an FDR-adjusted p-value threshold of < 0.05) and is colored in red. Multiple testing correction with false discovery rate (FDR) estimation. **i.** Kaplan–Meier survival for breast cancer patients with high or low levels of *CPT1a* gene expression. Comparison of survival curves was done using Mantel-COX test and Gehan-Breslow-Wilcoxon test (n=369).

Next, we asked whether the priming of palmitate in pre-metastatic lungs is reflected in metastases. Spatial mass spectrometry imaging showed that 4T1 lung metastases were enriched in palmitate-containing lipids compared to adjacent non-cancerous tissue (Figure 3a, Extended Data Figure 10). Moreover, palmitate, but not oleate or linoleate, abundance increased in 4T1 and EMT6.5 lung metastases compared to the primary tumor tissues (Figure 3b). We hypothesized that this enrichment in palmitate was linked to lipid species secreted by AT2 cells. The lipidomics analysis of lung metastases compared to primary tumors showed an enrichment in palmitoyl acyl chains of several phospholipids (PL) such as phosphatidylcholine (PC), phosphatidylglycerol (PG) and phosphatidylethanolamine (PE), which are species of the pulmonary surfactant ^18^ (Extended Data Figure 3a). Thus, these data suggest that breast cancer-derived lung metastases may take up palmitate-containing lipids released by AT2 cells.

Using a tumor spheroid assay ^19,20^ we found that 3D cultured breast cancer cells, like metastases, exhibited a palmitate, but not oleate, enrichment compared to the same breast cancer cells cultured in 2D (Figure 3c, Extended Data Figure 3b). In line, 3D cultured breast cancer cells displayed an increased fatty acid uptake and reduced *de novo* synthesis compared to 2D cultured cells (Extended Data Figure 3c-d). Subsequently, we supplemented conjugated palmitate (75 μM) and, as a negative control, oleate (116 μM) to 3D cultured breast cancer cells on top of 10% FBS resulting in a similar final concentration of 130 μM and in a selective increase of each fatty acid (Extended Data Figure 3e). Strikingly, the addition of extra palmitate, but not oleate, stimulated 3D but not 2D growth of 4T1 cells (Figure 3d, Extended Data Figure 3f-g). In accordance with our *in vivo* data, also 3D spheroid growth of low metastatic 4T07 cancer cells increased with extra palmitate (Extended Data Figure 3h). Since oleate can buffer the effects of palmitate in cancer cells ^21–23^, we also added investigated the combination of both fatty acids. Unexpectedly, the growth increase observed with extra palmitate was also present when palmitate was combined with oleate (Extended Data Figure 3i), while stearate did not phenocopy palmitate (Extended Data Figure 3j), suggesting that only palmitate promotes spheroid growth of 4T1 breast cancer cells. Taken together, these data show that breast cancer-derived lung metastases are enriched in palmitate and that increasing palmitate availability further promotes spheroid growth.

### CPT1a expression is upregulated in breast cancer metastases

**Extended Data Figure 4:**
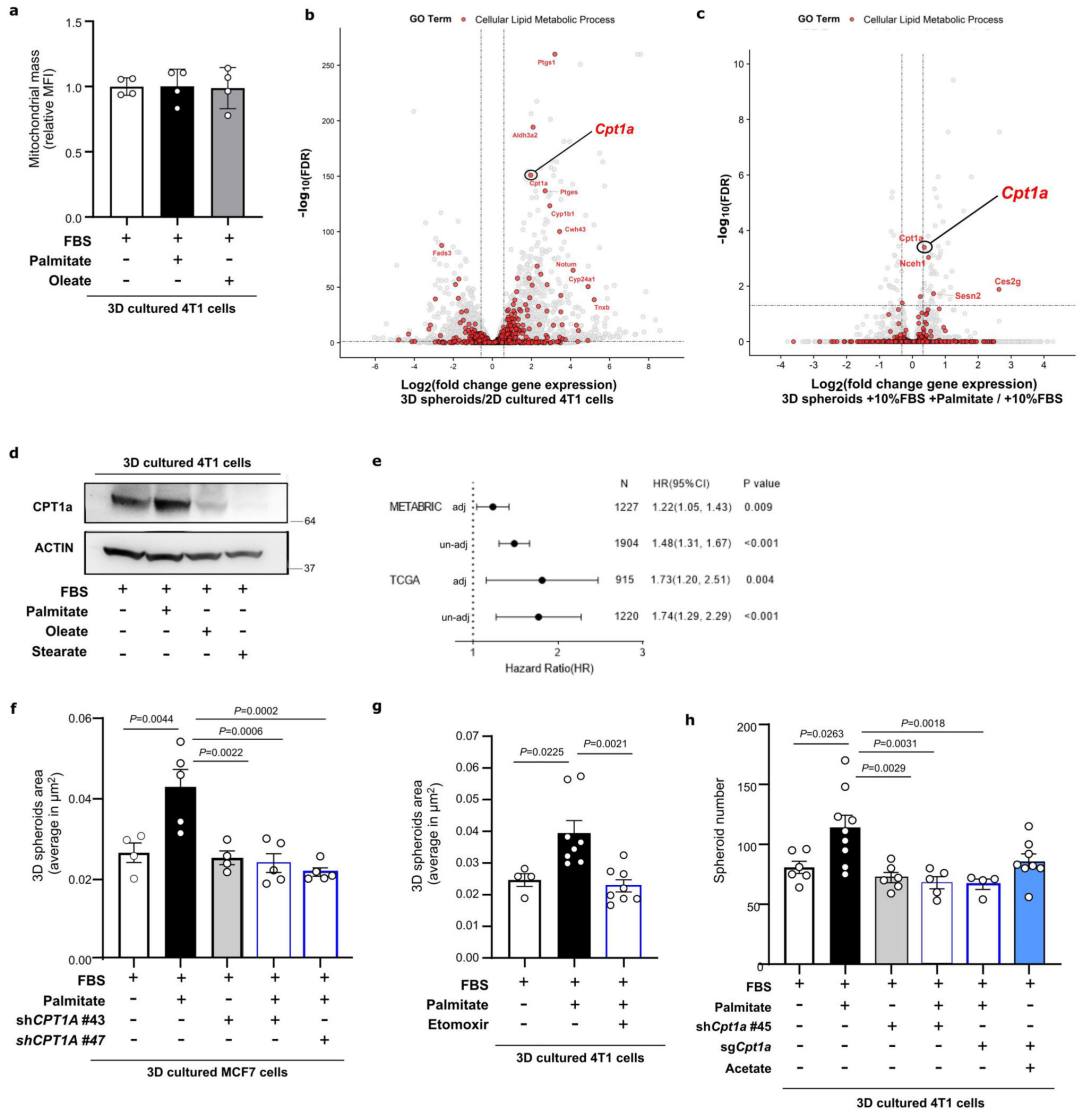
CPT1a expression is upregulated in metastasis and is associated with poor prognosis in breast cancer patients. a. Mitochondrial mass represented by mean relative fluorescence (MFI) of MitoTracker in 3D spheroids 4T1 cells growing in the absence or presence of palmitate or oleate for 5 days. MFI is shown relative to the level of MitoTracker fluorescence of 10% FBS condition. Data are presented as mean ± SD (n=4). b-c. Differentially expressed genes in (b) 4T1 cells cultured in 3D spheroids or 2D monolayer in the presence of extra palmitate, or (c) in 3D spheroid 4T1 in the presence or absence of extra palmitate (10% FBS + palmitate or 10% FBS). Calculated differences in gene expression are presented by plotting the negative log10 of false discovery rate (Y-axis) against the log2 fold change of gene expression (X-axis). Each dot represents an individual gene. In red, genes belong to the GEO term lipid metabolic process (GO:0006629). The highest-ranking (top 10) overexpressed genes or genes above the established cutoff are annotated. d. CPT1a expression in 3D spheroid 4T1 cells growing for 5 days in the presence of additional palmitate, oleate and stearate. A representative image of n=3 experiments is shown. e. Forest plot depicting the hazard ratio (HR) (x-axis) and corresponding 95% confidence intervals (denoted by error bars) for overall survival of patients with primary breast cancer according to CPT1A expression from two different cohorts: TCGA (n=1,221) and METABRIC (n= 1,904). Cox proportional hazards models were applied, controlling for age, tumor stage (cTNM-staging system), and tumor subtype in both datasets. Panel complementary to Figure 3i. f. 3D spheroids growth of MCF7 cells upon CPT1a knockdown compared to scrambled control upon palmitate supplementation (75 μM) represented by the average spheroids area of >100 spheroids. Data are presented as mean ± SEM (n≥4). One-way ANOVA with Tukey’s multiple comparison test. g. 3D spheroids growth (5 days) of 4T1 cells upon CPT1a inhibition using etomoxir (50 μM) in the presence of extra palmitate (75 μM) represented by the average spheroids area of >100 spheroids. Data are presented as mean ± SEM (n≥4). One-way ANOVA with Tukey’s multiple comparison test. h. 3D spheroids number per well of 4T1 cells upon palmitate supplementation (75 μM), CPT1a genetic inhibition performed by shRNA (shCpt1a) and CRISPR (sgCpt1a), and upon metabolic rescue by acetate (5mM). Data are presented as mean ± SEM (n≥4). One-way ANOVA with Tukey’s multiple comparison test.

Palmitate can have different fates in cells. We analyzed lipid processing pathways using RNA-seq comparing 3D versus 2D cultured 4T1 cells with additional palmitate, and 3D cultures with and without it. Despite mitochondrial mass being unchanged (Extended Data Figure 4a), we found that carnitine palmitoyltransferase 1a (CPT1a), which facilitates the import of long-chain fatty acid into the mitochondria, was within the highest-ranking lipid processing enzymes whose gene expression increased in 3D compared to 2D cultures and upon additional palmitate availability in 3D cultures (Extended Data Figure 4b-c). We confirmed this increase at the protein level in 4T1 as well as human MCF7 and MCF10A H-Ras^V12^ breast cancer cells (Figure 3e, Extended Data Figure 4d). Moreover, lung metastases compared to the corresponding primary 4T1 and EMT6.5 breast tumors displayed increased CPT1a expression (Figure 3f). Additionally, we found that CPT1a expression was higher in metastatic samples compared to primary tumor tissue of patients with breast cancer (Figure 3g, Expression Project for Oncology - expO, GSE2109).

Next, we performed RNA-seq on breast primary tumors of 14 patients that had metastases at diagnosis compared to 44 patients that did not have metastases at diagnosis and did not develop such within at least 6-years (CHEMOREL study at UZ Leuven, Supplementary Table 3). We observed that patients with metastatic breast cancer at diagnosis had higher *CPT1A* expression in their primary breast tumors compared to patients that did not develop metastases for at least 7 years (Figure 3h). We further analyzed gene expression data from patients with breast cancer (The Cancer Genome Atlas program (TCGA) ^24^ and METABRIC ^25^) and found that high CPT1a expression (above median) in primary breast tumors of patients was significantly associated with a decreased overall survival (Figure 3i). This association was confirmed after correcting for the common clinic-pathological variables (age, tumor stage and tumor subtype, Supplementary Table 4, HR_adj_ [95%confidence interval]: 1.73[1.20–2.51] and HR_adj_: 1.22[1.05-1.43] in TCGA and METABRIC dataset respectively, Extended Data Figure 4e). Based on these data, we concluded that CPT1a may be important for breast cancer progression in patients and that breast cancer-derived metastases may rely on CPT1a to process palmitate.

### Metastasis requires CPT1a in lean and high fat diet mice

**Extended Data Figure 5:**
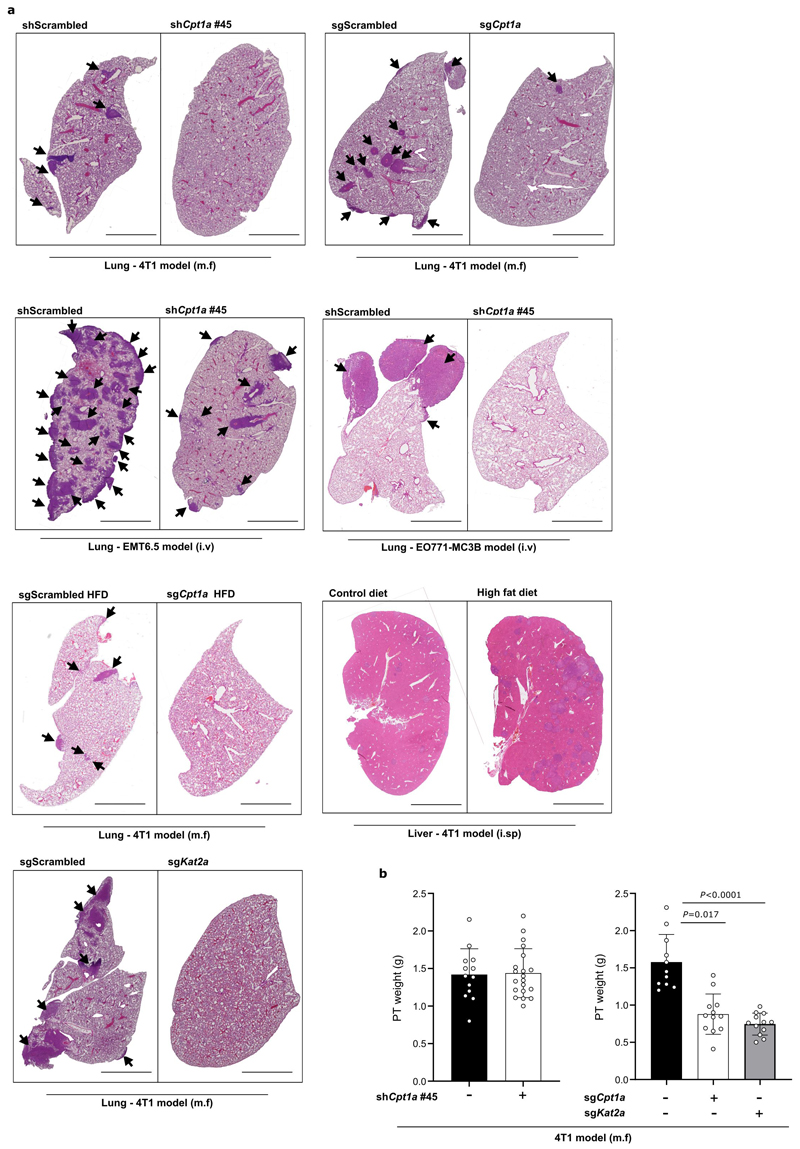
Representative lung and liver H&E staining and primary tumor weight upon CPT1a and KAT2a loss. a. Representative pictures of tissue from the lung of mice injected with 4T1 (m.f.) and EMT6.5 (i.v.) upon genetic inhibition of CPT1a compared to non-targeting sg/shRNA as a control, based on H&E staining. Arrowheads indicate metastasis tissue. Scale bars= 2 mm. b. Final primary tumor weight (grams) from individual breast tumors upon genetic inhibition of CPT1A and KAT2A in the 4T1 model (m.f.). Data are presented as mean ± SEM (n ≥11). One-way ANOVA with Dunnett’s multiple comparison test.

**Extended Data Figure 6:**
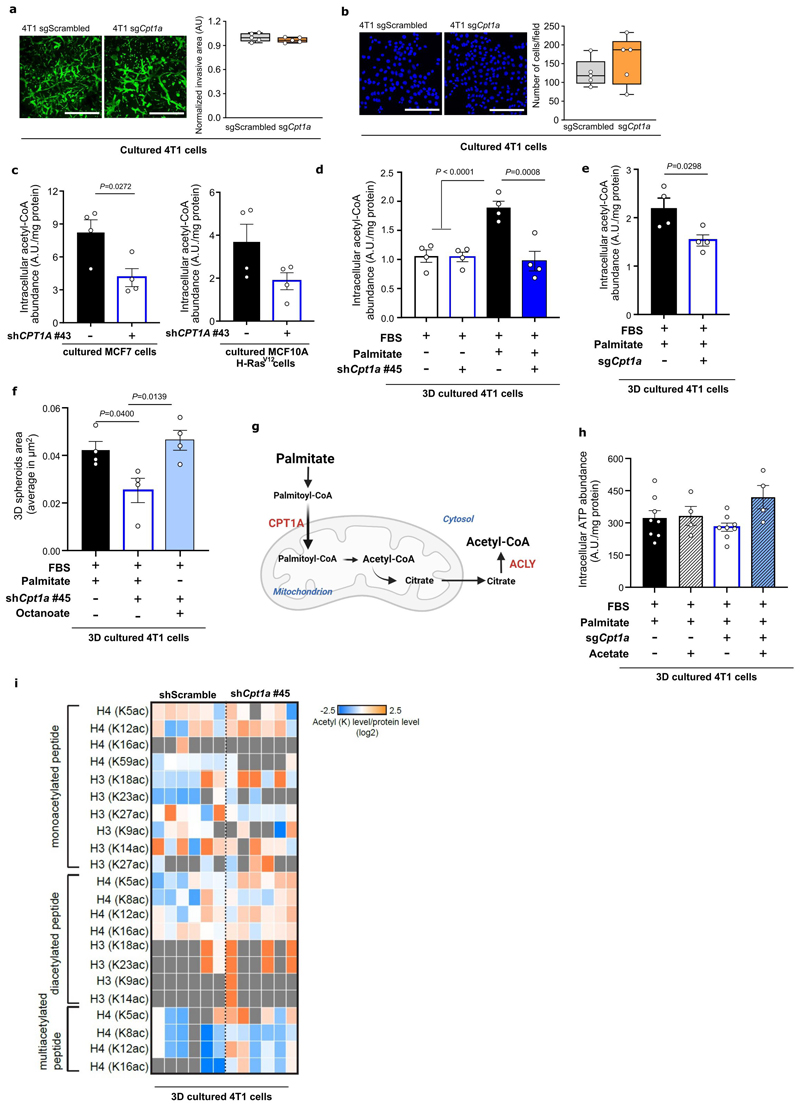
Breast cancer spheroids in the presence of additional palmitate rely on CPT1a for acetyl-CoA production and for sustaining palmitate-induced 3D growth. a. Invasive ability in a 3D matrix of 4T1 cells upon CPT1a knockout (sgCpt1a) compared to control (Scrambled) cells. Invasion was assessed by measuring the invasive area of cancer cells stained with calcein green. Representative images are depicted in the left panel (scale bar 500 μm), quantification in the right panel. Each dot represents a different, randomly selected microscopy field (n=5). b. Migratory ability of 4T1 upon CPT1a knockout (sgCpt1a) compared to control (Scrambled) cells. Migration was assessed by analyzing the total cells migrated through transwells coated with endothelial cells. Blue, DAPI nuclear staining. Representative images are depicted in the left panel (scale bar 500 μm), quantification in the right panel. Each dot represents a different, randomly selected microscopy field (n=5). c. Relatives changes in acetyl-CoA abundance in human MCF10A H-RasV12 and MCF7 breast cancer spheroids transduced with a lentiviral vector with shRNA against CPT1A (knockdown) compared to scrambled control sequences in the presence of extra palmitate. Data are presented as mean ± SEM (n=4). Unpaired two-tailed t-tests with Welch correction. d-e. Relatives changes in acetyl-CoA abundance in mouse 4T1 breast cancer spheroids transduced with a lentiviral vector with RNA against Cpt1a (c, knockdown and d, knockout) compared to non-targeting sh/sgRNA control in the presence or absence of extra palmitate. Data are presented as mean ± SEM (n=4). One-way ANOVA with Dunnett’s multiple comparison test or two-tailed unpaired student’s T-test. f. 3D spheroids growth (5 days) of 4T1 cells upon palmitate supplementation (75 μM), CPT1a genetic inhibition (shCpt1a) and metabolic rescue with octanoate (130 μM) compared to non-targeting shRNA control, represented by the average of spheroids area of >100 spheroids. Data are presented as mean ± SEM (n=4). One-way ANOVA with Dunnett’s multiple comparison test. g. Schematic representation of the palmitate flux into the mitochondria via CPT1A and the ACLY-dependent export of the mitochondrial acetyl-CoA pool to the cytosol via citrate. h. Intracellular levels of ATP in CPT1a knockout and control 4T1 3D spheroids cultured for 5 days in medium containing extra palmitate (75 μM) or acetate as metabolic rescue (5 mM). Data are presented as mean ± SEM (n ≥4). i. Heatmap display of the log2 transformed ratios obtained for the indicated histone acetylation for CPT1a knockdown and control 4T1 3D spheroids cultured for 5 days in medium containing extra palmitate (75 μM). Relative abundances ratios, light/SILAC heavy, were obtained with the SILAC (Stable Isotope Labeling with Amino acids in Cell culture) internal standard strategy.

**Figure 4:**
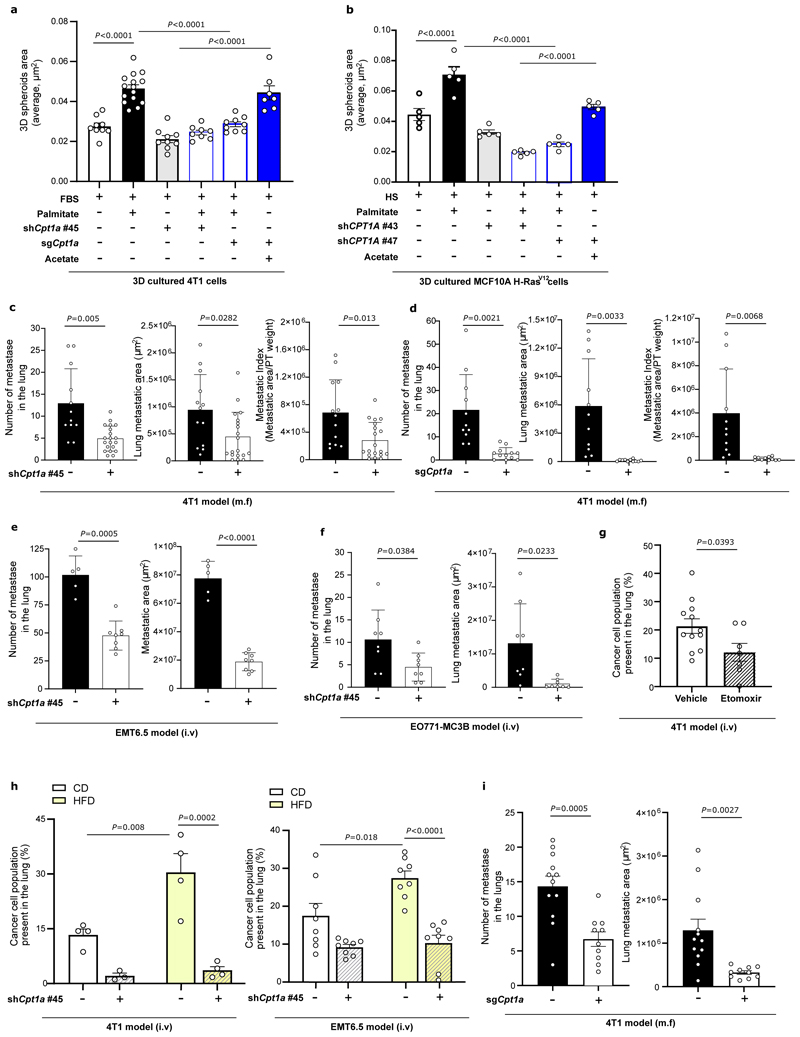
Silencing CPT1a counteracts palmitate-induced spheroid growth and inhibits metastasis formation in lean and obese mice. **a-b.** 3D spheroids growth of 4T1 and MCF10A H-RasV^12^ cells upon palmitate supplementation (75 μM), CPT1a genetic inhibition performed by shRNA (sh*Cpt1a* and sh*CPT1A*) and CRISPR (sg*Cpt1a*) compared to cells infected with non-targeted sh/sgRNA as a control, and upon metabolic rescue by acetate (5mM), represented by the average of spheroids area of >100 spheroids. Data are presented as mean ± SEM(n ≥4). One-way ANOVA with Tukey’s multiple comparison test. **c-d**. Metastatic burden in lung of mice injected with 4T1 in the mammary fat pad (m.f.) upon genetic (**c**) knockdown or (**d**) knockout of *Cpt1a* compared to cells infected with non-targeted sgRNA as a control, analyzed by H&E staining. Data are presented as mean ± SEM (n≥11). Data for sg-control and *sgCpt1a* are also shown in Figure 7f. Unpaired two-tailed t-tests with Welch correction. Representative H&E staining images are shown in Extended Data Figure 5a. **e-f.** Total area and number of metastases in lung of mice after 12-14 days of intravenous (i.v.) injections with EMT6.5 or EO771-MC3B cancer cells previously transduced with a lentiviral vector with shRNA against *Cpt1a* or scramble sequence as a control, and analyzed by H&E staining. Data are presented as mean ± SEM (n≥5).. Unpaired two-tailed t-tests with Welch correction. Representative H&E staining images are shown in Extended Data Figure 6a. **g.** Percentage of breast cancer cells present in lung of mice after 14 days of intravenous (i.v.) injections with CD90.1-4T1 cells. Mice were treated intraperitoneally with the CPT1a inhibitor etomoxir (40 mg/kg) or vehicle (water) daily starting after 4 days of cancer cell injections. Data are presented as mean ± SEM (n≥7). Unpaired two-tailed t-tests with Welch correction. **h.** Percentage of cancer cells (mean ± SEM) present in lung of mice after 12 days of intravenous (i.v.) injections with CD90.1-labeled 4T1 or EMT6.5 cancer cells, and expressingshRNA against *Cpt1a* or scramble sequence as a control. Before injections, mice were maintained during 16 weeks in CD and HFD (n ≥4). Data for 4T1 sh-control in CD and HFD are also shown in Figure 1d. Two-way ANOVA with Tukey’s multiple comparison test. **i**. Metastatic burden in lung of mice injected with 4T1 in the mammary fat pad (m.f.) upon *Cpt1a* knockout compared to cells infected with non-targeted sgRNA as a control, analyzed by H&E staining. Data are presented as mean ± SEM (n≥5). Before injections, mice were maintained during 16 weeks in CD and HFD. Unpaired two-tailed t-tests with Welch correction. Representative H&E staining images are shown in Extended Data Figure 5a.

We then assessed how CPT1a inhibition affects palmitate promoted spheroid growth. We found that genetic or pharmacologic inhibition of CPT1a decreased spheroid size and number to the level observed without additional palmitate in 4T1, MCF7 and MCF10A H-Ras^V12^ cells (Figure 4a-b, Extended Data Figure 4f-h, Extended Data Figure 9). Based on this *in vitro* finding, we hypothesized that blocking CPT1a is sufficient to reduce lung metastasis formation. We assessed experimental and spontaneous lung metastasis formation by injecting control and CPT1a knockout/down cells (Extended Data Figure 9) intravenously (4T1, EMT6.5 and EO771-MC3B) or into the mammary fat pad (4T1) of mice, respectively. We observed that metastatic burden was highly reduced in the absence of CPT1a expression compared to control (Figure 4c-d, Extended Data Figure 5a), while primary tumor growth showed no changes (upon CPT1a knockdown) and a small reduction (upon CPT1a knockout) compared to control (Extended Data Figure 5b). Similarly, also intravenous injections of CPT1a silenced 4T1, EMT6.5 and EO771-MC3B breast cancer cells reduced metastasis formation compared to control (Figure 4e-h) showing that this effect was not dependent on the dissemination of cancer cells from the primary tumor. In line, we did not observe differences in the invasion and migration capacity of 4T1 cells upon CPT1a inhibition *in vitro* (Extended Data Figure 6a-b). Furthermore, inhibiting CPT1a by treating mice after initial metastatic colonization (4 days) with etomoxir (40 mg/Kg i.p. daily) also reduced metastasis formation (Figure 4g). Thus, we concluded that blocking CPT1a is sufficient to impair the growth of breast cancer cells in the lung environment.

**Figure 5:**
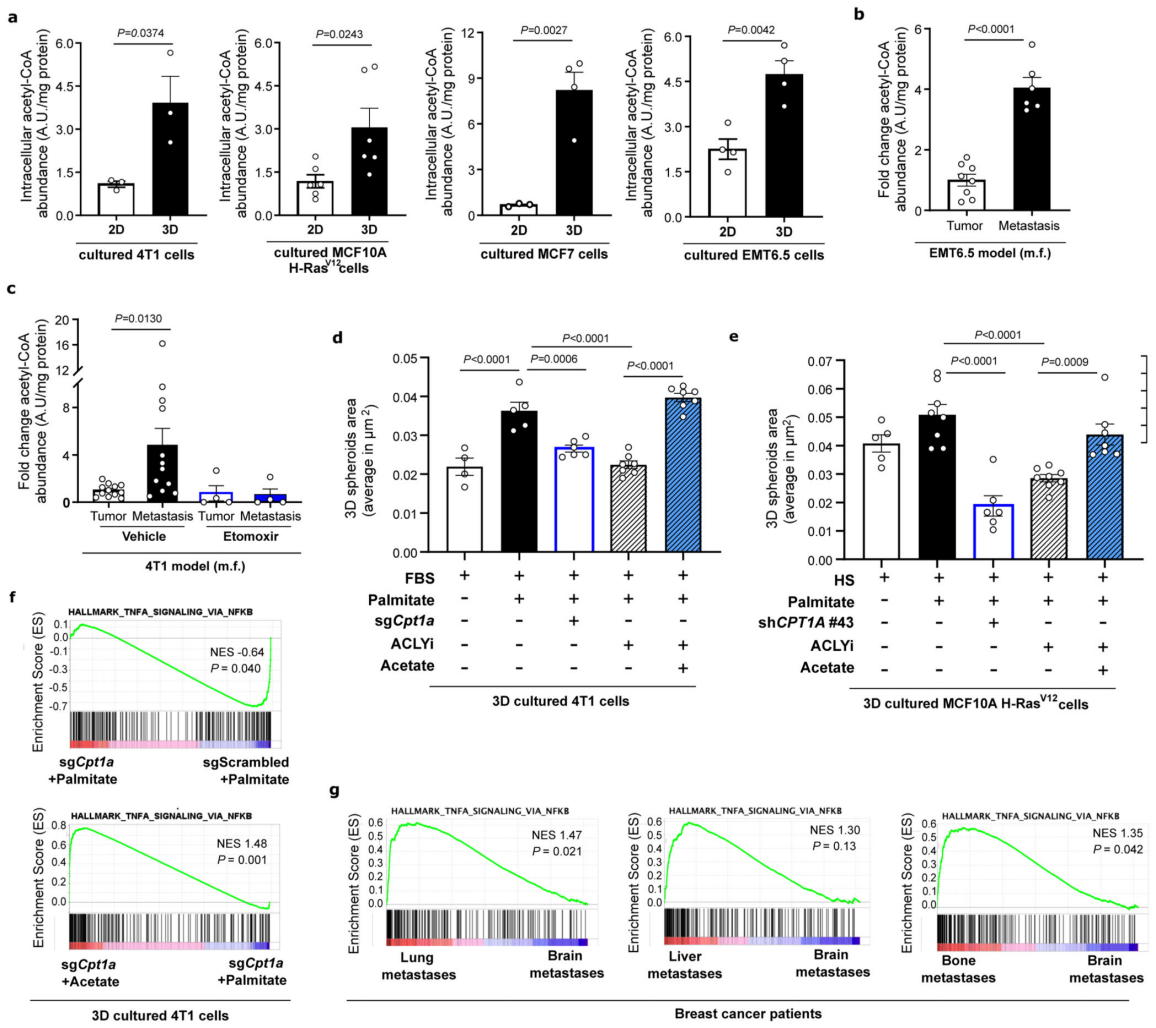
CPT1a activity sustains acetyl-CoA levels in spheroids and lung metastases. **a.** Intracellular levels of acetyl-CoA in breast cancer cells incubated in 2D monolayer and 3D spheroids cultures for 5 days in medium containing extra palmitate. Data are presented as mean ± SEM (n=4).Unpaired two-tailed t-tests with Welch correction. **b.** Relatives changes in acetyl-CoA abundance in EMT6.5 (m.f.) breast primary tumors and lung metastases. Data are shown as fold changes compared with the acetyl-CoA abundance in the primary tumors. Data are presented as mean ± SEM (n=4). Unpaired two-tailed t-tests with Welch correction. **c.** Relatives changes in acetyl-CoA abundance in 4T1 (m.f.) breast primary tumors and lung metastases upon acute inhibition of CPT1A using the inhibitor etomoxir (40 mg/kg i.p.) or vehicle (water). Data are shown as fold changes compared with the acetyl-CoA abundance in the primary tumors of the group of mice treated with the vehicle. Data are presented as mean ± SEM and points represented as zero were below the detection limit (n≥4).. One-way ANOVA with Dunnett’s multiple comparison test. **d-e.** 3D spheroids growth upon genetic inhibition of either *Cpt1a*/*CPT1A* compared to cells infected with scramble as a control together with pharmacologic ALCY inhibition using BMS-303141 (20 μM, 5 days) in **(d)** 4T1 and **(e)** MCF10A H-Ras^V12^ cells with or without extra palmitate and in the presence of the acetate as metabolic rescue (5 mM, 5 days). 3D spheroid growth is represented by the average spheroids area of >100 spheroids. Data are presented as mean ± SEM (n ≥4). One-way ANOVA with Tukey’s multiple comparison test. **f.** GSEA enrichment plots comparing gene expression profiles in 4T1 3D spheroids transduced with a lentiviral vector containing sg*Cpt1a* or sgControl (top panel) and sg*Cpt1a* 4T1 3D spheroids cultured with or without acetate (bottom panel). NES, normalized enrichment score; the P value indicates the significance of the enrichment score (permutation test). **g.** GSEA enrichment plots comparing gene expression profiles of HALLMARK_TNFA_SIGNALING_VIA_NFKB signature from the Molecular Signature Database (MsigDB) in breast cancer metastases at different organ sites from patients (GSE14018). NES, normalized enrichment score; the P value indicates the significance of the enrichment score (unpaired one-tailed t-tests).

Next, we hypothesized that CPT1a also mediates the increased growth capacity of metastases in HFD-fed mice. We injected intravenously 4T1 and EMT6.5 control and CPT1a silenced cells expressing the congenic marker CD90.1 into mice on CD or HFD. Assessing metastatic burden after 12 days, we found an increased fraction of CD90.1 positive cells in the lung of HFD compared to CD mice (Figure 4h). Silencing CPT1a was sufficient to prevent this increase induced by HFD (Figure 5h). This striking dependence on CPT1a was also observed in spontaneous metastases arising from mammary fad pad injected HFD-fed mice (Figure 4i, Extended Data Figure 5). These results show that lung metastasis formation depends on CPT1a in lean and HFD mice.

### CPT1a activity sustains acetyl-CoA levels

Next, we investigated the mechanism by which palmitate supports metastasizing cells. CPT1a facilitates the transport of long-chain fatty acyl-CoAs into the mitochondria, and one of the main fates for those is the subsequent oxidation to acetyl-CoA in the β-oxidation pathway. Therefore, we measured acetyl-CoA abundance in 3D spheroids and metastases tissue. We found that acetyl-CoA abundance was higher in breast cancer cells supplemented with extra palmitate in 3D compared to 2D culture (Figure 5a) and in lung metastases compared to primary 4T1 and EMT6.5 breast tumors (Figure 5b-c). Accordingly, acetyl-CoA abundance decreased in 3D cultured mouse (4T1 and EMT6.5) and human (MCF7 and MCF10A H-Ras^V12^) breast cancer cells upon CPT1a silencing (Extended Data Figure 6c-e) as well as in 4T1 lung metastases when the mice were treated with etomoxir (40 mg/kg, i.p. injection once a day starting 72 hours before euthanasia ^26^) (Figure 5c). Without extra palmitate, CPT1a silencing did not decrease acetyl-CoA abundance in 3D cultured 4T1 breast cancer cells (Extended Data Figure 6d). Moreover, the 3D growth defect observed upon CPT1a inhibition was rescued by acetate (5mM; Figure 4a-b) and octanoate (130 μM, Extended Data Figure 6f), which are CPT1a independent sources of acetyl-CoA. These findings show that breast cancer spheroids in the presence of additional palmitate and breast cancer-derived lung metastases rely on CPT1a for acetyl-CoA production.

### Cpt1a-mediated palmitate oxidation increases NF-κB signaling

**Extended Data Figure 7:**
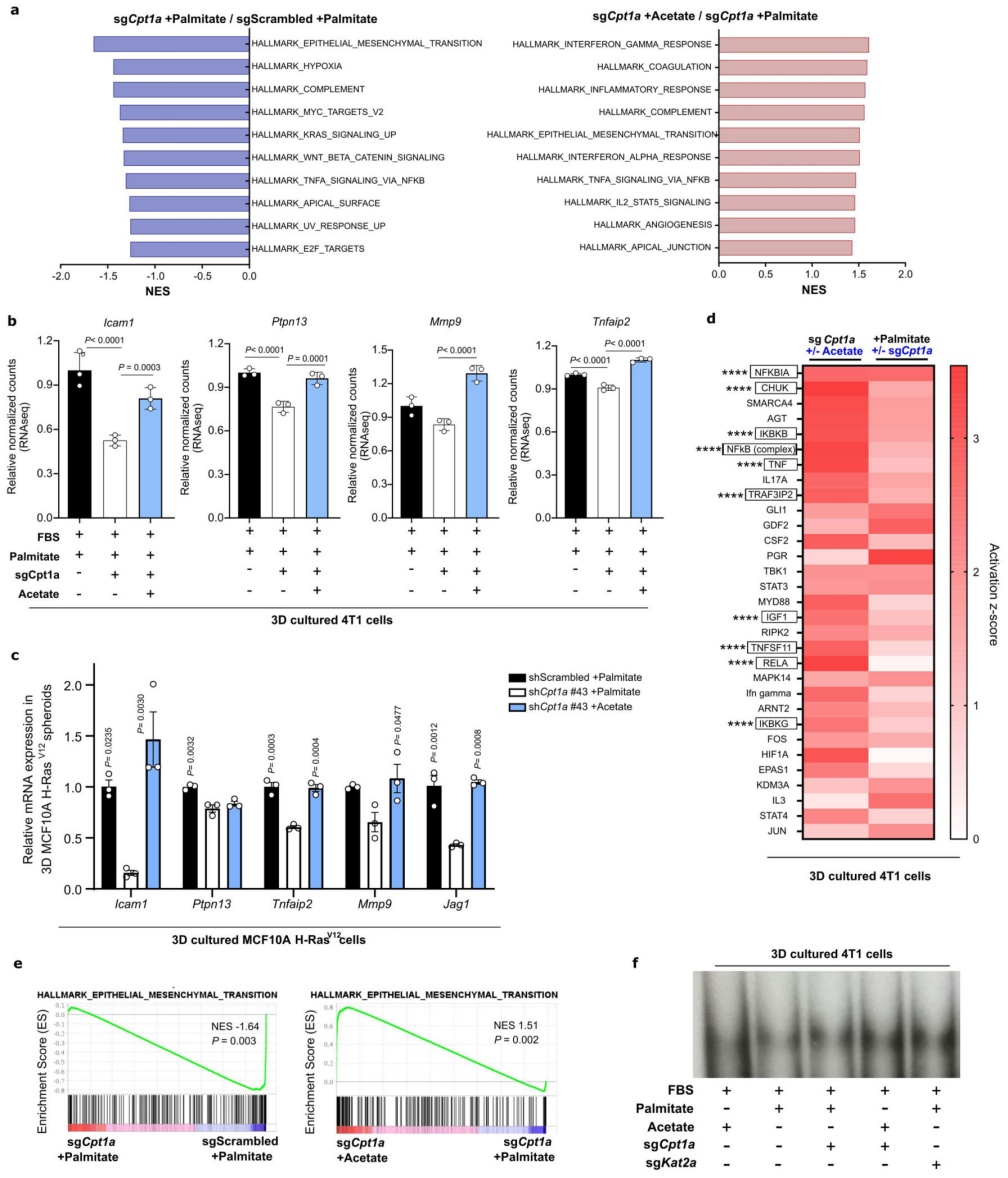
CPT1a deletion reduces NF-κB signaling pathway but does not affect p65 DNA binding. a. Top 10 enriched pathways (p<0.05) obtained from gene set enrichment analysis (GSEA) of 4T1 spheroids upon CPT1a inhibition (sgCPT1a), in the presence of the extra palmitate (75 μM) or acetate as metabolic rescue (5 mM) using the Hallmark gene set from Molecular Signatures Database (MSigDB). NES, normalized enrichment score. b. Relative expression of genes implicated in invasion and metastasis, and that are known to be regulated via NF-κB activation in 4T1 spheroids. Fold change is calculated from normalized raw counts (RNA sequencing) of CPT1a knockout and non-targeting sgRNA control 4T1 3D spheroids cultured for 5 days in medium containing extra palmitate (75 μM) or acetate (5 mM). Data are presented as mean ± SD (n=3). Multiple testing correction with false discovery rate (FDR) estimation. c. Relative expression of genes implicated in invasion and metastasis, and that are known to be regulated via NF-κB activation in MCF10A H-RasV12 spheroids. Fold changes are calculated for CPT1a knockdown and non-targeting sgRNA control MCF10A H-RasV12 3D spheroids cultured for 5 days in medium containing extra palmitate (75 μM) or acetate (5 mM) and are normalized to gene expression in control cells. Data are presented as mean ± SEM (n=3). One-way ANOVA with Dunnett’s multiple comparison test. d. Upstream regulator analysis performed using Ingenuity Pathway Analysis using the differential gene expression of CPT1A knockout (sgCpt1a) 4T1 3D spheroids in the presence or absence of acetate (metabolic rescue, 5 mM) as input. Activation score of the top 30 upstream regulators (left column) was compared to those predicted for the differential gene expression of CPT1A knockout versus control conditions (in the presence of extra palmitate). Genes related to the activation of the NF-κB pathway are framed. Asterisks represent the overlap p-value calculated using one-sided Fisher’s Exact Test (****p < 0.0001). e. GSEA enrichment plots comparing the gene expression profiles in 4T1 3D spheroids transduced with a lentiviral vector containing sgCpt1a or sgScrambled as a control (left panel) and sgCpt1a 4T1 3D spheroids cultured with or without acetate (right panel). NES, normalized enrichment score; the P value indicates the significance of the enrichment score (permutation test). f. Total p65 binding to DNA measured by electrophoretic mobility shift assay (EMSA). Arrow indicates the position of the NF-κB containing complex. A representative of n=3 experiments is shown.

Next, we addressed how CPT1a-derived acetyl-CoA supports metastasizing cells. If acetyl-CoA is required in the cytosol, inhibiting ATP citrate lyase (ACLY) is expected to phenocopy CPT1a inhibition (Extended Data Figure 6g). Indeed, we found that inhibiting ACLY with BMS-303141 (20μM for 5 days, ACLYi) decreased 3D growth to a similar extent as CPT1a inhibition and that acetate rescued this growth defect (Figure 5d-e). Moreover, the level of ATP, a product of mitochondrial acetyl-CoA metabolism, was not significantly altered upon CPT1a deletion and acetate supplementation (Extended Data Figure 6h). Thus, we investigated possible fates of acetyl-CoA beyond mitochondria metabolism. One such possibility is histone acetylation ^27^. Yet, we found no prominent changes in histone acetylation in 3D cultured 4T1 cells upon CPT1a silencing using proteomics (Extended Data Figure 6i). Therefore, we further investigated non-histone protein acetylation, which may be connected to gene expression regulation ^28^. We performed RNA-seq and consecutive gene set enrichment analysis (GSEA) in 4T1 spheroids upon CPT1a deletion and acetate rescue. Among the highest-ranking gene sets (Extended Data Figure 7a) was a signature for NF-κB signaling that decreased upon CPT1a deletion and was rescued with acetate (Figure 5f, Extended Data Figure 7b). These gene expression changes were confirmed in an additional breast cancer cell line (Extended Data Figure 7c). Conversely, we analyzed all CPT1a-dependent and acetate rescued gene expression changes to predict upstream regulators using Ingenuity Pathway Analysis ^29^. Remarkably, several upstream regulators of the NF-κB signaling pathway scored within the top 30 predicted regulators (Extended Data Figure 7d). Thus, this shows that NF-κB signaling is likely induced by palmitate in a CPT1a-dependent manner. NF-κB signaling can induce an epithelial-to-mesenchymal transition (EMT) ^1,30^. Accordingly, we found a reduced EMT signature upon CPT1a deletion that was rescued with acetate (Extended Data Figure 7e). Moreover, we analyzed publicly available data from metastatic breast cancer patients and found an overrepresentation of NF-κB signaling in lung, liver and bone metastases, which grow in lipid-rich environments (Figure 1a-b and ^31^) compared to brain metastases where CSF and interstitial fluid are lipid deprived environment ^32,33^ (Figure 5g). Thus, these data are consistent with the notion that NF-κB signaling is activated downstream of the CPT1a-mediated palmitate oxidation in 3D spheroids.

### CPT1a promotes acetylation of the NF-κB subunit p65

**Figure 6:**
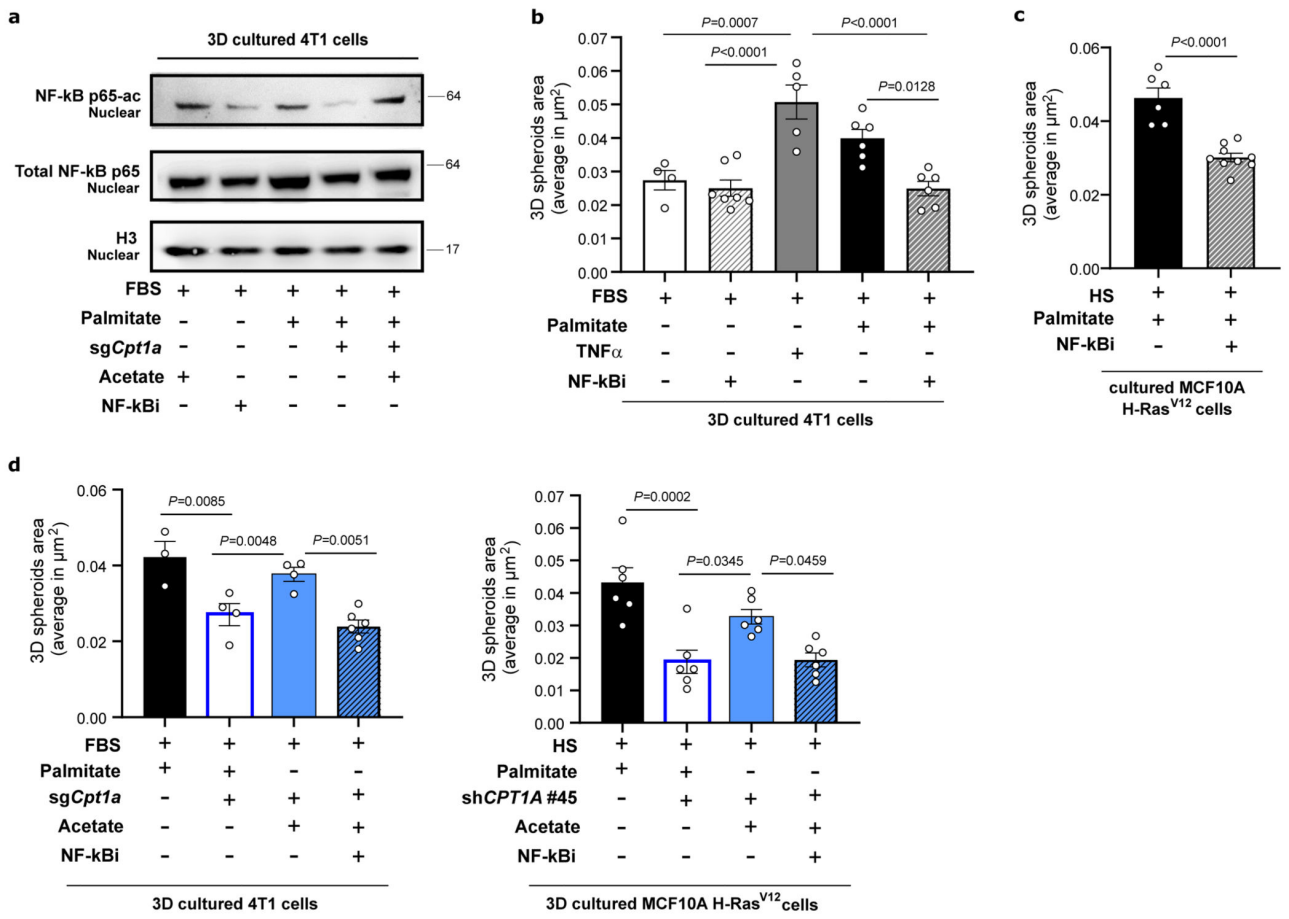
CPT1a is required for p65 acetylation and NF-κB signaling. **a.** Acetylated p65 (NF-κB p65ac) in nuclear extracts of 3D spheroids transduced with a lentiviral vector containing sg*Cpt1a* or sgcontrol and cultured for 3 days in the presence of extra palmitate (75 μM), acetate (5 mM) or the NF-κB inhibitor PDTC (0.5 μM). Histone H3 is shown as a loading control of NF-κB p65ac. A representative of n=3 experiments is shown. **b-c.** 3D growth (5 days) of (**b**) 4T1 and (**c**) MCF10A H-Ras^V12^ cells upon treatment with the NF-κB inhibitor (PDTC, 0.5 μM) in the presence of extra palmitate and 4T1 3D spheroids growth upon activation of the pathway via either supplementation of TNFa (10 ng/μL) or extra palmitate (75 μM) is shown. 3D spheroid growth is represented by the average spheroids area of >100 spheroids. One-way ANOVA with Tukey’s multiple comparison test (4T1, n≥4) and Unpaired two-tailed t-tests with Welch correction (MCF10A H-Ras^V12^, n=4). Data are presented as mean ± SEM. **d.** 3D growth (5 days) of 4T1 (left panel) and MCF10A H-Ras^V12^ (right pale) cells upon treatment with the NF-κB inhibitor to the inhibitory impact of CPT1a inhibition (sg*Cpt1a*) compared to non-targeting shRNA as a control, in the presence of the extra palmitate (75 μM) or acetate as metabolic rescue (5 mM). 3D spheroid growth is represented by the average spheroids area of >100 spheroids. Data are presented as mean ± SEM (n ≥4). One-way ANOVA with Tukey’s multiple comparison test.

Acetylation of the NF-κB family member p65 is known to dynamically regulate NF-κB activation and transcriptional activity ^34^. Thus, we asked whether p65 acetylation is regulated by CPT1a. To address this question, we determined the amount of total p65 binding to the DNA by electrophoretic mobility shift assay (EMSA) and p65 acetylated at K310 in the nucleus upon CPT1a silencing and upon acetate supplementation in 4T1 spheroids using acetylation-specific western blot analysis. Total nuclear p65 and DNA binding activity were not altered (Figure 6a, Extended Data Figure 7f), which may indicate that the genome-wide distribution of p65 is not altered. Strikingly, however we found that K310 acetylated p65 in the nucleus decreased upon CPT1a silencing and was rescued upon acetate supplementation (Figure 6a). These findings suggest an importance of p65 acetylation in mediating the transcription of distinct NF-κB targets.

Next, we modulated NF-κB signaling and assessed spheroid growth. TNFα is an activator of NF-κB, while pyrrolidine-dithiocarbamate ammonium (PDTC 0.5 μM for 5 days, NF-κBi) blocks the translocation of p65 into the nucleus reducing its transcriptional activity ^35^. Treating 4T1 and MCF10A H-Ras^V12^ cancer cells with TNFα increased spheroid growth even without extra palmitate, while NF-κBi treatment decreased spheroid growth in the presence of extra palmitate (Figure 6b-c). Accordingly, acetate did not any longer rescue spheroid growth in the presence of NF-κBi (Figure 6d). Therefore, we concluded that palmitate-derived acetyl-CoA production is required for p65 acetylation and downstream NF-κB signaling.

### KAT2a acetylates p65 in the presence of palmitate

**Extended Data Figure 8:**
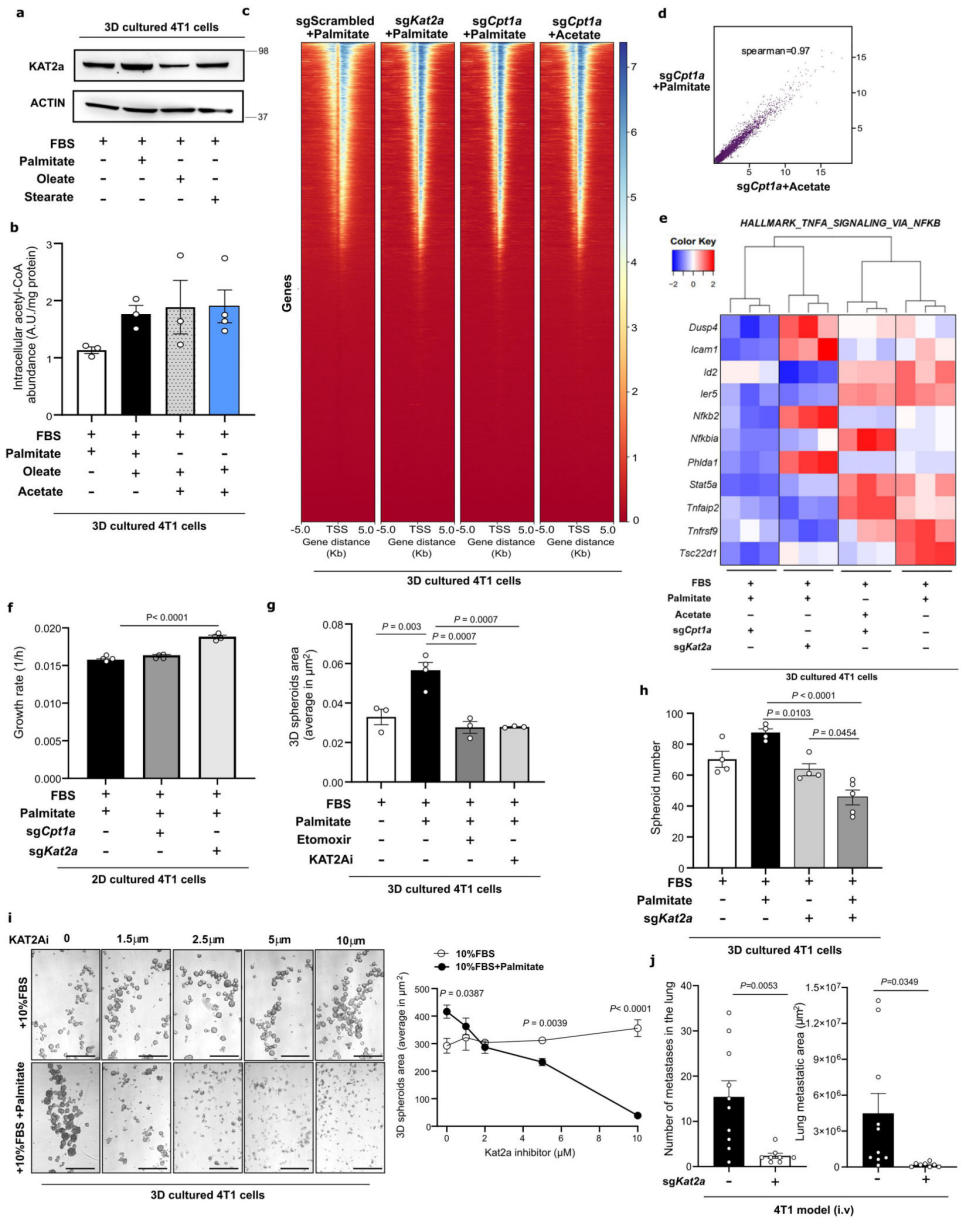
Global histone acetylation and chromatin accessibility are not consistently changed upon CPT1a and/or KAT2a loss a. KAT2a expression in 3D spheroid 4T1 cells growing for 5 days in the absence or presence of additional palmitate, oleate and stearate. A representative image of n=3 experiments is shown. b. Intracellular levels of acetyl-CoA in 4T1 cells growing in 3D for 5 days in medium containing only 10% FBS, or supplemented with palmitate (75 ?m), oleate (116 μM) or acetate (5 mM). Data are presented as mean ± SEM (n=4). c. Heatmap of the signal intensity of H3K9ac-targeted gene loci in non-targeting RNA control, CPT1a and KAT2a knockout 4T1 3D spheroids cultured for 5 days in medium containing extra palmitate (75 μM) (n=3). d. Correlation plot of H3K9 acetylation in 4T1 3D spheroids cultured in the presence of palmitate upon CPT1a inhibition with and without acetate (5 days). e. Heatmap and hierarchical clustering of top-scored downregulated genes of the NF-κB signaling pathway upon CPT1a deletion in 4T1 3D spheroids cultured in the presence of palmitate for 5 days, represented together with the expression status of the same genes upon acetate rescue and KAT2a deletion non-targeting sgRNA is used in control transfected samples (n=3). f. Proliferation of 4T1 cells upon genetic inhibition of either Cpt1a or Kat2a in 2D culture measured using incucyte. Mean of growth rate ± SEMis shown (n=6). One-way ANOVA with Dunnett’s multiple comparison test. g. 3D spheroids in 4T1 cells upon pharmacologic inhibition of either KAT2a using the inhibitor CPTH2 (2 μM) or CPT1A using etomoxir (50 μM) cultured for 5 days in medium with or without extra palmitate supplementation. Size quantification is represented by the average spheroids area of >100 spheroids. Data are presented as mean ± SEM (n ≥4). One-way ANOVA with Tukey’s multiple comparison test. h. 3D spheroids number per well of 4T1 cells upon palmitate supplementation (75 μM), CPT1a or KAT2a genetic inhibition performed by CRISPR (sgCpt1a and sgKat2a) compared to non-targeting sgScrambled as a control, and upon metabolic rescue by acetate (5mM). Data are presented as mean ± SEM (n≥4). One-way ANOVA with Tukey’s multiple comparison test. i. Dose-response of 3D spheroid growth to the pharmacologic inhibition of KAT2a using CPTH2 inhibitor with or without extra supplementation of palmitate. Left panel, representative pictures. Right panel, spheroid size quantification is represented by the average spheroids area of >100 spheroids (n ≥4). Two-way ANOVA with Tukey’s multiple comparison test. j. Total area and number of metastases in lung of mice after 14 days of intravenous (i.v.) injections with 4T1 Kat2a knockout (sgKat2a) or non-targeting sgScrambled control cells analyzed by H&E staining (n≥8). Unpaired two-tailed t-tests with Welch correction.

**Figure 7:**
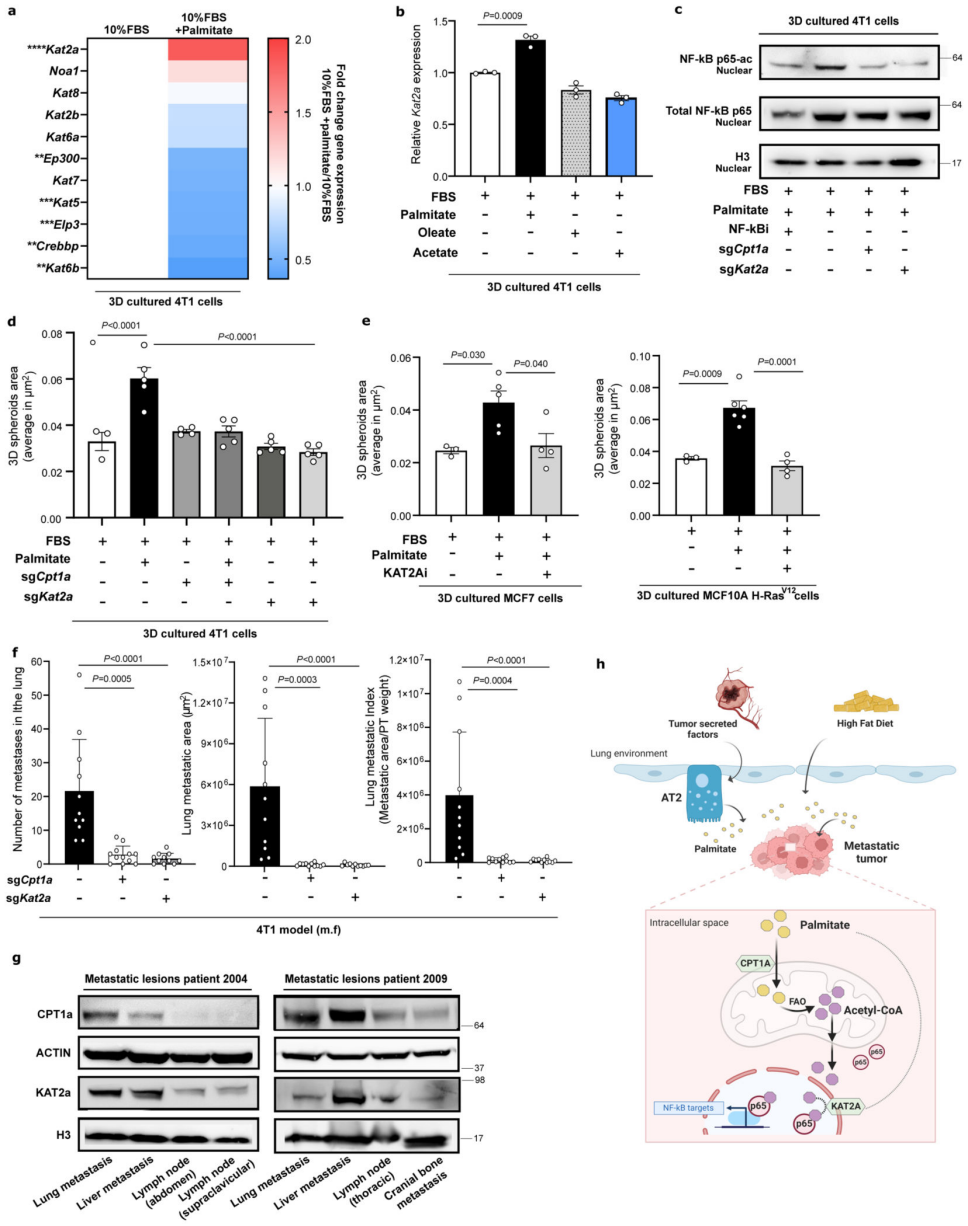
Silencing CPT1a and KAT2a inhibit metastasis formation *in vivo* and are co-expressed in palmitate-enriched environments. **a.** Differential expression of acetyltransferases in 4T1 3D spheroids in the with or without palmitate supplementation after 5 days of incubation. Data represent the fold changes compared with the non-supplemented condition (n=4). Unpaired two-tailed t-tests with Welch correction. Asterisks represent statistical significance *p < 0.01, ***p < 0.001, ****p < 0.0001. **b.** Relative *Kat2a* expression in 4T1 3D spheroids (5 days) with or without additional palmitate (75 μM), oleate (116 μM) or acetate (5 mM) (n =6). One-way ANOVA with Tukey’s multiple comparison test. **c.** Acetylated p65 (NF-κB p65ac) in nuclear extracts of 3D spheroids transduced with a lentiviral vector containing sg*Cpt1a*, sg*Kat2a* or sgcontrol, and cultured for 3 days in the presence of extra palmitate (75 μM) or the NF-κB inhibitor PDTC (0.5 μM). Histone H3 is shown as a loading control of NF-κB p65ac. A representative of n=3 experiments is shown. **d.** 3D spheroid growth of 4T1 cells silenced for *Cpt1a* or *Kat2a* compared to non-targeting sgRNA as a control, with or without extra palmitate (5 days). Data are presented as mean ± SEM (n ≥4). One-way ANOVA with Tukey’s multiple comparison test. **e.** 3D spheroid growth represented by the average spheroids area of >100 spheroids in MCF7 and MCF10A H-Ras^V12^ cells upon pharmacologic inhibition of KAT2a using the inhibitor CPTH2 (5μM) cultured for 5 days with or without palmitate supplementation. is. Data are presented as mean ± SEM (n ≥4). One-way ANOVA with Tukey’s multiple comparison test. **f.** Metastatic burden in lungs of mice injected with 4T1 into the mammary fat pad (m.f.) upon genetic knockout of *Cpt1a* and *Kat2a* compared to non-targeting sgRNA as a control. Data are presented as mean ± SEM (n ≥11). Data for control and *sgCpt1a* are also shown in Figure 4c. One-way ANOVA with Dunnett ’s multiple comparison test. **g.** CPT1a and KAT2a protein expression in different metastasis sites of breast cancer patients 2004 and 2009 from the UPTIDER program. A representative of n=2 experiments is shown. **h.** Tumor secreted factors and high fat diet are two independent factors increasing palmitate levels in the lung environment. Metastasizing cancer cells use the available palmitate to drive p65 acetylation in a CPT1a dependent manner resulting in pro-metastatic NF-κB signaling.

We asked how palmitate, but not other fatty acids, can increase p65 acetylation. One possibility is that a specific acetyltransferase mediates the palmitate-derived acetylation of p65. Thus, we compared the gene expression of several acetyltransferases in 3D cultured 4T1 cells with or without extra palmitate. Strikingly, we discovered that only lysine acetyltransferase 2a (KAT2a or GCN5) was significantly induced in the presence of palmitate (Figure 7a). Neither oleate nor acetate addition did increase KAT2a gene and protein expression (Figure 7b, Extended Data Figure 8a) although both increased acetyl-CoA levels (Extended Data Figure 8b). This observation suggests that this palmitatespecific effect is mediated by KAT2a.

**Extended Data Figure 9:**
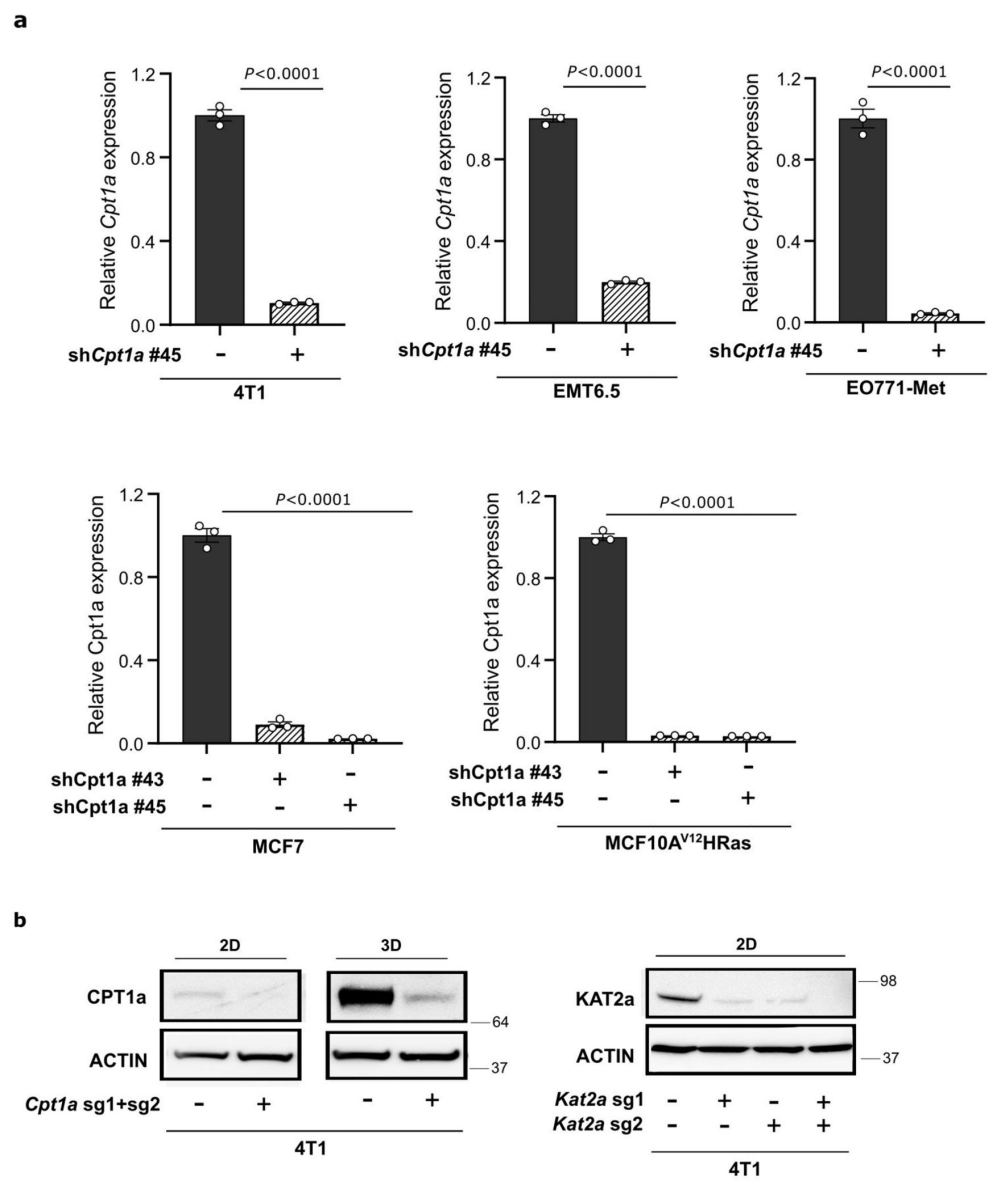
Protein and RNA expression of genetically modified breast cancer cells. a. Relative gene expression analysis of CPT1A in human (MCF10A H-RasV12 and MCF7), mouse (4T1, EO771-MCB3 and EMT6.5) breast cancer cells infected with either a control shRNA, or two different CPT1A, or Cpt1a shRNAs normalized to the control condition. Data are presented as mean ± SD (n=3). Unpaired two-tailed t-tests. b. Protein expression in mouse 4T1 cancer cells infected with either a non-targeting sgScrambled as a control or two different sgRNA against Cpt1a and Kat2a gRNAs. A representative of n=3 experiments is shown.

KAT2a has been mainly studied concerning histone H3K9 acetylation and some non-histone targets^36,37^. Therefore, we evaluated whether KAT2a could affect p65 indirectly via H3K9 acetylation by ChIP-sequencing. We found that the loss of either KAT2a or CPT1a (Extended Data Figure 9) only resulted in minor changes in H3K9-acetylation (Extended Data Figure 8c) that were not rescued by acetate (Extended Data Figure 8d). Despite that, a decrease in the transcription of NF-κB targets upon CPT1a or KAT2a deletion and rescue with acetate was observed (Extended Data Figure 8e). Consequently, we deemed it unlikely that KAT2a promotes metastasis via histone acetylation.

Next, we investigated whether KAT2a directly acetylates p65, since a physical interaction was shown during hippocampal memory regulation ^38^. We deleted KAT2a in 4T1 cells and assessed the presence of K310 acetylated p65 in the nucleus. As observed with CPT1a deletion, total p65 and general binding to DNA were not significantly altered (Figure 7c, Extended Data Figure 7f). However, in line with the hypothesis that KAT2a is responsible for acetylating p65 in the presence of palmitate, we observed that its deletion decreased the amount of p65-K310ac in the nucleus (Figure 7c). Subsequently, we investigated whether blocking KAT2a and thus p65 acetylation, prevents the palmitate-induced spheroid growth. KAT2a inhibition did not decrease proliferation of cancer cells growing in 2D (Extended Data Figure 8f). However, KAT2a deletion reduced 3D 4T1 spheroid size and number to a similar degree as CPT1a deletion (Figure 7d, Extended Data Figure 8g-h). Accordingly, 3D growth of 4T1, MCF7 and MCF10A H-Ras^V12^ was significantly reduced upon treatment with the KAT2a inhibitor cyclopentylidene-[4-(4’-chlorophenyl)thiazol-2-yl)hydrazone (CPTH2; 2 μM for 5 days) ^39^ (Figure 7e, Extended Data Figure 8g). Moreover, breast cancer spheroids showed sensitivity to CPTH2 only in the presence of extra palmitate but not in the absence of extra palmitate (Extended Data Figure 8i). Based on these data we concluded that KAT2a acetylates p65 in the presence of palmitate and that this activity is essential for palmitate-promoted spheroid growth.

### KAT2a deletion impairs metastasis formation

Next, we assessed whether blocking KAT2a and hence p65 acetylation is sufficient to impair lung metastasis formation. We injected 4T1 control and KAT2a knockout cells (Extended Data Figure 9) into the mammary fat pad of mice or intravenously and assessed lung metastases number, area and metastatic index based on H&E staining. We observed that metastatic burden was dramatically reduced in the absence of KAT2a expression compared to control (Figure 7f, Extended Data Figure 5a), while primary tumor growth showed only a small reduction compared to control (Extended Data Figure 5b). A similar reduction in metastases area was observed upon intravenous injection of cancer cells (Extended Data Figure 8j). Thus, we concluded that blocking KAT2a phenocopies CPT1a inhibition and is sufficient to impair metastatic growth in mice.

Finally, we investigated whether this CPT1a-KAT2a-driven mechanism may occur in patients with metastatic breast cancer. We argued that metastases growing in palmitate-enriched organs require co-expression of CPT1a and KAT2a. We therefore collected several metastatic lesions of different organs from two patients with breast cancer within the UPTIDER rapid autopsy program (Supplementary Table 2) and determined CPT1a and KAT2a protein expression. Consistent with the determined mechanism, we observed that CPT1a and KAT2a proteins were highly co-expressed within lung and liver metastases compared to cranial bone and lymph node metastases (Figure 7g, h). We therefore concluded that CPT1a and KAT2a may act in concert in patients with breast cancer to promote metastatic growth specifically in organs those interstitial nutrient composition is palmitate enriched, with the latter being an oleate-enriched organ^95^.

In conclusion, we identify a palmitate priming of the lung during pre-metastatic niche formation and high fat diet exposure. Metastasizing cancer cells rely on CPT1a-dependent palmitate oxidation to acetyl-CoA, which in turn serves as a substrate for the acetylation of p65 by KAT2a. Subsequently the pro-metastatic NF-κB transcriptional program is activated supporting metastatic growth in palmitate-rich environments (Figure 7h).

## Discussion

Here, we provide the first analysis of lipid availability in the interstitial fluid of lungs and livers in healthy and pathological conditions. Furthermore, we functionally linked palmitate-rich environments to protein acetylation upstream of a pro-metastatic NF-κB signaling. Palmitate can induce a prometastatic memory in primary tumors via histone H3 lysine 4 trimethylation stimulating intratumor Schwann cells and innervation ^40^. Adding to this known mechanism we discovered an epigenetically independent role of palmitate in promoting metastasis formation.

Palmitate and obesity are associated with inflammatory NF-κB signaling in stromal cells^41–44 45^ and, in cancer cells, it was suggested that etomoxir reduces phosphorylation of iκBα ^46^. Here, we discovered a mechanism by which CPT1a-dependent palmitate oxidation regulates NF-κB signaling via acetylation of p65. Moreover, we find that the acetyltransferase KAT2a is only important for p65 acetylation in the presence of extra palmitate. This provides strong evidence of a nutrient environment-dependent regulation of protein acetylation in organs of metastasis.

Acetyl-CoA levels are linked Smad ^47^ and histone ^48–50^ acetylation with subsequent breast cancer metastasis ^47,48^. In the liver, high fat diet exposure elevates acetyl-CoA levels which increases p65 acetylation and its transcriptional activity modulating inflammatory responses ^51^. We add to this current knowledge by defining p65 as a KAT2a-acetylation target modulated by palmitate-derived acetyl-CoA in metastases. Contrary to a previous study showing that KAT2a participates in p65/RelA degradation independently of its acetyltransferase activity ^52^, and in line with the previously reported interaction of KAT2a and p65 activating gene expression via acetylation ^38^, we find that KAT2a activity is needed to activate NF-κB signaling in palmitate-rich environments.

Several roles of fatty acid oxidation were described in metastasizing cancer cells^1,2^. CPT1a deletion inhibits mammosphere formation of luminal cells ^54^, and a splice variant of CPT1a promotes histone deacetylase (HDAC) activity through a protein–protein interaction ^55^. Moreover, ATP levels sustained by CPT1a activity activate SRC through autophosphorylation ^56^. Beyond breast cancer, matrix detached colorectal and ovarian cancer cells accumulate ROS upon CPT1a silencing ^57^ and oxidize oleate to sustain mitochondrial respiration ^58^, respectively. Similarly, oleate enriched lymph node metastases from melanoma rely on fatty acid oxidation ^59^. We complement these various functions of fatty acid oxidation by showing that palmitate oxidation is important for p65 acetylation. Moreover, we show that CPT1a silencing is sufficient to target metastasis formation in lean and high fat diet fed mice, with the latter not being shown *in vivo* before. This may suggest that the use of the CPT1a inhibitor etomoxir should be revisited in obese breast cancer patients.

To date, very little is known about the nutrient priming of the pre-metastatic niche by primary tumor-secreted factors with only one report highlighting spared glucose consumption through the induction of a hypometabolism of resident cells ^7^. We discover that palmitate availability is selectively increased in pre-metastatic lungs because palmitate is actively secreted by lung resident AT2 cells in response to tumor secreted factors. High fat diet on the opposite alters the lung and liver environment by increasing the availability of several fatty acids in the interstitial fluid, which may be reflective of the systemic increase that was observed in circulation ^23^. Obesity is known to promote breast cancer progression through leptin and insulin signaling^64^. Adding to this, we show that high fat diet increases lung interstitial fatty acid availability even though lungs are not considered steatotic organs. Thus, this suggests a much broader impact of diet on nutrient availability than previously considered and highlights an underappreciated role of nutrient priming of distant organs in metastasis formation.

## Methods

### Cell culture

MCF10A (ER-/PR-/HER2-), MCF7 (ER+/PR+/HER2-) and 4T1 (ER-/PR-/HER2-) cell lines were purchased from ATCC. The EMT6.5 (ER-/PR-/HER2-) cell line was provided by R. Anderson (Peter MacCallum Cancer Center), EO771-Met clone (ER-/+/PR-/HER2-) by Prof. Mazzone VIB Center for Cancer Biology) and 4T07 (ER-/PR-/HER2-) cell line by Prof. Gomes (H Lee Moffitt Cancer Center). MCF10A cells expressing the oncogenic driver H-Ras^V12^ (MCF10A H-Ras^V12^) were generated as previously described^19^ as a relevant in vitro model for breast cancer since 50% of the human breast cancers display increased H-Ras activity ^61^. Moreover, the suitability of this cell line for metastasis biology has been previously reported ^19,20,62,63^. 4T1, 4T07 and EMT6.5 cells were grown in Roswell Park Memorial Institute (RPMI) 1640 medium supplemented with 10% fetal bovine serum, 1% penicillin (50 units/mL and 1% streptomycin (50 μg/mL). MCF7 cells were maintained in Dulbecco’s modified Eagle’s medium (DMEM) supplemented with 10% fetal bovine serum (FBS), 1% penicillin (50 units/mL) and 1% streptomycin (50 μg/mL). MCF10A H-RAS^V12^ cells were cultured in DMEM/F12 supplemented with 5% horse serum, 1% penicillin (50 units/mL, 1% streptomycin (50 μg/mL), 0.5 μg/mL hydrocortisone, 100 ng/mL cholera toxin, 10 μg/mL insulin and 20 ng/mL recombinant human EGF. All cells were maintained at 37°C and 5% CO_2_ and 95% relative humidity and regularly tested negative for mycoplasma infection by Mycoalert detection kit (Lonza). For 3D growth conditions, plates covered with soft-agar were used as described^19^. Briefly, 1% soft-agar was mixed 1:1 with culture medium and left to solidify at room temperature. Cells were plated on top of the base agar and incubated for 3-5 days.

Sodium acetate was purchased from Sigma-Aldrich and used at a concentration of 5 mM. Sodium palmitate, stearic acid, oleic acid and octanoic acid were purchased from Sigma-Aldrich. These free fatty acids (FFA) were supplemented on top of FBS to the media in different concentrations up to complete a final concentration of 130 μM. FFA stock solutions were prepared by coupling free fatty acids with bovine serum albumin (BSA) as described previously ^64^. The final ratio of FFA to BSA was always at least 3:1. Conditions with no additional FFA added were prepared using the same stocks of 10% w/w BSA with an equivalent amount of ethanol matching the concentration in FFA stocks.

Etomoxir and the KAT2A specific inhibitor cyclopentylidene-[4-(4’-chlorophenyl)thiazol-2-yl)hydrazone (CPTH2)^65^ were purchased from Cayman Chemical, dissolved in ethanol and used at concentrations of 50 and 2 μM, respectively. The ATP Citrate lyase (ACLY) inhibitor BMS-303141 was purchased from MedChemExpress, dissolved in DMSO and used at 20 μM. Tumor Necrosis Factor-α from mouse was purchased from Sigma-Aldrich and used at 10 ng/μL. Ammonium pyrrolidinedithiocarbamate (PDTC) was purchased from Merck, dissolved in water and used at 0.5 μM.

### Cell proliferation assays

Growth was assessed based on cell number and cell confluency (two-dimensional, 2D) or spheroid size (three-dimensional, 3D). Specifically, to measure cell proliferation in 2D cultures, growth rates were calculated by measuring confluency every 4 hours using an Incucyte live-cell imager (Essen Biosciences, Ann Arbor, MI) over 96 hours in culture. To measure 3D growth, spheroids were cultured in 6-well plates upon specified conditions for 5 days. The compounds were always added on day 0 and representative pictures of each well were taken on day 5. Microscopy images were acquired using Motic Images Plus 2.0 software (Motic). Spheroids area was analyzed using Image Studio Lite 5.2 of ≥5 representative pictures per experimental condition. All growth experiments were performed in n≥3 biological replicates.

### Generation of shRNA knockdown cell lines

CPT1a knockdown cell lines were generated using the shRNA-expressing lentiviral pLKO-shRNA2 vector (No. PT4052-5; Clontech), with a puromycin selection cassette, kindly provided by Prof. P.Carmeliet, VIB, Belgium). Two non-overlapping oligonucleotide sequences per species (human and mouse) and nonsense scrambled shRNA sequence as negative control were used (oligonucleotide sequences are available upon request). Lentiviral particles were produced in HEK293 cells. Transduction of cells was performed overnight and the medium was replaced the next day. Polyclonal cells were selected for one week with puromycin, 1 μg/mL for MCF10A H-Ras^V12^, MCF7 and EMT6.5 and 2 μg/mL for 4T1 cells. shRNA-based silencing was confirmed by qPCR (Extended Data Figure 9a).

### Generation of CRISPR knockout cell lines

Murine *Cpt1a* and *Kat2a* knockout cell lines were generated by first establishing parental cell lines with doxycycline-inducible Cas9 expression using the Dharmacon Edit-R Inducible Lentiviral Cas9 vector (Horizon Discovery). Complementary sgRNA oligonucleotides targeting two different exons of either *Cpt1a* or *Kat2a* were designed and cloned into the LentiGuide Puro sgRNA expression vector (Addgene plasmid #52963) and delivered to cells via lentiviral infection. Targeting sequences - mouse Cpt1a sgRNA1 (CACATTGTCGTGTACCACAG), mouse Cpt1a sgRNA2 (ACGTTGGACGAATCGGAACA), mouse Kat2a sgRNA1 (GCTTCGGCCAAACACGTGGG), mouse Kat2a sgRNA2 (GCTCGCCTGGAAGAACGGCG), and non-targeting sgRNA against EGFP (GAGCTGGACGGCGACGTAAA) - were synthesized by IDT. Lentiviral particles were produced in HEK293T cells. Stable 4T1 cell lines were generated by selection with puromycin (2 μg/mL). Cas9 expression was induced using doxycycline (2ug/ml) for 7 days after which gene knockout was verified by western blot (Extended Data Figure 9b).

### Gene expression analysis by quantitative PCR

Total RNA from frozen tissues or cell lines was isolated using TRIzol™ Reagent (Thermo Scientific) and cDNA synthesis was performed using qScript cDNA Synthesis Kit (Quanta) according to the manufacturer’s protocols. 50 ng of cDNA per reaction were amplified at 95 °C for 10 min, followed by 40 cycles of 15 s at 95 °C and 1 min at 60 °C using SYBER Green PCR Master Mix (Life Technologies) in the 7500HT system (Applied Biosystems). The mRNA expression in each sample was normalized to endogenous housekeeping gene *RPL19* and relative expression was analyzed using 2^-ΔΔCt^ method. Gene-specific primers were designed using Primer3 or obtained from Origene. Sequences are listed in Table 1.

### Cell-fractionation and nuclear isolation

Nuclear and cytoplasmic extracts were prepared from spheroids as described previously ^66^. Briefly, spheroids were collected and washed once in ice-cold PBS. Spheroid pellets were then suspended in 0.5 ml of hypotonic cytoplasmic fraction buffer (20 mM HEPES pH 8.0, 0.2 % IGEPAL CA-630, 1 mM EDTA, 1 mM DTT, protease and phosphatase inhibitor cocktail), lyse using tissue-lyser and incubated on ice for 10 min with occasional shaking. After centrifugation (4 °C, 1700 × g, 5 min), supernatant was set aside and treated as the cytoplasmic fraction. The remaining pellet was suspended in 100 μl of nuclear fraction buffer (20 mM HEPES pH 8, 420 mM NaCl, 20 % glycerol, 1 mM EDTA, 1 mM DTT, protein and phosphatase inhibitors), incubated on ice for 30 min with occasional mixing and then centrifuged at 4 °C, 10,000 × g, 10 min. Supernatant was transferred to a new tube and treated as nuclear fraction. Protein concentration was measured using Bradford Assay.

### Protein extraction and western blot analysis

Spheroids were collected by centrifugation and lysed in RIPA lysis buffer (Thermo Scientific) supplemented with protease (complete, Mini Protease Inhibitor Cocktail Tablets (Roche) and phosphatase (PhosSTOP™, Sigma) inhibitors. Tissues were ground prior RIPA incubation. Protein amount was measured using a Pierce BCA protein assay kit (Thermo Scientific). Aliquots of 30-40 μg of protein were loaded on a NuPAGE 4–12% denaturing Bis-Tris gel and transferred to a nitrocellulose membrane (Thermo Scientific). Membranes were incubated for 1h at room temperature in a blocking solution of 5% milk in TRIS Buffer Saline 0.05% Tween (TBS-T). Subsequently, membranes were incubated overnight at 4°C with the following primary antibodies: CPT1a (Abcam, ab234111; 1:1000 dilution), GCN5L2/KAT2A (C26A10) (Cell signaling, 3305T; 1:1000 dilution), NF-kB p65 (acetyl K310) (Abcam, ab19870; 1:1000 dilution), NF-κB p65 (D14E12) (Cell signaling, 8242S; 1:1000 dilution), β-actin (Sigma, A5441; 1:10,000 dilution) or Histone H3 (Abcam, ab1791; 1:2000 dilution). For NF-kB p65-ac analysis, total levels of NF-kB were assessed in parallel samples run in the same gel prepared from the same master mix. The day after the membranes were incubated with a mouse (Cell Signaling Technology, 7076; 1:4000 dilution) or rabbit (Cell Signaling Technology, 7074; 1:4000 dilution) HRP-linked secondary antibodies, and bound antibodies were visualized using Pierce ECL reagent (Thermo Scientific). Images were acquired using an ImageQuant LAS 4000 (GE Healthcare).

### Electrophoretic Mobility Shift Assays (EMSA)

Whole extracts from 3D spheroids were prepared and analyzed for DNA binding activity using the HIV-LTR tandem κB oligonucleotide as κB probe as described previously^67^. The use of the whole cell extracts to analyze NF-κB DNA-binding status by EMSA was previously validated by comparing nuclear and total NF-κB DNA-binding profiles and the observation of highly similar NF-κB DNA-binding activity, indicating that NF-κB binding emanates from nuclear proteins ^68^.

### Mitochondrial mass

To measure mitochondrial mass, 3D spheroids were collected and dissociated using trypsin. Single cells were washed once with PBS and stained with MitoTracker Deep Red (#M22426, Invitrogen). Cells were incubated with pre-warmed MitoTracker staining solution (diluted in PBS/HBSS buffer with 1 μM Mitotracker) for 30 min at 37 °C. All subsequent steps were performed in the dark. Cells were washed in PBS, and re-suspended in 300 μL of PBS. Cells were then analyzed by flow cytometry. Data analysis was performed using FlowJo software.

### Mass spectrometry analysis

To analyze metabolite abundances of 3D spheroids, quenching and extraction procedures were applied as described previously ^69^. Briefly, spheroids growing in 6-well plates were collected after 5 days by centrifugation (1.5 min at 350 x g). Pellets were quenched by transferring them to a tube placed inside of a rack in a cold ethanol bath (–40°C) that contained cold quenching solution (60% methanol, 10% ammonium acetate (100 mM) and 30% Milli-Q). Spheroids were spun down (1 min at 2500 rpm), washed using –20°C cold quenching solution and placed in dry ice until continuing with the extraction. To analyze two-dimensional (2D) cultured cells, cells were seeded in 6-well plates and, after 5 days, cells were washed with blood bank saline and quenched by flash-freezing the plates in liquid nitrogen. Metabolites for mass spectrometry analysis were extracted, derivatized and measured as described previously ^70,71^. Briefly, for 2D cells, 800 μL of –20 °C cold 62.5% methanol-water buffer containing glutarate as internal standard (2.5 μg/mL) was added to the quenched wells and cells were scraped using a pipette tip. Suspensions were transferred to Eppendorf tubes and 500 μL of –20°C cold chloroform containing heptadecanoate (10 μg/mL) as internal standard was added. For 3D spheroids, 800 μL of –20 °C cold 62.5% methanol-water buffer was added into Eppendorfs containing the quenched spheroids and a tissue-lyser was applied inside of the cold ethanol bath (–40°C) until the spheroids were completely disintegrated. After, 500 μL of –20°C cold chloroform was added. 2D and 3D samples were vortexed at 4°C for 10 min to extract metabolites. Phase separation was achieved by centrifugation at 4°C for 10 min max x g, after which the chloroform phase (containing the total fatty acid content) and the methanol phase (containing polar metabolites) were separated and dried by vacuum centrifugation.

Metabolite abundances were analyzed by either gas or liquid chromatography-mass spectrometry. For fatty acid measurements, the lipid fraction (chloroform phase) was esterified with 500 μL 2% sulfuric acid in methanol at 60°C overnight and extracted by addition of 600 μL hexane and 100 μL saturated aqueous NaCl. Samples were centrifuged for 5 min and the hexane phase was separated and dried by vacuum centrifugation. Samples were resuspended in hexane and fatty acids were separated with gas chromatography (8860 or 7890A GC system, Agilent Technologies, CA, USA) combined with mass spectrometry (5977B or 5975C Inert MS system, Agilent Technologies, CA, USA). 1 μL of each sample was injected in splitless mode, split ratio 1 to 3 or 1 to 9 with an inlet temperature of 270 °C on a DB-FASTFAME column (30m x 0.250 mm). Helium was used as a carrier gas with a flow rate of 1 mL per min. For the separation of fatty acids, the initial gradient temperature was set at 50°C for 1 min and increased at the ramping rate of 12°C/min to 180°C, followed by a ramping rate of 1°C/min to rich 200°C for 1 min. The final gradient temperature was set at 230°C with a ramping rate of 5°C/min for 2 min. The temperatures of the quadrupole and the source were set at 150°C and 230°C, respectively. The mass spectrometry system was operated under electron impact ionization at 70 eV and a mass range of 100-600 amu was scanned. For acetyl-CoA and polar metabolites measurements, the polar metabolite phase was resuspended in 50 μL water and analyzed in a Dionex UltiMate 3000 LC System (Thermo Scientific) with a thermal autosampler set at 4°C, coupled to a Q Exactive Orbitrap mass spectrometer (Thermo Scientific) and a volume of 10 μL of sample was injected on a C18 column (Acquity UPLC HSS T3 1.8μm 2.1 x 100mm). The separation of metabolites was achieved at 40°C with a flow rate of 0.25 ml/min. A gradient was applied for 40 min (solvent A: 10mM Tributyl-Amine, 15mM acetic acid – solvent B: Methanol) to separate the targeted metabolites (0 min: 0% B, 2 min: 0% B, 7 min: 37% B, 14 min: 41% B, 26 min: 100% B, 30 min: 100% B, 31 min: 0% B; 40 min: 0% B. The MS operated in negative full scan mode (m/z range: 70-500 and 190-300 from 5 to 25 min) using a spray voltage of 4.9 kV, capillary temperature of 320°C, sheath gas at 50.0, auxiliary gas at 10.0. Data was collected using the Xcalibur software (Thermo Scientific). For the detection of metabolites by LC-MS/MS, a 1290 Infinity II with a thermal autosampler set at 4°C, coupled to a 6470 triple quadrupole (Agilent Technologies) was used. Samples were resuspended in 80% methanol-water and a volume of 4 μL was injected on a SeQuant ZIC/pHILIC Polymeric column (Merck Millipore). The separation of metabolites was achieved at 25°C with a flow rate of 0.20 ml/min. A gradient was applied for 22 min (solvent A: 10 mM ammonium acetate (pH=9.3, 10 mM) – solvent B: acetonitrile) to separate the targeted metabolites (0 min: 10% A, 2 min: 10% A, 13 min: 30% A, 13.1 min: 70% A, 17 min: 75% A, 18 min: 10% A, 22 min: 10% A). The temperature of the gas and the sheath gas were set at 270°C (flow: 10L/min) and 300°C (flow: 12L/min), respectively.

Metabolite abundances were calculated from the raw chromatograms using an in-house MATLAB script. For relative abundance, total ion counts were normalized to an internal standard (glutarate or heptadecanoate) and the protein content for cell extracts. To calculate fatty acid concentration in interstitial fluid, a known volume of interstitial fluid was extracted using 62.5% methanol-water and chloroform buffers as described above. A standard curve for each metabolite was extracted and analyzed in parallel.

### ^13^C-metabolite labeling

Cells were seeded in 3D conditions using 11 mM ^13^C_6_-Glucose (Cambridge Isotope Laboratories) dissolved in RPMI (XXX, ThermoFisher) + 10% dialyzed FBS. Spheroids grew for 5 days and then quenching and metabolite extraction were performed as described above. Metabolite abundances and ^13^C labeling patterns were analyzed by either gas chromatography–mass spectrometry. Mass distribution vectors were extracted from the raw ion chromatograms using Matlab, corrected for naturally occurring isotopes using the method of Fernandez et al ^72^, and fractional contribution of carbon was calculated as described by Buescher et al ^73^. Palmitate uptake was calculated using the labeled substrate ^13^C16-Palmitic acid and measuring the difference in labeled palmitate in media after 5 days. Palmitate normalized total ion counts from media were corrected by total protein content of each analyzed well. Fractional *de novo* synthesis of fatty acids was calculated using Isotopomer Spectral Analysis (ISA) during the exposure to the labeled substrate ^13^C_6_-Glucose for 5 days.

### Histone extraction and SILAC analysis

For the preparation of the SILAC labeled histone internal standard, 4T1 cells were cultured in SILAC DMEM (Silantes) supplemented with 10% 10 kDa dialyzed FBS (PAA), 2 mM glutamine, 1% penicillin/streptomycin, 84 mg/l 13C615N4 L-arginine and 175 mg/l 13C615N2 L-lysine (Cambridge Isotope Laboratories) until they were fully labeled. 3D spheroids were collected after 5 days and the core histone proteins were isolated by acid extraction procedure previously described before ^74^. Briefly, nuclei were isolated from pelleted cells with a high salt extraction buffer (15 mM Tris-HCL (pH 7.5), 60 mM KCl, 15 mM NaCl, 5 mM MgCl2, 1 mM CaCl2, 250 mM Sucrose, 0.3% NP-40). Histones were isolated from nuclei by incubation in 0.4 N H2SO4, followed by addition of trichloroacetic acid to a final concentration of 20%. Isolated histones were precipitated with acetone and resuspended in 8 M urea. SILAC labeled histones were added to unlabelled histones as an internal standard in a 2:1 ratio. Histones were digested in 50 mM ammonium bicarbonate using Arg-C (Promega), and the resulting peptides desalted by C18 StageTip ^75^. Peptides were resuspended in 1% TFA, 0.2% formic acid buffer and injected on an EASY-nLC (Thermo Fisher Scientific) coupled online to a mass spectrometer. Peptides were separated on a 20 cm fused silica emitter (New Objective) packed inhouse with reverse-phase Reprosil Pur Basic 1.9 μm (Dr. Maisch GmbH). Peptides were eluted with a flow of 300 nl/min from 5% to 30% of buffer B (80% acetonitrile, 0.1% formic acid) in a 60 min linear gradient. Eluted peptides were injected into an Orbitrap Fusion Lumos (Thermo Fisher Scientific) via electrospray ionization. MS data were acquired using XCalibur software (Thermo Fisher Scientific). The MS .raw files were processed with MaxQuant software (version 1.6.6.3) and searched with the Andromeda search engine with the following settings: minimal peptide length 6 amino acids, fixed modification Carbamidomethyl (C) and variable modifications Acetyl (K), Acetyl (Protein N-term) and Oxidation (M). Multiplicity was set to 2, where the light labels were Arg0 and Lys0 and the heavy labels were Arg10 and Lys8. The false discovery rates (FDRs) at the protein and peptide level were set to 1%. Perseus (version 1.6.2.2) was used for downstream analysis. The data were filtered to remove potential contaminants, reverse peptides which match a decoy database, and proteins only identified by site. To ensure unambiguous identification, only proteins identified with at least one unique peptide were considered. The heavy/light SILAC ratios were transformed by 1/x and then by log2. Each acetylation site was normalized by total histone abundance.

### ChIP sequencing analysis

To analyze changes in genome-wide DNA binding sites using, we applied ChIPmentation protocol followed by sequencing as described previously ^94^. Briefly, 3D spheroids were collected after 5 days, cross-linked with 1% formaldehyde for 10 min at room temperature and then quenched by addition of glycine (125 mM final concentration). Nuclei were isolated and resuspended in L3B+ buffer (10 mM Tris-Cl pH 8.0, 100 mM NaCl, 1 mM EDTA, 0.5 mM EGTA, 0.1% sodium deoxycholate, 0.5% N-Lauroylsarcosine, 0.2% SDS). Chromatin was fragmented to 100-300 bp using 25 cycles (30 sec on, 30 sec off, 20% amplitude) using Qsonica Q800R. Chromatin immunoprecipitation was performed overnight in the presence of H3K9ac antibody (Cell signaling, 9649S, 1:50 dilution) conjugated to magnetic protein A/G beads (Millipore). Drosophila melanogaster chromatin was spiked-in (50 ng, Active Motif, 53083). Next, tagmentation (Nextera DNA Sample Preparation Kit, Illumina) and library preparation was performed, then DNA was purified using three sided SPRI bead cleanup using 1.0X; 0.65X; 0.9X ratios (Agencourt AMPure Beads, Beckman Coulter) and sequenced on Illumina Hiseq 4000 platform (Illumina, San Diego, CA, USA). The raw sequencing reads were cleaned with fastq-mcf and a quality control was performed with FastQC. These cleaned reads were then aligned to the mm10 genome using BWA and duplicates were removed with Picard (v1.130). deepTools (v1.6) was used to plot heatmaps of signals centered around TSS as well as to plot Spearman correlation of ChIP-seq signal per gene between conditions.

### *In vitro* invasion assay

50,000 cells were embedded in a 50:50 mix of growth factor-reduced Matrigel (BD Biosciences) and collagen I (Life Technologies) and seeded onto 35 mm glass bottom culture dishes (MatTek). Cells were allowed to invade for 72h, and then they were stained with calcein green (Life Technologies) for 1h, washed with PBS and immediately imaged. Imaging was performed on a Leica TCS SP8 X confocal microscope equipped with a White Light Laser and a HCX PL APO CS 10x/0.40 DRY objective. Images were acquired as three-dimensional scans with 10 μm Z-steps and processed with LAS X 3.3 software (Leica) to obtain maximum projection images. Quantification of invasive area and invasive distance was performed on the maximum projection images using FIJI 2.3.1 distribution of ImageJ2.3.0.

### *In vivo* mouse experiments

All animal experiments complied with ethical regulations and were approved by the Institutional Animal Care and Research Advisory Committee of KU Leuven, Belgium (ECD number P007/2020, P048/2020, and ECD P025/2020). All mice were housed under a regimen of 12 h light/12 h dark cycles in non-SPF (conventional) conditions with a constant supply of food and water. Sample size was determined using power calculations with B = 0.8 and P < 0.05 based on preliminary data and in compliance with the 3R system: Replacement, Reduction, Refinement. For lung metastasis experiments, 6-8 weeks old female Balb/cAnNCrlCrlj and BALB/cOlaHsd mice were inoculated with 4T1 or EMT6.5 cells either in the mammary fat pad (m.f.; 1 × 10^6^ cells) or intravenously (i.v.; 1 × 10^5^ cells). For EO771 cells, 6-8 weeks old female C57BL/6JAXmice were used to inject i.v. 1 × 10^5^ cells. Mice were sacrificed after 21 days (m.f) or 13 days (i.v) using ketamine-xylene or dolethal. For injections performed after lung pre-conditioning with tumor conditioned media, 4T1 and 4T07 cells were injected i.v (2.5× 10^4^ cells) and mice sacrificed after 16 days. For etomoxir treatment after breast cancer cell seeding in the lung, 1 × 10^5^ cells were injected i.v. and, after 4 days, intraperitoneal injections of etomoxir (40 mg/kg per day) or vehicle (water) were daily performed. Mice were sacrificed after 10 days and lung metastatic growth was assessed by flow cytometry. For acute etomoxir treatment, 1x10^6^ 4T1 cells were suspended in 50 μL of PBS and injected into the mammary fat pad of BALB/c mice. Mice were checked for tumor formation by palpation, and when tumors were about 13 mm, mice were randomized into two groups for daily intraperitoneal injections for 3 days: vehicle (water) and treatment (etomoxir, 40 mg/kg per day). Mice were sacrificed and lung metastases were collected to further analysis. For metabolomics, mice were euthanized using dolethal, and tumor tissues were placed in cold saline for rapid dissection (>3 min) and immediately frozen using a liquid-nitrogen-cooled Biosqueezer (Biospec Products). The tissue was weighed (10–15 mg) and pulverized (Cryomill, Retsch) under liquid-nitrogen conditions. To follow the effect on the primary tumor, tumor volumes were measured during the experiment using a caliper. Additionally, at the end of the experiment, primary tumors were dissected and weighted. Humane endpoints were determined using a scoring sheet to determine the condition of the mouse as follows: tumor size of 1.8 cm^3^, loss of ability to ambulate, labored respiration, surgical infection, or weight loss over 10% of initial body weight. Mice were monitored and upon detection of one of the previously mentioned symptoms, the animal was euthanized. For all experiments, the maximum permitted tumor volume was 1.8 cm^3^and this limit was not exceeded in any experiment.

For high fat diet experiments, 4- weeks old female BALB/c mice were randomized into two groups: control diet (CD, E15742-33 ssniff Spezialdiäten GmbH) or long-chain high fat diet (HFD, S8655-E220 sniff Spezialdiäten GmbH). The energy balance between fat (from lard)/protein/carbohydrates was 13%/27%/60% and 60%/20%/20% for CD and HFD, respectively. Mice were maintained in these diets for 16 weeks before the experiments were performed. Bodyweight was monitored biweekly and mice were inspected weekly for welfare assessment. For lung metastasis experiments, 1×10^5^ cells were injected i.v and mice were sacrificed 11 days after. For liver metastasis experiments, 1×10^5^ cells were delivered via the splenic vein and mice were sacrificed 17 days after.

### Lung and liver interstitial fluid extraction

Interstitial fluid extraction method was adapted and performed as described previously^8,9^. For the collection of human samples, ‘normal’ lung tissue was collected from patients who underwent lung surgery for emphysematous lung volume reduction or tumorectomy (taken as far away as possible from the tumor front). The study was approved by the local ethics committee (Medical Ethics Committee UZ/KU Leuven) under the protocol S57123. For the collection of mice samples, 6-8 balb/c female mice were first euthanatized with 50μL of 60mg/ml of Dolethal (pentobarbital sodium), and subsequently, liver and lung were harvested by surgical resection, washed with a blood bank saline solution and dried from liquid excess by carefully tapping in a gauze. Subsequently, the organs were placed in a homemade filtered centrifuge tube with a nylon mesh filter with 20 μm opening pores (Repligen). The interstitial fluid (between 1-5 μL for each organ) was collected in the columncentrifuge tubes by centrifugation at 400g, 4°C for 10 minutes and stored in dry ice immediately after extraction. The volume of interstitial fluid was used to determine the concentration of the different metabolites measured by mass spectrometry.

### Tumor condition media generation and *in vivo* pre-metastatic niche formation

The procedure to induce pre-metastatic niche formation in the lung was adapted from ^12^. In brief, for primary tumor formation, 1x10^6^ 4T1 cells were injected orthotopically in the mammary fat pad of BALB/c mice (7-8 weeks old). After 21 days, mice were euthanized and primary tumor was removed. Tumors were cut into small pieces and placed in 10-cm dishes cultured with 15 mL/g of tumor of DMEM (No FBS) + 1% penicillin (50 units/mL) and 1% streptomycin (50 μg/mL). Tumors were incubated 72h at 37°C and 5% CO_2_. The same media was incubated in parallel in 10cm dishes without tumor to generate control media. After 72h, tumor- and control- conditioned media were collected, transferred to a cell strainer (70 μm) and spin down for 10 min at 1000 x g. Supernatants were moved to 50 mL tubes by pooling together conditioned media of the same type from 3 different tumors to cover the variability between tumors. Hepes (20mM) was added to the media and this was filtered (0.20 μm) and aliquoted to be stored at 4*C no more than 2 weeks after collection. 7-8 weeks old BALB/c mice were injected intravenously with 200 μL of either control or tumor conditioned media 3 times per week for 3 weeks. Three days after the last injections, interstitial fluid of the lung was collected or cancer cells were injected as described above.

### Haematoxylin and eosin staining of tumor sections

Haematoxylin and eosin (H&E) staining of pulmonary metastases was performed as previously described^20^. Briefly, dissected lung samples were gently infused via the trachea with 10% formalin, fixed overnight, and immersed in 70% ethanol for 24 hours. Then, 5-μm thick sections obtained from the resulting paraffin blocks were stained with haematoxylin and eosin. Scanned slides were analyzed for metastatic area and the number of metastases using ZEN blue software. Metastatic burden is determined by analyzing the number of metastases, metastatic area, and metastatic index. Metastatic index was calculated by dividing the metastatic area by the primary tumor weight. Only mice with a primary tumor of 0.8 g or greater were analyzed. All i.v.-injected animals were analyzed. All samples were analyzed blinded.

### MALDI-MSI molecular imaging

**Extended Data Figure 10:**
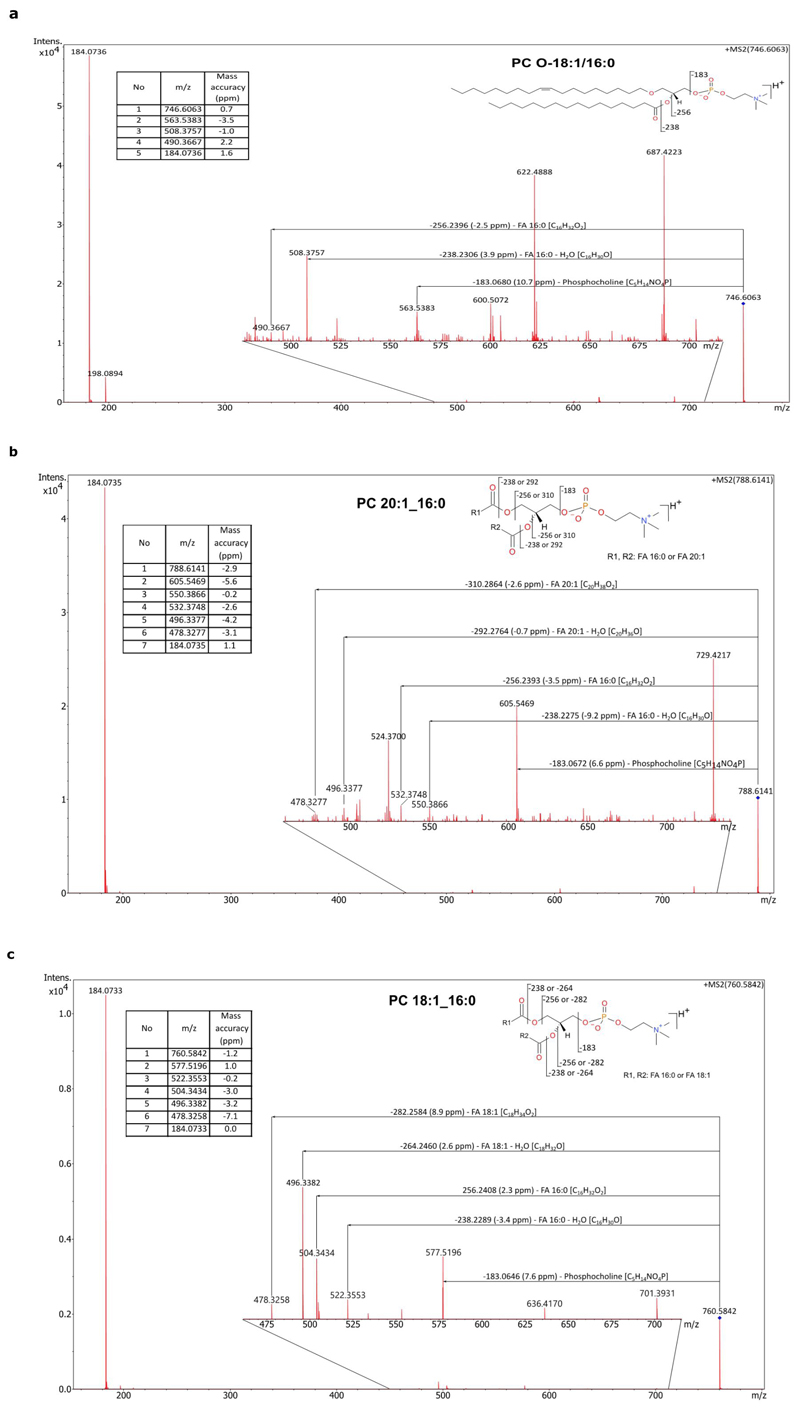
MS/MS validation of lipids detected in metastases by MALDI-MSI molecular imaging a. MS/MS spectrum of the ion at m/z 760.5842 detected from the metastatic areas within the lung tissue in positive ion mode using the timsTOF fleX. Ions supporting the assignment of PC O-18:1_16:0 are annotated with their corresponding mass accuracy. b. MS/MS spectrum of the ion at m/z 760.5842 detected from the metastatic areas within the lung tissue in positive ion mode using the timsTOF fleX. Ions supporting the assignment of PC 20:1_16:0 are annotated with their corresponding mass accuracy. c. MS/MS spectrum of the ion at m/z 760.5842 detected from the metastatic areas within the lung tissue in positive ion mode using the timsTOF fleX. Ions supporting the assignment of PC 18:1_16:0 are annotated with their corresponding mass accuracy.

4T1 cells were injected i.v. into healthy 6-8 weeks old female BALB/c mice. After 13 days, lung tissue was embedded in 3% carboxymethylcellulose (CMC), snap-frozen in a cryomold dipped in liquid-nitrogen-cooled isopentane (2-methylbutane, Sigma Aldrich) and stored at -80°C. Right before sectioning, the frozen tissue was placed at -20°C and 10 μm sections were cut using a Microm HM525 NX cryostat (Thermo ScientificTm), thaw-mounted onto conductive IntelliSlides (Bruker Daltonics, Bremen, Germany) and dried in a vacuum desiccator at room temperature for 30 minutes. For matrix coating, 7 mg/ml norharmane in 2:1 chloroform and methanol (v/v) was sonicated for 10 minutes and applied onto the slides using an HTX M5-SprayerTM (HTX technologies, LC, North Carolina, USA) at a flow rate of 0.125 mL/min, spray nozzle temperature of 30° C, and spray nozzle velocity of 1350 mm/min. A 16-pass cycle was used with 3 mm track spacing, and the nitrogen gas pressure was maintained at 10 psi. MALDI-MSI was performed on a timsTOF fleX MALDI-2 mass spectrometer (Bruker Daltonics, Bremen Germany) in positive mode within a m/z range of 250–1800, using a 50 μm by 50 μm raster size with 300 laser shots/pixel at a laser frequency of 10 kHz. External calibration was performed using red phosphorous dissolved in acetone and spotted beside the tissue section. Images were acquired using FlexImaging 7.0 software (Bruker Daltonics, Bremen, Germany). For m/z of interest, on-tissue MS/MS fragmentation spectra were acquired in positive ion mode using a precursor ion isolation width of 1 Da and collision energy of 35 eV. Data were analysed using SCiLS Lab 2023a software (Bruker Daltonics, Bremen, Germany) with a mass accuracy of ±10 ppm, without denoising and applying root mean square (RMS)-normalisation. Spectral data and regions of interest (ROI) defined in SCiLS were exported to MetaboScape 2021a (Bruker Daltonics, Bremen, Germany), and the T-ReX2 (MALDI-Imaging) algorithm was used for the extraction of features, de-isotoping, ion deconvolution, and annotation of lipids. A bucket table with total of 1000 speckles per ROI (full tissue section) was created by averaging the spectra within an area of the size of the width-by-height (6:3 ratio), resulting in 90% covered pixels. The following adducts were considered: H+, Na+, K+. The bucket table was annotated with the Metaboscape build-in lipid annotation tool, and a list of lipids obtained from the MS-DIAL’s LipidBlast database was used for this purpose. Annotation was performed with 5 ppm (narrow) or 10 ppm (wide) mass tolerance and a mSigma value below 250. Next, the compound annotation list was exported back to SCiLS Lab 2023a. To generate segmentation maps showing regions of spectral similarity, bisecting k-means clustering was applied to all individual peaks in the imported feature list using the correlation distance metric. MS/MS spectrums are shown in Extended Data Figure 10. After MSI analysis, the MALDI matrix was removed by submerging the slide in 100% methanol for 30 seconds, the tissue was stained with haematoxylin-eosin (H&E). The scanned optical images were imported in SCiLS and co-registered with the MALDI-MSI distribution images. The metastatic ROIs were defined based on both the segmentation map and the matched optical image of the same H&E tissue section. For feature annotations, the ROIs were exported to Metaboscape 2021a, and the same procedure as described above was followed. For the metastatic cluster, spatial m/z colocalized values were searched by calculating Pearson’s correlations between the spatial masks of the cluster and each m/z value and taking the m/z values with the highest correlation values (r > 0.5). Finally, annotations for m/z of interest were manually verified against the LipidMaps database based on the mass accuracy (<10 ppm) and characteristic ions observed in the fragmentation spectra (Bruker Compass DataAnalysis v6.0).

### Lung dissociation and flow cytometry analysis of cancer cell populations

Prior injections, 4T1, 4T07, and EMT6.5 cancer cells were transfected with the mammalian expression lentiviral vector pLKO.3 Thy1.1 (Addgene plasmid #14749) containing the surface protein Thy1.1 as a reporter protein. Since Thy1.1 is not usually expressed on mouse cells and does not elicit immune activation if injected in recipient mice, its overexpression served to identify cancer cells from single-cell suspension of mouse tissues. Lentiviral particles were produced in HEK293T cells and transduction of cancer cells was performed overnight. After 72 hours of recovering, CD90.1-positive cells were FACS-sorted. CD90.1-expressing 4T1 and EMT6.5 cells were then injected i.v. into 6-8 weeks old BALB/c mice. After 11 days, mice were anesthetized with Ketamine (100mg/kg) + Xylazne (10mg/kg) and lungs were perfused through the right ventricle (adapted from ^76^). Once lungs were extracted, the tissue was washed in blood bank saline, dry and minced for >2min using blades. Tissues were incubated with Liberase (Roche) (0.3 mg/mL) and DNAse1 (1 μg/mL) during 45 min at 37°C with occasional vortex. The reaction was quenched with 3% FBS:PBS + 2mM EDTA and filtered through a 70μm cell strainer. Cell pellet was washed, incubated with Red Blood Lysis buffer (Merck) and transferred through a 40 μm cell strainer. Single-cells suspension was counted and 30x10^6^ cells/mL, preincubated with anti-Mouse CD16/CD32 (Fc block, BD Biosciences), and stained for flow cytometry analysis. Antibodies against CD45 (BD Bioscience, 550994 1:250 dilution), PDPN (BioLegend, 127409 1:250 dilution) and CD90.1 (BioLegend, 202505 1:400 dilution) were used for selecting cancer cells from the single-cell suspensions of murine lung. For cell-surface antigen staining, the samples were incubated with antibodies for 20 min at 4°C, washed and resuspended in PBS containing 3% FBS:PBS +2 mM EDTA. To exclude dead cells, Viability efluor450 (ThermoFisher, 65-0863-14 1:500 dilution) was used. Single cells were analyzed using a BD FACSCanto II with FACSDiva (BD Biosciences). Metastatic burden was determined by assessing the fraction of CD90.1 positive cells in the lung.

### Primary alveolar type II cells isolation by flow cytometry and 3D culture

For *in vivo* analysis, primary AT2 cells (EpCam+MHCII+CD49flo) cells were isolated from control (healthy) or 4T1 and 4T07 tumor-bearing (primary tumor, day 17 post m.f. injection) BALB/c mice by flow cytometry sorting following the gating strategy previously reported ^77^. Briefly, mice were anesthetized with Ketamine (100mg/kg) + Xylazne (10mg/kg) and lungs were perfused and dissociated as described above. Single-cell suspensions were preincubated with anti-Mouse CD16/CD32 (Fc block, BD Biosciences), before a 30-min incubation with one or more fluorochrome-labeled antibodies: CD45 (BD Bioscience, 550994 1:200 dilution), CD31 (BD Bioscience, 562939 1:200 dilution), CD49f (Thermo Scientific, 25-0495-82 1:100 dilution), EpCAM (Thermo Scientific, 17-5791-82 1:200 dilution), MHC-II (BioLeged, 107643 1:100 dilution), and Viability efluor780 (ThermoFisher, 65-0865-14 1:800 dilution). A minimum of 1.2× 10^5^ AT2 cells (purity ≥92%) were sorted using FACSAria Fusion (BD Biosciences) and placed directly in TRIzol™ Reagent (Thermo Scientific). RNA extraction, cDNA synthesis and gene expression analysis by qPCR were performed as described above.

For *in vitro* experiments, primary AT2 cells were isolated from CreER induced lineage-tracing mice^78^. *Sftpc-CreERT2* mice were a gift from the J-H Lee laboratory (MRC Cambridge Stem Cell Institute). Breeding and all animal procedures at the Francis Crick Institute were performed in accordance with UK Home Office regulations under project license PPL80/2531. For type II cell lineage labeling, 3 doses of tamoxifen dissolved in Mazola corn oil (40 mg/mL stock solution) were given to *Sftpc-CreERT2;R26R-YFP* female C57BL/6J mice (8-10 weeks) via oral gavage over consecutive days (0.2 mg per g body weight). Lungs were harvested two weeks after the final dose. Lung tissues were dissociated as previously described ^18^. Briefly, lungs were minced manually and then digested in a mixture of DNase I (Merck Sigma-Aldrich) and Liberase TM and TH (Roche) in HBSS solution for 30 min in a 180rpm shaker at 37ºC. Samples were then washed, passed through a 100 μm filter, and centrifuged at 300 x g for 10 min. The resulting cell pellet was incubated in Red Blood Cell Lysis buffer (Miltenyi Biotec) for 4-5 min at room temperature. After a wash with MACS buffer (0.5% BSA and 250 mM EDTA in PBS), samples were passed through a 20μm strainer-capped flow cytometry tube to generate a single cell suspension. Cells were incubated with mouse FcR Blocking Reagent (Miltenyi Biotec) for 10 min at 4ºC followed by an incubation with CD45-BV421 (Biolegend 103133, clone 30-F11, 1:200 dilution) and Ter119-BV421 (Biolegend 116233, TER-119, 1:200 dilution) for 30 min at 4°C. Cells were washed twice with MACS buffer, before being incubated with 4′,6-diamidino-2-phenylindole (DAPI) to discriminate dead cells. AT2 cells (CD45-Ter119-YFP+) were sorted on a BD Influx cell sorter (BD Biosciences).

Isolated AT2 cells were seeded at a density of 150,000 cells/well in a collagen-coated Alvetex™ Scaffold 12-well plate insert (ReproCELL). Collagen solution was made by 30μg/mL PureCol collagen (Advanced Biomatrix), 0.1% bovine serum albumin, 20mM HEPES in HBSS. 2 mL of control or tumor-conditioned (generated in DMEM/F12 as described above) MEM medium (DMEM/F12) with 0.5% fetal bovine serum, 100 U/ml penicillin-streptomycin, 20 ng/ml EGF and 10 μg/ml insulin was added below the insert. Cells were cultured at 37ºC and 5% CO_2_ for 72h. Media was collected at experimental endpoint, centrifuged and filtered to remove any cellular debris, and stored at -80°C until further metabolomics analysis by mass spectrometry. For RNA extraction, scaffolds were washed and placed directly into QIAzol lysis reagent and incubated on a shaker at 100 rpm for 15 minutes at room temperature. RNA was then isolated using the RNeasy Mini kit (Qiagen), together with an on-column DNase treatment (Qiagen). cDNA was synthesized with random primers, using the SuperScript III First Strand Synthesis Kit (Thermo Fisher Scientific) according to the manufacturer’s protocol.

#### Single-cell RNA sequencing, data pre-processing, and cell-type assignment

Single-cell suspensions from the lungs of female BALB/c mice injected with control media (CM) or tumor conditioned media (TCM) for 3 weeks, or of mice fed with control diet (CD) or high fat diet (HFD) for 16 weeks, were prepared by following the protocol described above for lung dissociation. Cell suspensions were kept on ice and immediately processed for single-cell library preparation. Cell suspensions for each sample were converted to barcoded single-cell cDNA libraries using the Chromium Single Cell 5’ V1.1 Library (10x Genomics) kit, following the manufacturer’s guidelines, and aiming for a total of 10000 cells per library. Single-cell libraries were then sequenced on a NovaSeq 6000 System (Illumina). The sequenced reads were then mapped to the mouse genome (mm10 build GRCm38.p6) using the Cell Ranger software (10x Genomics), and the resulting single-cell gene expression data were analyzed within the *R/Bioconductor* framework. Specifically, the raw UMI count matrices for all samples in each individual experiment were first imported and merged using *Seurat* ^79^, and then converted for further processing with *Monocle* ^80^. Low-quality cells were then filtered based on standard quality-control metrics, with sample-specific thresholds chosen based on evaluating quality-control histograms for each experiment independently. In particular, cells were filtered based on their mitochondrial RNA content (allowing for a maximum of 10% in the case of CM/TCM, or 20% in the case of CD/HFD), library size (removing cells with total UMI counts below 500 in both cases and number of detected genes (removing cells expressing less than 200 genes in the case of CM/TCM, or 250 in the case of CD/HFD). Genes expressed in less than 5 cells were additionally ignored in all subsequent analyses. Size-factor and variance-stabilizing normalization (based on fitting to a negative binomial distribution) were then applied to the filtered data sets, and highly variable genes (HVGs) were identified for each of them based on their departure from the average normalized dispersion *versus* expression trend observed among all genes. After excluding mitochondrial, ribosomal-protein, and cell cycle-associated genes, the top 1000 HVGs with size-factor normalized expression levels above 0.01 were selected. Principal component analysis (PCA) was then performed on the size factor-normalized and variance-stabilized count matrix restricted to these genes only, followed by 2D UMAP dimensional reduction ^80^ based on the resulting top 50 principal components (with *correlation* distance metric, *number of neighbors* = 15, and *minimum distance* = 0.1, and without further PCA scaling). After that, cells were clustered in the UMAP plane by applying the *Louvain* ^80,81^ graph-based algorithm at high resolution (resolution = 0.001 with *k_NN_* = 10 for the CM/TCM and *k_NN_* = 15 for CD/HFD), in order to attain a fine-grained cluster structure for each data set (158 clusters for CM/TCM and 51 for CD/HFD). The resulting fine-grained clusters were then manually annotated to specific cell types, based on evaluating the cluster-averaged normalized expression profiles of several cell type-specific markers. For marker score analysis, cell-type marker scores were calculated using the *GSVA* (gene set variation analysis) package ^82^. In brief, count matrices for the filtered data sets were first subject to size-factor and variance-stabilizing normalization. *GSVA*-based z-scores were then determined, for every cell, for a manually assembled list of marker gene sets and further scaledto the range 0–1, by means of the following mapping:

$${\text{X}}_{\text{M},\text{C}}=\frac{{\text{Z}}_{\text{M},\text{C}}-Z_{\text{M},\min}}{Z_{\text{M},\max}-{\text{Z}}_{\text{M},\min}}$$

where ${\bar{X}}_{\text{M},\text{C}}$ is the scaled (0–1) score for a given marker set, M ande cell C, while Z_M,C_ is the az-score for that cell and marker set, and Z_M,max_ and Z_M,min_ are respectively the highest and lowest values of *Z*_M,C_ among all cells (for each marker set M).

### Lipidomics analysis

7-8 weeks old female BALB/c mice were inoculated with 4T1 cells in the mammary fat pad (1 × 10^6^ cells). After 23 days, primary tumors and lung metastases were collected and snap frozen. Primary tumor and lung metastatic tissues (n=6) were pulverized (Cryomill, Retsch) under liquid-nitrogen conditions and lipids were extracted by mixing 700 μL in water, homogenizing (Precellys, Bertin) with 800 μL 1 N HCl:CH3OH 1:8 (v/v), 900 μL CHCI3, 200 mg/mL of the antioxidant 2,6-di-tert-butyl 4-methylphenol (BHT; Sigma-Aldrich) and 3 μL of SPLASH LIPIDOMIX Mass Spec Standard (#330707, Avanti Polar Lipids). After vortexing and centrifugation, the lower organic fraction was collected and evaporated in a speedvac at room temperature. The remaining lipid pellet was stored at -20C under argon and subsequently reconstituted in 100% ethanol just before mass spectrometry analysis. Lipid species were analyzed by liquid chromatography electrospray ionization tandem mass spectrometry (LC-ESI/MS/MS) as previously described ^23^ on a Nexera X2 UHPLC system (Shimadzu) coupled with hybrid triple quadrupole/linear ion trap mass spectrometer (6500þ QTRAP system; AB SCIEX). Peak integration was performed with the MultiQuantTM software version 3.0.3. Lipid species signals were corrected for isotopic contributions calculated using Python Molmass 2019.1.1 and were quantified based on internal standard signals and adheres to the guidelines of the Lipidomics Standards Initiative (LSI) (level 2 type quantification as defined by the LSI).

### Patient selection and sample collection

An informed consent was obtained from all participants included in this study. For collection of human samples for interstitial fluid measurements, the study was approved by the local ethics committee (Medical EthicsCommittee UZ/KU Leuven, protocol S57123) as described in^18^. Briefly, human ‘normal’ lung tissue was collected from patients who underwent lung surgery for emphysematous lung volume reduction or tumorectomy. In the latter case, lung tissue from the resection specimen was taken as far away as possible from the tumor front (Supplementary Table 1). Following surgical resection, lung samples were taken and transported to our research facility for interstitial fluid extraction.

In addition, this study has benefited from two different clinical programs developed at the UZ Leuven hospital (Belgium).

#### UPTIDER program

Snap frozen and/or fresh tissue samples from metastases as well as normal tissues were obtained through the ethically approved UPTIDER program (UZ/KU Leuven Program for Post-mortem Tissue Donation to Enhance Research, NCT04531696, S64410). The UPTIDER project is coordinated and performed by the Laboratory for Translational Breast Cancer Research (LTBCR) at our institution, KU Leuven, under the lead of Prof. Christine Desmedt and Prof. Dr. Giuseppe Floris. In this project, patients with metastatic breast cancer that consent to participate undergo a rapid research autopsy in the first 12 hours after death. In short, upon death of a participating patient, the body is transported to the morgue of the UZ Leuven hospital. Several types of body fluids are collected, as well as extensive malignant and adjacent normal tissue samples from different organs in the body, including the ones that are difficult to reach during the life of the patient. Tissue samples are processed in different conditions (snap-frozen in liquid nitrogen, frozen in OCT (Optimal Cutting Temperature Compound), collected in neutral buffered formalin for further processing in formalin-fixed paraffin embedded blocks (FFPE), or fresh in specific media for in vivo development). For our experiments described in this paper, fresh samples were collected, part was used to freshly isolated interstitial fluid, and others snap-frozen in liquid nitrogen. Proteins were extracted from snap-frozen tissues following the cell-fractionation and nuclear isolation protocol described above. CPT1a and KAT2a expression was assesed in the cytosolic and nuclear fraction respectively. Clinicopathological information for every patient in the UPTIDER data set is shown in Supplementary Table 2.

#### CHEMOREL program

Patients for the retrospective translational CHEMOREL study were selected from a clinical-pathological database in which all breast cancer patients, who were diagnosed and treated at the UZ Leuven Multidisciplinary Breast Center, are documented (i.e. patient and tumor characteristics, therapy, and follow-up information including relapse and survival). From this database, a homogeneous cohort of newly diagnosed primary Luminal B-type breast cancer patients with grade 2/3, ER+, PR+/-, HER2- tumors was selected including (i) patients who remained disease-free for at least 6 to 10 years after the initial therapy (n=43), (ii) patients developing distant metastasis within 5 years after initial therapy (n=44), and (iii) primary metastasized patients (n=14). Groups were matched for patient age, tumor 18, and node status. Clinicopathological information for patients in the CHEMOREL data set is shown in Supplementary Table 3.

### RNA sequencing analysis

#### Patient tumors

RNA was extracted from 5x10μm FFPE unstained tissue slides prepared from the left-over of surgical resection specimens after standard pathological diagnostic procedures. An extra tissue slide was cut sequentially with the unstained slides and was H&E stained and microscopically inspected by an expert breast pathologist (Prof. Dr. G. Floris, UZ Leuven) to ensure representative and comparable tumor cellular composition across the entire cohort. RNA extraction was performed by using the HighPure FFPET RNA extraction kit (Roche). RNA concentration and quality were assessed on the Agilent Bioanalyzer. RNA sequencing workflow and subsequent bioinformatics were accomplished at the Laboratory of Translational Genetics (VIB-KU Leuven, Prof. D. Lambrechts). RNA libraries were created using the KAPA stranded mRNA seq Library preparation kit according to the manufacturer’s instructions. Briefly, poly-A containing mRNA was purified from total RNA using oligo(dT) magnetic beads and fragmented into 200–500 bp pieces using divalent cations at 94ºC for 8 min. The cleaved RNA fragments were copied into first-strand cDNA. After second-strand cDNA synthesis, fragments were A-tailed and indexed adapters were ligated. The products were purified and enriched by PCR to create the final cDNA library. After confirmation of successful library construction, the resulting libraries were sequenced on a HiSeq2500 or HiSEq4000 (Illumina) using a V3 flowcell generating 1 x 50 bp reads, yielding reliable results for 43, and 14 patients from groups I and ii, respectively. Optical duplicates and adapator sequences were removed from the raw sequencing reads before aligning to the transcriptome and the reference genome using TopHat 2.0 ^83^ and Bowtie 2.0 ^84^. Counts were assigned to genes using the HTSeq software package. Raw sequencing reads were mapped to the transcriptome: > 25000 different transcripts were identified that could be detected in at least 50% of the samples. For the purpose of this study, the groups of primary metastasized patients (n=14) (iii) and patients who remained disease-free for at least 6 to 10 years after the initial therapy (n=43) (i) were used for differential gene expression analysis.

#### 2D and 3D spheroid cultured cells

RNA from freshly collected cells was extracted using TRIzol™ Reagent (Thermo Scientific). RNA integrity and concentrations were measured on the Agilent Bioanalyze, and libraries were prepared using KAPA Stranded mRNA Sequencing Kit (Roche) according to the manufacturer’s instructions and as described above. After quantification with qPCR, the resulting libraries were sequenced on a HiSeq4000 (Illumina) using a flow cell generating 1x50bp single-end reads. The resulting reads were cleaned with the fastq-mcf software, after a quality control was performed with FastQC (v0.11.9). The high-quality reads were then mapped to the *Mus Musculus* reference genome (GRCm38/mm10) with HISAT2 (v2.1.0) and the abundance of reads per gene was determined with HTSeq-count. Differential gene expression analysis was performed with the R package DESeq2 (v1.22.0). To further investigate the significantly differential genes, genes were ranked according to the score-sign(log2FC)*log(padj), and used as input for a geneset enrichment analysis using GSEA software of the Broad Institute. In the pre-ranked analysis, only gene sets containing between 15 and 1000 genes were retained and the number of permutations was set at 1000. All pathways with GSEA FDR < 0.05 were considered significant. Pathway comparison and upstream regulators analysis were done in Ingenuity Pathway Analysis. The threshold criteria of P-value ≤ 0.05 and a fold change of ≥0.5 in gene expression among the different pairwise comparisons were used.

### In silico gene expression analysis and pathway analysis

Transcript mRNA expression data were collected from patients with primary breast cancer from TCGA (n=1,221) and METABRIC (n= 1,904). Datasets were downloaded from The Cancer Genome Atlas (TCGA) portal (http://tumorsurvival.org/download.html), and cBioportal (https://www.cbioportal.org/datasets). These data were gathered from publicly available datasets and did not require institutional review board approval or patient informed consent. All patients with mRNAseq and clinical data were included. Clinical parameters, including survival information, tumor stage, tumor subtype, were acquired from TCGA’s Clinical Data Resource data set (https://gdc.cancer.gov/about-data/publications/PanCan-Clinical-2018). We used TNM staging according to the AJCC Cancer Staging Manual, 7th edition, to maintain consistency for all patients from TCGA and METABRIC datasets. Clinical information of those two datasets is shown in Supplementary Table 4. For statistical analysis, the Kaplan–Meier method was used to analyze the relationship between gene expression (CPT1A-low and CPT1A-high, median as cutoff) and survival prognosis. Univariate and multivariate Cox regressions were used to analyze the associations between patient clinical information (age, TNM stage, and tumor subtype (classified by PAM50)) and overall survival. R software (version 4.0.5 (R Project for Statistical Computing)) was used to perform KM survival analysis, univariate and multivariate Cox regressions by using a p-value < 0.05 as the filter value.

The expression levels of *CPT1A* in human tissues were analyzed in the Genevestigator database, using the HS00002-(33) public microarray and RNA-Seq study results^85^. Expression values are shown as fold change of normalized expression values calculated by Genevestigator calculated using standard normalization methods for different microarray platforms.

Gene set enrichment analysis (GSEA): To compare gene expression profiles from breast cancer metastases at different organ sites, the data set GSE14018 was downloaded from Gene Expression Omnibus website (GEO, https://www.ncbi.nlm.nih.gov/geo/query/acc.cgi?acc=GSE14018) ^86^. GSEA was performed using the NF-kB-related transcriptional signature HALLMARK_TNFA_SIGNALING_VIA_NFKB from the Molecular Signature Database (MsigDB) ^87^. Single sample gene set enrichment analysis (ssGSEA) was performed using GenePatterns (https://www.genepattern.org/).

Pathway analysis: QIAGEN’s Ingenuity® Pathway Analysis (IPA®, QIAGEN) tool was applied to differential gene expression data obtained from the RNA sequencing, The activation score was calculated based on the direction of change (i.e. expression in the sg*CPT1A* + acetate relative to control sh*CPT1A* without acetate. The threshold criteria of P-value ≤ 0.05 and a fold change of ≥0.5 were used.

### Statistics and reproducibility

Statistical data analysis was performed using GraphPad Prism 8 (GraphPad Software) on n≥ 3 biological replicates. Data are presented as mean ± s.e.m. Details of statistical tests and post-tests are presented in the figure legends. P values lower than 10^-15^ are indicated as p<0.0001. Determination of mathematical outliers was performed using ROUT method of regression (Prism) with coefficient Q = 1%.. Sample size for all *in vitro* experiments was chosen empirically or based on previous similar studies that have given statistically significant results. For in vivo experiments, sample size was determined using power calculations with β = 0.8 and P < 0.05, based on preliminary data.

For *in vivo* experiments, mice were randomized before diet feeding, tumor conditioned/control media injections, or injection with the different cell lines. In addition, mice were given a unique number prior to data collection for bliding analysis. For *in vitro* studies, samples were randomized, when possible, prior data acquisition.

### Reporting summary

Further information on research design is available in the Nature Research Reporting Summary linked to this article.

## Extended Data

## Supplementary Material

Dataset Extended Figure 1

Dataset Extended Figure 3

Dataset Extended Figure 4

Dataset Extended Figure 5

Dataset Extended Figure 6

Dataset Extended Figure 7

Dataset Extended Figure 8

Dataset Extended Figure 9

Dataset Figure 1

Dataset Figure 2

Dataset Figure 3

Dataset Figure 4

Dataset Figure 5

Dataset Figure 6

Dataset Figure 7

Uncut western blots Extended Figure 4

Uncut western blots Extended Figure 8

Uncut western blots Extended Figure 9

Uncut western blots Figure 3

Uncut western blots Figure 6

Uncut western blots Figure 7

Supplementary Tables

## Figures and Tables

**Table 1:** Primers used to analyze mRNA levels

Target organism	Target gene	Sequence 5’ – 3’
Human	*RPL19*	Fwd: ATTGGTCTCATTGGGGTCTAAC
		Rev: AGTATGCTCAGGCTTCAGAAGA
Human	*CPT1A*	Fwd: GCACCTCCGTAGCTGACTC
		Rev: GAGTGACCGTGAACTGAAAGG
Human	*ICAM1*	Fwd: GGCTGGAGCTGTTTGAGAAC
		Rev: CTGTGGGGTTCAACCTCTG
Human	*PTPN13*	Fwd: TTCTCTGCAGACCTCCACCT
		Rev: TCTTCTCCACTCCCACTGCT
Human	*TNFAIP2*	Fwd: GAAGTCTGGCTGAGGTCTGG
		Rev: CTCCAGAAGGAGTGCAGGAC
Human	*MMP9*	Fwd: TTGACAGCGACAAGAAGTGG
		Rev: GCCATTCACGTCGTCCTTAT
Human	*JAG1*	Fwd: TGCTACAACCGTGCCAGTGACT
		Rev: TCAGGTGTGTCGTTGGAAGCCA
Mouse	*Cpt1a*	Fwd: CAGAGGATGGACACTGTAAAGG
		Rev: AGTATGCTCAGGCTTCAGAAGA
Mouse	*Rpl19*	Fwd: CAGGCATATGGGCATAGGGAA
		Rev: TGCCTTCAGCTTGTGGATGT
Mouse	*Icam1*	Fwd: AGCACCTCCCCACCTACTTT
		Rev: AGCTTGCACGACCCTTCTAA
Mouse	*Ptpn13*	Fwd: GAACACCTCGACTGTGCTGA
		Rev: GGACGCTGGTATTCACACCT
Mouse	*Tnfaip2*	Fwd: AAAAAGGACCAGCCCAGATT
		Rev: TACAGAGCCTCCACCTTGCT
Mouse	*Mmp9*	Fwd: TGAATCAGCTGGCTTTTGTG
		Rev: GTGGATAGCTCGGTGGTGTT
Mouse	*Fabp5*	Fwd: GACGACTGTGTTCTCTTGTAACC
		Rev: TGTTATCGTGCTCTCCTTCCCG
Mouse	*Sfpb*	Fwd: TGTGCCAAGAGTGTGAGGAT
		Rev: CAGGGGCAGGTAGACATCAA
Mouse	*Sfpc*	Fwd: GATGAGAAGGCGTTTGAGGT
		Rev: GATGAGAAGGCGTTTGAGGT
Mouse	*Abca3*	Fwd: GGTCCTGATGGAGAGTCCAC
		Rev: GGAGCAGGAACGCTGAGAT
Mouse	*Acsl4*	Fwd: CCTTTGGCTCATGTGCTGGAAC
		Rev: GCCATAAGTGTGGGTTTCAGTAC
Mouse	*Kat2a*	Fwd: CACGGAAATCGTCTTCTGTGCC
		Rev: CGTACTCGTCAGCATAGGTGAG
Mouse	*Kat2b*	Fwd: CCTCTTCACCTGCGTCCACAAA
		Rev: TCTCCAAGGAGCCTTCAACCAC
Mouse	*Noa1*	Fwd: GAGCGGCATAAAATTCTGCACCG
		Rev: CACTGACACAGCAGTAGAGGCT
Mouse	*Kat5*	Fwd: TGAGCGTGAAGGACATCAGTGG
		Rev: TTAAGTCCAGCCGCTCGTGAGT
Mouse	*Kat6a*	Fwd: CGGTCAAACTCGCCACCAATTC
		Rev: CTAACACCTCCGTGGTCTCAGA
Mouse	*Kat6b*	Fwd: GCTGTGGTTTCTGAGGAAGAGC
		Rev: TGCCTACTGCTAACTCTGGACG
Mouse	*Ep300*	Fwd: GTGATGACCCTTCCCAACCTCA
		Rev: CTCGTGGTGAAGGACACAGATC
Mouse	*Crebbp*	Fwd: CACCATCTGTGGCTACTCCTCA
		Rev: GGTTTCAGCACTGGTCACAGAG
Mouse	*Kat7*	Fwd: AGGAAAAGGTGGCTGAACTCAGG
		Rev: GTCAGGTTTTCCAAGAGAGGCTC
Mouse	*Kat8*	Fwd: CAGCAGAAGTGATCCAGTCTCG
		Rev: TTGGTCAGTGCGAGTCGGTTCT

## Data Availability

Mouse scRNA-sequencing, RNA-sequencing, and ChIP-sequencing data have been deposited in the Gene Expression Omnibus (GEO) under accession code GSE196993. Source data for *in vivo* data represented in Figs. 1–7 and Extended Data Figs. 1–10 have been provided as Source Data files. Gel source images are available in Supplementary Fig. 1. All other data supporting the findings of this study are available within the Article and the Supplementary Information, and from the corresponding author on reasonable request..
