# Supplementary figures and images for: A palmitate-rich metastatic niche enables metastasis growth via p65 acetylation resulting in pro-metastatic NF-κB signaling
Source: Nat Cancer. Author manuscript; Available in PMC 2023 Oct 21. (PMC7615234; doi:10.1038/s43018-023-00513-2)

Imaging Source Data Extended Data Figure 4d

CPT1a

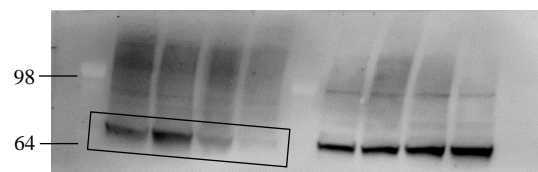

ACTIN

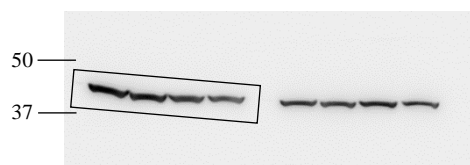

Supplement: Uncut western blots Extended Figure 4 [file EMS172138-supplement-Uncut_western_blots_Extended_Figure_4.pdf]

Imaging Source Data Extended Data Figure 8

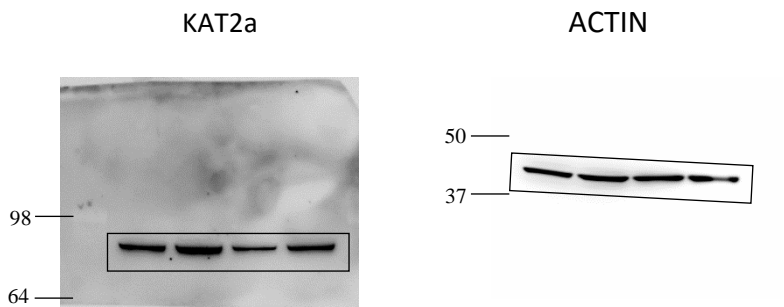

Supplement: Uncut western blots Extended Figure 8 [file EMS172138-supplement-Uncut_western_blots_Extended_Figure_8.pdf]

Imaging Source Data Extended Data Figure 9

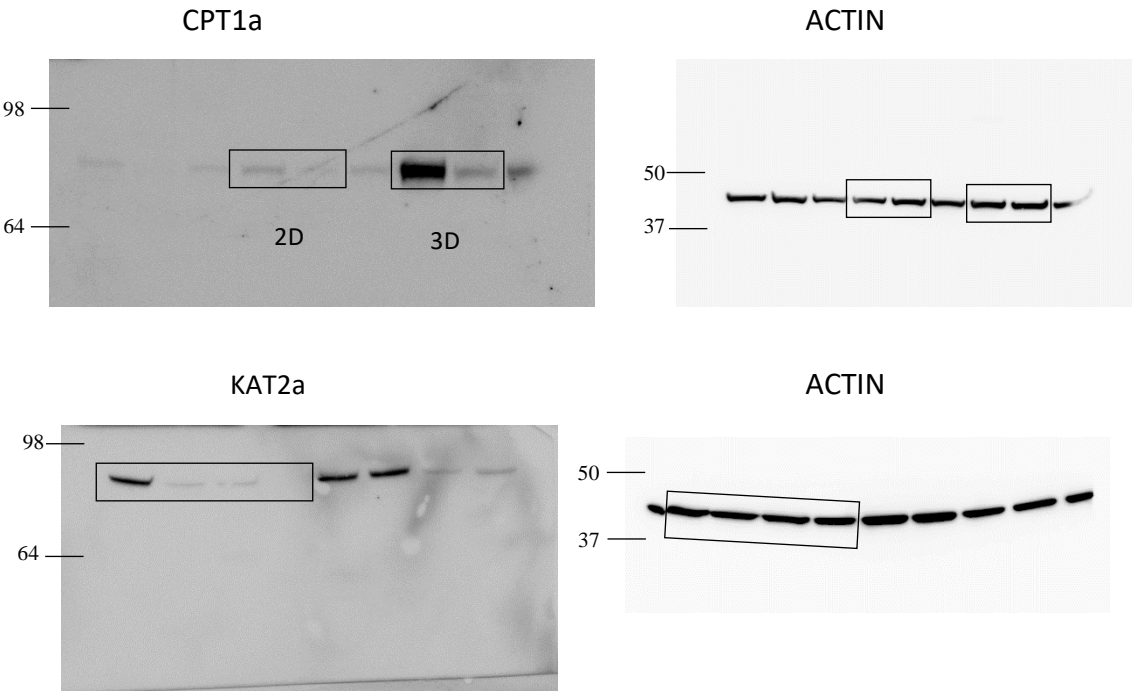

Supplement: Uncut western blots Extended Figure 9 [file EMS172138-supplement-Uncut_western_blots_Extended_Figure_9.pdf]

Imaging Source Data Figure 3e

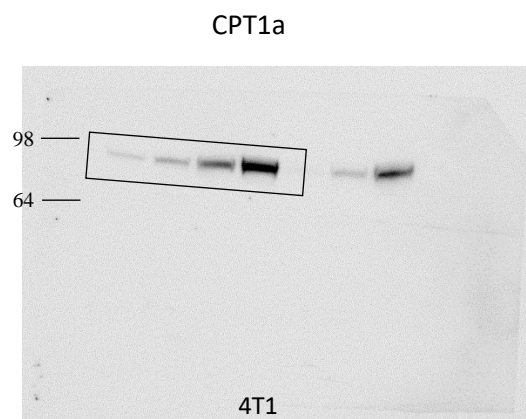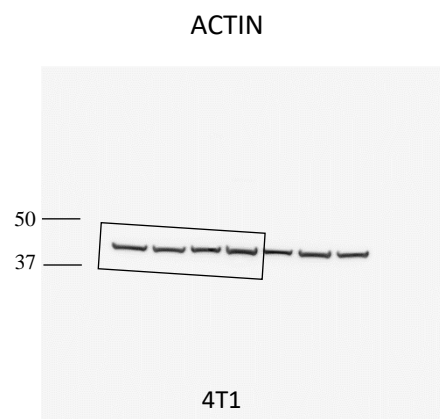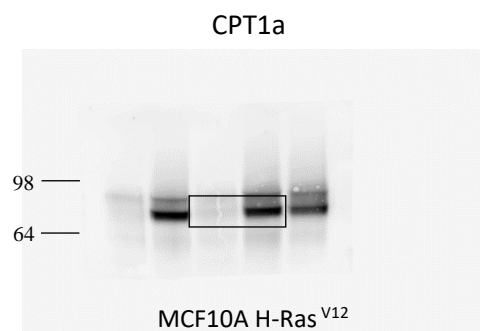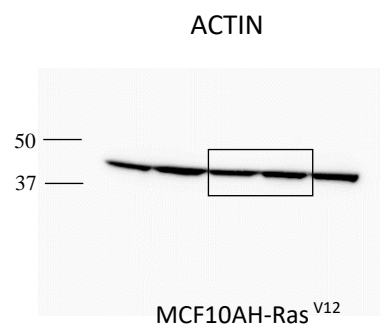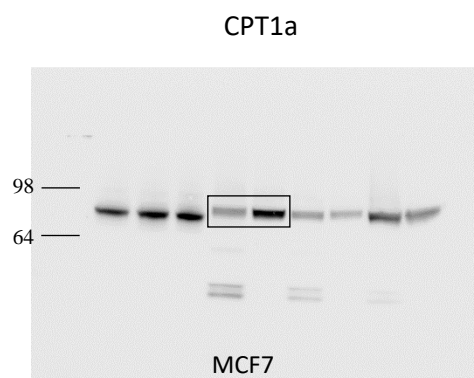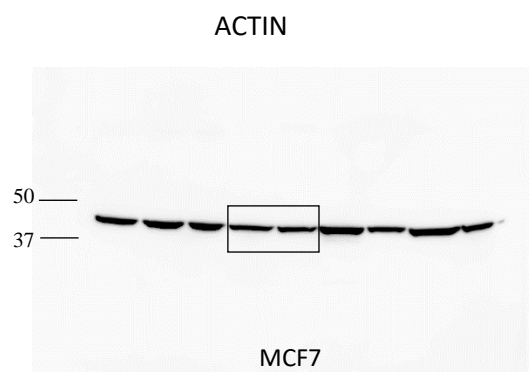

Supplement: Uncut western blots Figure 3 [file EMS172138-supplement-Uncut_western_blots_Figure_3.pdf]

Imaging source Data Figure 6e

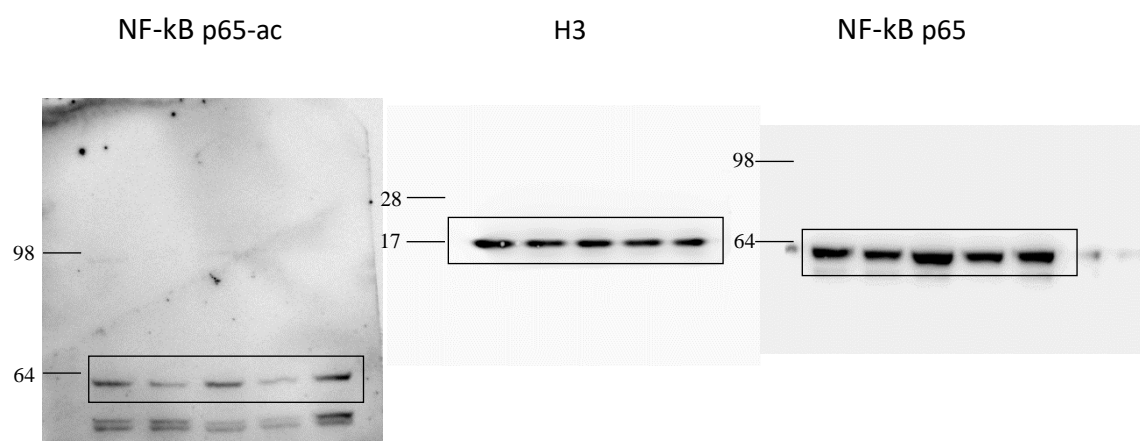

Supplement: Uncut western blots Figure 6 [file EMS172138-supplement-Uncut_western_blots_Figure_6.pdf]

Imaging Source Data Figure 7c

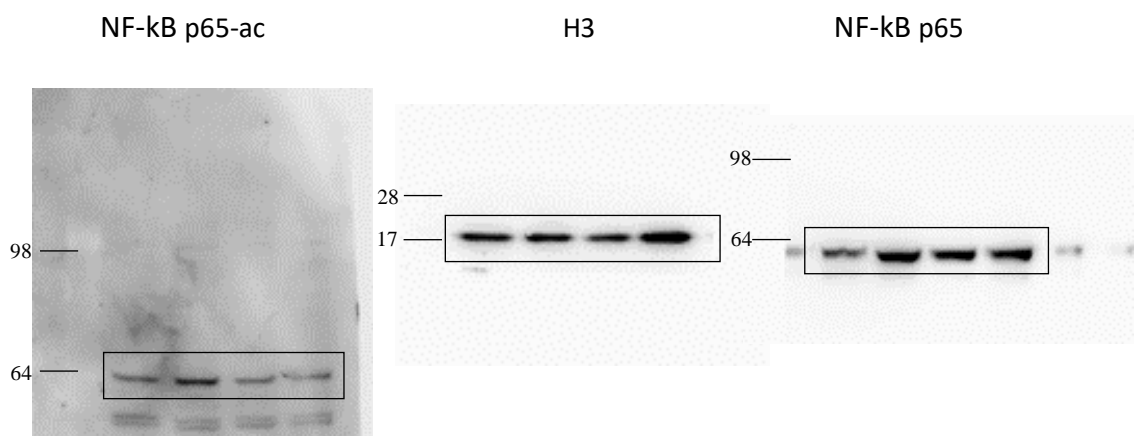

Imaging Source Data Figure 7g

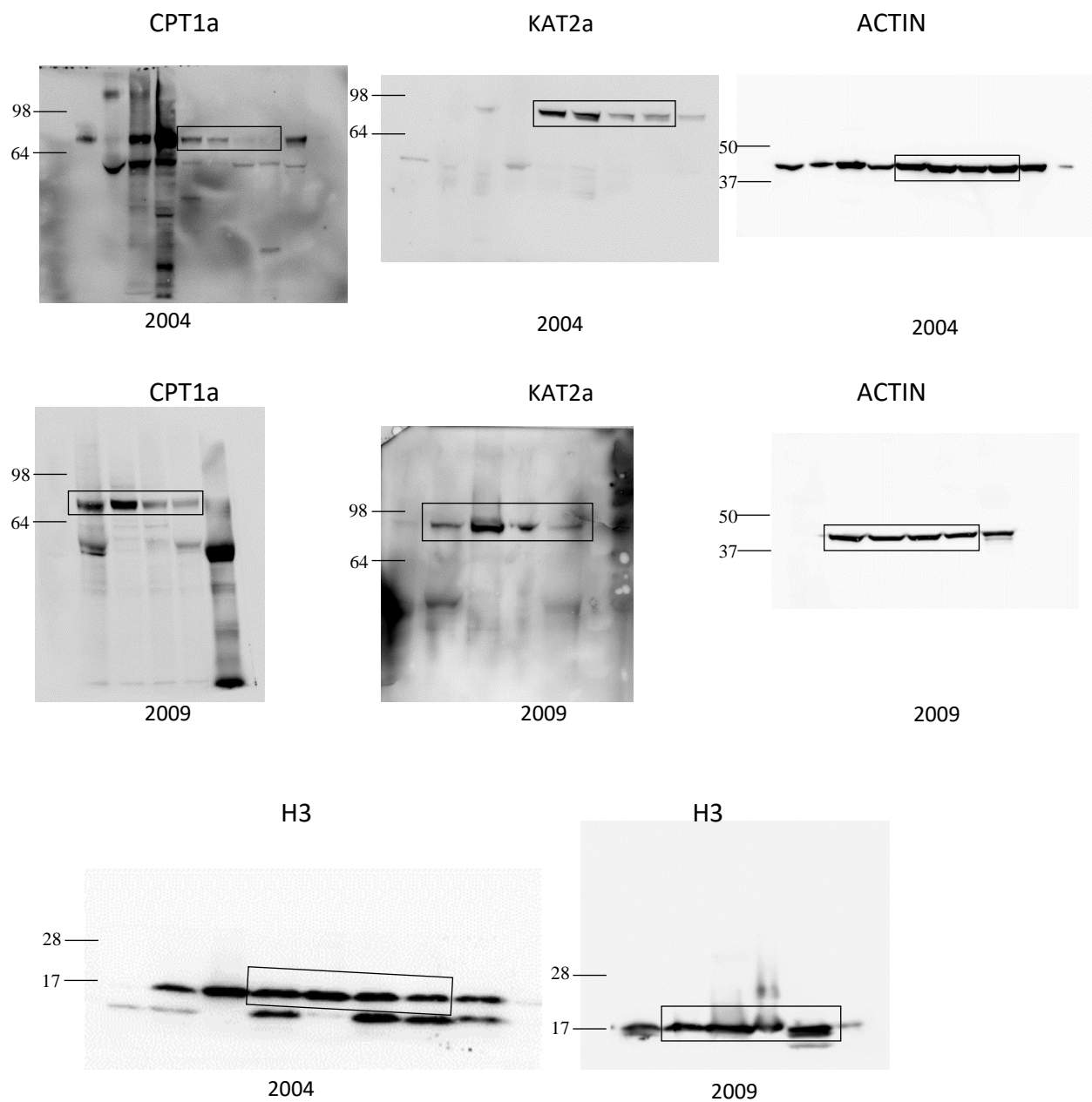

Supplement: Uncut western blots Figure 7 [file EMS172138-supplement-Uncut_western_blots_Figure_7.pdf]
