# Supplementary material for: A palmitate-rich metastatic niche enables metastasis growth via p65 acetylation resulting in pro-metastatic NF-κB signaling
Source: Nat Cancer. Author manuscript; Available in PMC 2023 Oct 21. (PMC7615234; doi:10.1038/s43018-023-00513-2)
Supplement: Supplementary Tables [file EMS172138-supplement-Supplementary_Tables.pdf]

SUPPLEMENTARY TABLE 1

Clinical data of patients from protocol S57123 (UZ Leuven)

Patient ID	Age diagnosis	Gender	Reason of surgery
S57123-001	68	M	volume reduction surgery in emphysema
S57123-002	69	M	volume reduction surgery in emphysema
S57123-003	58	M	peritumoral normal lung tissue from tumorectomy
S57123-004	64	F	volume reduction surgery in emphysema
S57123-005	56	F	peritumoral normal lung tissue from tumorectomy
S57123-006	34	F	peritumoral normal lung tissue from tumorectomy
S57123-007	66	M	peritumoral normal lung tissue from tumorectomy

SUPPLEMENTARY TABLE 2

Clinical data of patients from the UPTIDER program

Patient ID	Age diagnosis	Age death	Molecular subtype primary tumor	Histotype	Grade of tumor at diagnosis*	Stage at diagnosis*
2004	56	61	ER+/PR+/HER2-	Metaplastic-SCC	2	II
2005	53	60	ER+/PR-/HER2-	ILC	2	III
2006	46	60	ER+/PR+/HER2-	NST+ILC	2	I
2008	69	77	ER+/PR+/HER2-	NST	3	II
2009	51	62	ER+/PR+/HER2-	NST	3	II
2010	65	88	ER+/PR+/HER2-	NST	2	II
2011	51	60	ER+/PR+/HER2-	ILC	2	III
2016	49	56	ER+/PR+/HER2-	NST	2	I
2018	47	60	ER+/PR+/HER2-	NST+ILC	2	II

*When the samples were obtained they were both stage IV (which is the case for all UPTIDER patients)

SUPPLEMENTARY TABLE 3

Clinical data comparison between primary metastasized and non-metastasized patients in the CHEMOREL study.

Variables	Contrast	Primary metastasized patients	Non-metastasized patients
Age patients			
	N	14	43
	Median	63.5	54
	Average	62	56
	Range	[32.0; 88.0]	[36.0; 82.0]
Grade of tumor			
Grade 2	n/N (%)	0/14 (0%)	1/43 (2%)
Grade 3	n/N (%)	14/14 (100%)	42/43 (98%)
Stage (without M* info)			
Stage I	n/N (%)	0/14 (0%)	3/43 (7%)
Stage IIA	n/N (%)	0/14 (0%)	15/43 (35%)
Stage IIB	n/N (%)	5/14 (36%)	8/43 (19%)
Stage IIIA	n/N (%)	1/14 (7%)	14/43 (32%)
Stage IIIB	n/N (%)	5/14 (36%)	0/43 (0%)
Stage IIIC	n/N (%)	3/14 (21%)	3/43 (7%)
ER			
Positive	n/N (%)	14/14 (100%)	42/43 (98%)
Negative	n/N (%)	0/14 (0%)	1/43 (2%)
PR			
Positive	n/N (%)	13/14 (93%)	41/43 (95%)
Negative	n/N (%)	1/14 (7%)	2/43 (5%)
HER2			
Positive	n/N (%)	0/14 (0%)	0/43 (0%)
Negative	n/N (%)	14/14 (100%)	43/43 (100%)

Abbreviations: M*: Metastasis

SUPPLEMENTARY TABLE 4

Clinical information

Chracteristics		TCGA(n=1220) CPT1A-low (n=610)	TCGA(n=1220) CPT1A-high (n=611)	TCGA p value	METABRIC(n=1904) CPT1A-low (n=716)	METABRIC(n=1904) CPT1A-high (n=683)	METABRIC p value
		N(%)					
Age	median(ra	57(26, 90)	59(27, 90)	0,04	59.8(21.93, 96	3(26.72, 8	0,001
Age-group	young(<50	190	154	0,03	183	132	0,006
	old(>=50)	420	456		533	551	
Menopaus	pre	135	124	0,89	183	132	0,006
	post	379	389		533	551	
	intermedia	25	32		0	0	
	missing	71	66		0	0	
TNM-Stag	1	116	86	p<0.001	280	195	p<0.001
	2	358	339		380	420	
	3	115	161		52	63	
	4	8	14		4	5	
	missing	13	11		0	0	
Stage-grou	stage 1-2	474	425	p<0.001	660	615	0,19
	stage 3-4	123	175		56	68	
Subtype	LumA	231	249	0,14	307	213	p<0.001
	LumB	44	153		129	210	
	Her2-enric	31	43		42	100	
	Basal	116	41		83	52	
	normal	8	19		61	39	
	missing	180	106		94	69	
histologic :	IDC	438	448	0,41	546	545	0,05
	ILC	102	110		41	59	
	others	70	53		129	79	

Uni-multi variate analysis

KM plot and forest plot

cutoff: age(<50,>=50), stage(1-2 vs 3-4), subtype(LumA, LumB, Her2, Basal, Normal)

TCGA

category

variable	HR	95%CI	p-value	n	groups
**CPT1A**	**1,74**	**1.29-2.29**	**0,0002**	**1218(200)**	**median**
age	1,47	1.07-2.01	0,0200	1218(200)	<50 vs. >=50
stage	1,85	1.34-2.55	0,0002	1218(200)	1-2 vs. 3-4
subtype	0,79	0.34-1.81	0,5750	934(164)	LumA vs. Normal
	1,33	0.56-3.16	0,5116		LumB vs. Normal
	2,18	0.87-5.44	0,0949		Her2 vs. Normal
	0,97	0.40-2.33	0,937		Basal vs. Normal

*univariate analysis, model, OS=CPT1A/age/stage/subtype

variable	HR	95%CI	p-value	n	groups
**CPT1A**	**1,73**	**1.20-2.51**	**0,004**	**915(156)**	**median**
age	1,70	1.17-2.47	0,0050		<50 vs. >=50
stage	2,48	1.77-3.49	<0.001		1-2 vs. 3-4
subtype	0,72	0.31-1.67	0,4400		LumA vs. Normal
	0,90	0.38-2.17	0,8200		LumB vs. Normal
	1,97	0.78-4.96	0,1500		Her2 vs. Normal
	1,29	0.53-3.19	0,57		Basal vs. Normal

*multivariate analysis: model, OS=CPT1A+age+stage+subtype

TCGA

continuous

variable	HR	95%CI	p-value	n	groups
**CPT1A**	**1,01**	**1.00-1.02**	**0,0087**	**1218(200)**	**continuous**
age	1,47	1.07-2.01	0,0200	1218(200)	<50 vs. >=50
stage	1,93	1.58-2.34	<.0001	1196(188)	1,2,3,4
subtype	0,79	0.34-1.81	0,5800	934(164)	LumA vs. Normal
	1,33	0.56-3.16	0,51		LumB vs. Normal
	2,18	0.87-5.44	0,09		Her2 vs. Normal
	0,96	0.40-2.33	0,94		Basal vs. Normal

*univariate analysis

variable	HR	95%CI	p-value	n	groups
**CPT1A**	**1,01**	**1.00-1.02**	**0,025**	**915(156)**	**continuous**
age	1,68	1.02-1.05	0,0056		<50 vs. >=50
stage	2,02	1.68-2.58	<0.001		1,2,3,4
subtype	0,67	0.28-1.51	0,3508		LumA vs. Normal
	0,82	0.34-1.98	0,7016		LumB vs. Normal
	1,74	0.74-4.67	0,2402		Her2 vs. Normal
	1,12	0.46-2.74	0,7997		Basal vs. Normal

*multivariate analysis: model, OS=CPT1A+age+stage+subtype

METABRIC

category

variable	HR	95%CI	p-value	n	groups
**CPT1A**	**1,48**	**1.31-1.67**	**<.0001**	**1904(1103**	**median**
age	1,68	1.43-1.98	<.0001	1904(1103	<50 vs. >=50
stage	2,30	184-2.89	<.0001	1390(783)	1-2 vs. 3-4
subtype	0,86	0.67-1.11	0,2444	1699(1009	LumA vs. Normal
	1,30	1.01-1.67	0,0396		LumB vs. Normal
	1,47	1.12-1.94	0,0059		Her2 vs. Normal
	1,16	0.87-1.56	0,306		Basal vs. Normal

*univariate analysis

variable	HR	95%CI	p-value	n	groups
**CPT1A**	**1,22**	**1.05-1.43**	**0,009**	**1227(710)**	**median**
age	1,60	1.31-1.97	<.0001		<50 vs. >=50
stage	1,92	1.50-2.47	<.0001		1-2 vs. 3-4
subtype	0,87	0.64-1.19	0,3786		LumA vs. Normal
	1,12	0.81-1.54	0,4995		LumB vs. Normal
	1,43	1.01-2.02	0,0457		Her2 vs. Normal
	1,18	0.82-1.70	0,3612		Basal vs. Normal

*multivariate analysis: model, OS=CPT1A+age+stage+subtype

*still sig after correct for menopausal

METABRIC

continuous

variable	HR	95%CI	p-value	n	groups
**CPT1A**	**1,51**	**1.34-1.71**	**<.0001**	**1904(1103**	**continuous**
age	1,68	1.43-1.98	<.0001	1904(1103	<50 vs. >=50
stage	1,81	1.62-2.03	<.0001	1403(792)	0,1,2,3,4
subtype	0,86	0.67-1.11	0,2444	1699(1009	LumA vs. Normal
	1,30	1.01-1.67	0,0396		LumB vs. Normal
	1,47	1.12-1.94	0,0059		Her2 vs. Normal
	1,16	0.87-1.56	0,306		Basal vs. Normal

*univariate analysis

variable	HR	95%CI	p-value	n	groups
**CPT1A**	**1,17**	**0.99-1.37**	**0,058**	**1227(710)**	**continuous**
age	1,61	1.32-1.98	<.0001		<50 vs. >=50
stage	1,90	1.48-2.43	<.0001		1,2,3,4
subtype	0,88	0.64-1.19	0,4001		LumA vs. Normal
	1,12	0.81-1.54	0,4881		LumB vs. Normal
	1,44	1.02-2.05	0,0399		Her2 vs. Normal
	1,18	0.82-1.70	0,3601		Basal vs. Normal

*multivariate analysis: model, OS=CPT1A+age+stage+subtype

*still sig after correct for menopausal

SUPPLEMENTARY TABLE 5

Primers used to analyze mRNA levels

Target organism	Target gene	Sequence 5' – 3'
Human	*RPL19*	Fwd: ATTGGTCTCATTGGGGTCTAAC<br>Rev: AGTATGCTCAGGCTTCAGAAGA
Human	*CPT1A*	Fwd: GCACCTCCGTAGCTGACTC<br>Rev: GAGTGACCGTGAACTGAAAGG
Human	*ICAM1*	Fwd: GGCTGGAGCTGTTTGAGAAC<br>Rev: CTGTGGGGTTCAACCTCTG
Human	*PTPN13*	Fwd: TTCTCTGCAGACCTCCACCT<br>Rev: TCTTCTCCACTCCCACTGCT
Human	*TNFAIP2*	Fwd: GAAGTCTGGCTGAGGTCTGG<br>Rev: CTCCAGAAGGAGTGCAGGAC
Human	*MMP9*	Fwd: TTGACAGCGACAAGAAGTGG<br>Rev: GCCATTCACGTCGTCCTTAT
Human	*JAG1*	Fwd: TGCTACAACCGTGCCAGTGACT<br>Rev: TCAGGTGTGTCGTTGGAAGCCA
Mouse	*Cpt1a*	Fwd: CAGAGGATGGACACTGTAAAGG<br>Rev: AGTATGCTCAGGCTTCAGAAGA
Mouse	*Rpl19*	Fwd: CAGGCATATGGGCATAGGGAA<br>Rev: TGCCTTCAGCTTGTGGATGT
Mouse	*Icam1*	Fwd: AGCACCTCCCCACCTACTTT<br>Rev: AGCTTGCACGACCCTTCTAA
Mouse	*Ptpn13*	Fwd: GAACACCTCGACTGTGCTGA<br>Rev: GGACGCTGGTATTCACACCT
Mouse	*Tnfaip2*	Fwd: AAAAAGGACCAGCCCAGATT<br>Rev: TACAGAGCCTCCACCTTGCT
Mouse	*Mmp9*	Fwd: TGAATCAGCTGGCTTTTGTG<br>Rev: GTGGATAGCTCGGTGGTGTT
Mouse	*Fabp5*	Fwd: GACGACTGTGTTCTCTTGTAACC<br>Rev: TGTTATCGTGCTCTCCTTCCCG
Mouse	*Sfpb*	Fwd: TGTGCCAAGAGTGTGAGGAT<br>Rev: CAGGGGCAGGTAGACATCAA
Mouse	*Sfpc*	Fwd: GATGAGAAGGCGTTTGAGGT<br>Rev: GATGAGAAGGCGTTTGAGGT
Mouse	*Abca3*	Fwd: GGTCCTGATGGAGAGTCCAC<br>Rev: GGAGCAGGAACGCTGAGAT
Mouse	*Acsl4*	Fwd: CCTTTGGCTCATGTGCTGGAAC<br>Rev: GCCATAAGTGTGGGTTTCAGTAC
Mouse	*Kat2a*	Fwd: CACGGAAATCGTCTTCTGTGCC<br>Rev: CGTACTCGTCAGCATAGGTGAG
Mouse	*Kat2b*	Fwd: CCTCTTCACCTGCGTCCACAAA<br>Rev: TCTCCAAGGAGCCTTCAACCAC
Mouse	*Noa1*	Fwd: GAGCGGCATAAAATTCTGCACCG<br>Rev: CACTGACACAGCAGTAGAGGCT
Mouse	*Kat5*	Fwd: TGAGCGTGAAGGACATCAGTGG<br>Rev: TTAAGTCCAGCCGCTCGTGAGT
Mouse	*Kat6a*	Fwd: CGGTCAAACTCGCCACCAATTC<br>Rev: CTAACACCTCCGTGGTCTCAGA
Mouse	*Kat6b*	Fwd: GCTGTGGTTTCTGAGGAAGAGC<br>Rev: TGCCTACTGCTAACTCTGGACG
Mouse	*Ep300*	Fwd: GTGATGACCCTTCCCAACCTCA<br>Rev: CTCGTGGTGAAGGACACAGATC
Mouse	*Crebbp*	Fwd: CACCATCTGTGGCTACTCCTCA<br>Rev: GGTTTCAGCACTGGTCACAGAG
Mouse	*Kat7*	Fwd: AGGAAAAGGTGGCTGAACTCAGG<br>Rev: GTCAGGTTTTCCAAGAGAGGCTC
Mouse	*Kat8*	Fwd: CAGCAGAAGTGATCCAGTCTCG<br>Rev: TTGGTCAGTGCGAGTCGGTTCT
